# Host Stress Drives *Salmonella* Recrudescence

**DOI:** 10.1038/srep20849

**Published:** 2016-02-09

**Authors:** Elin Verbrugghe, Maarten Dhaenens, Bregje Leyman, Filip Boyen, Neil Shearer, Alexander Van Parys, Roel Haesendonck, Wim Bert, Herman Favoreel, Dieter Deforce, Arthur Thompson, Freddy Haesebrouck, Frank Pasmans

**Affiliations:** 1 Department of Pathology, Bacteriology and Avian Diseases, Faculty of Veterinary Medicine, Ghent University, Merelbeke, Belgium; 2 Laboratory for Pharmaceutical Biotechnology, Faculty of Pharmaceutical Sciences, Ghent University, Ghent, Belgium; 3 Gut Health and Food Safety Programme, Institute of Food Research, Norwich Research Park, Norwich NR4 7UA, United Kingdom; 4 Department of Biology, Nematology Research Unit, Faculty of Sciences, Ghent University, Ghent, Belgium; 5 Department of Virology, Parasitology and Immunology, Faculty of Veterinary Medicine, Ghent University, Merelbeke, Belgium

## Abstract

Host stress is well known to result in flare-ups of many bacterial, viral and parasitic infections. The mechanism by which host stress is exploited to increase pathogen loads, is poorly understood. Here we show that *Salmonella enterica* subspecies *enterica* serovar Typhimurium employs a dedicated mechanism, driven by the *scsA* gene, to respond to the host stress hormone cortisol. Through this mechanism, cortisol increases *Salmonella* proliferation inside macrophages, resulting in increased intestinal infection loads in DBA/2J mice. *ScsA* directs overall *Salmonella* virulence gene expression under conditions that mimic the intramacrophagic environment of *Salmonella*, and stimulates the host cytoskeletal alterations that are required for increased *Salmonella* proliferation inside cortisol exposed macrophages. We thus provide evidence that in a stressed host, the complex interplay between a pathogen and its host endocrine and innate immune system increases intestinal pathogen loads to facilitate pathogen dispersal.

Apt responses to stressors promote an organism’s chance of survival as they prepare the body for the innate fight-or-flight response^1^. During periods of stress, hormones are released into the circulation or tissues in order to mediate the stress response. The resulting increase in circulating stress hormones such as epinephrine, norepinephrine and cortisol, however, coincides with increased susceptibility to infectious diseases, resulting in recrudescence of viral^2^, parasitic^3^ or bacterial diseases^4^. These flare-ups are commonly ascribed to general effects on the overall host immune status disrupting the host-pathogen equilibrium. Evidence is however growing that pathogens, such as *Salmonella*, can exploit the neuroendocrine alteration due to a stress reaction as a signal for growth and pathogenic processes^4^,^5^ and by doing so, promote pathogen survival through increased dispersal in the host or to novel hosts^5^,^6^.

Depending on the *Salmonella* strain and host, salmonellosis, one of the globally most important zoonotic bacterial diseases, can result in (self-limited) gastroenteritis, septicemia and even death. Worldwide, typhoidal^7^ and nontyphoidal^8^ *Salmonella* infections result in an estimated 20 million and 98.3 million human cases each year, of which 200, 000 and 155, 000 result in death. As such, *Salmonella* infections still pose major public health concerns worldwide. The pathogen is notorious for its ability to cause persistent infections both in humans, with the most notable example being typhoid Mary^9^, and a number of animal species, such as poultry and pigs^4^. During periods of host stress, *Salmonella* is able to sense stress hormones and to exploit their presence to its advantage, increasing the pathogen’s likeliness of dispersal and thus its chances of successful maintenance in the host population^5^. Both catecholamines^10^,^11^ and glucocorticoids^5^ have been suggested to contribute to stress-induced recrudescence of a *Salmonella* infection. Recently, we showed that glucocorticoids such as cortisol and dexamethasone, promote *Salmonella* proliferation inside porcine macrophages and *Salmonella* infections in pigs, representing a prime example for stress-induced flare-up of infections^5^.

Until now, the underlying mechanism of these stress-induced flare-ups is poorly understood. Therefore, the aim of the present study was to unravel the mechanism of how the infected macrophages respond to cortisol exposure and to identify *Salmonella* Typhimurium genes responsible for the cortisol-induced increased survival of the bacterium. Our study reveals that the *scsA* gene drives glucocorticoid-induced recrudescence of *Salmonella* inside host macrophages by altering bacterial virulence gene expression, which eventually results in recrudescence of infection.

## Results and Discussion

### Cortisol induces the macrophage cytoskeletal rearrangements that facilitate intracellular bacterial replication

**Figure 1:**
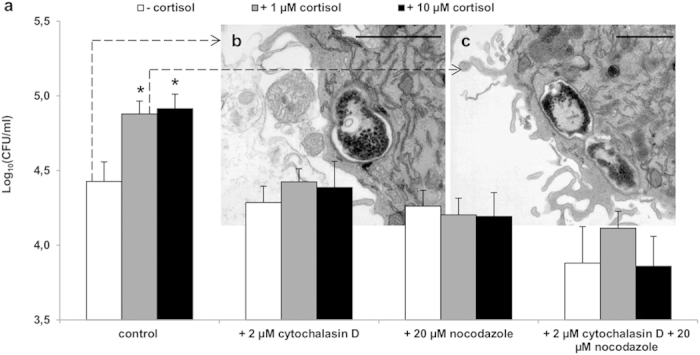
Cytoskeletal rearrangements in porcine macrophages are required for increased intracellular proliferation of *Salmonella*. (**a**) Number of intracellular *Salmonella* Typhimurium bacteria in porcine macrophages that were treated with unsupplemented medium, 2 μM cytochalasin D (inhibitor of F-actin polymerization), 20 μM nocodazole (inhibitor for microtubule formation) or the combination of both, for 24 hours after invasion. White bars illustrate medium without cortisol, grey bars represent medium with 1 μM cortisol and black bars indicate medium with 10 μM cortisol. An asterisk (*) refers to a significant difference compared to the respective controls without cortisol (P ≤ 0.05). Panels (**b,c**) represent TEM images, 6 hours after control (**b**) or 1 μM cortisol (**c**) treatment of *Salmonella* Typhimurium WT-infected porcine macrophages, showing SCV formation (scale bar, 1 μm).

We demonstrated that the cortisol-induced increased proliferation of *Salmonella* is due to glucocorticoid-induced host cytoskeletal changes that result in the formation of newly formed bacterial replicative niches^12^,^13^,^14^, the *Salmonella*-containing vacuoles (SCVs). During increased replication of *Salmonella* Typhimurium in cortisol exposed macrophages, the SCV was shown to divide together with the bacterium, resulting in a single bacterium per SCV (Fig. 1c). This process of increased SCV production required F-actin polymerization and microtubule formation (Fig. 1a) and did not coincide with obvious cortical actin redistribution (Supplementary Fig. S1). Proteomic and transcriptomic analyses confirmed the cortisol-induced increased expression of several cytoskeleton-associated proteins in *Salmonella*-infected macrophages, including tubulin beta chain (TUBB2A), F-actin capping protein subunit 2 (CAPZB), thymosin beta-4 (TMSB4X), actin-related protein 3B (ACT3), and tropomyosin 3 (TPM3) (Supplementary Table S1 and Supplementary Fig. S2). We thus conclude that cortisol induces cytoskeletal rearrangement activities in *Salmonella*-infected macrophages that underpin the formation of novel SCVs and thus allow increased bacterial replication.

### Cortisol increased intracellular replication is driven by *scsA*

**Figure 2:**
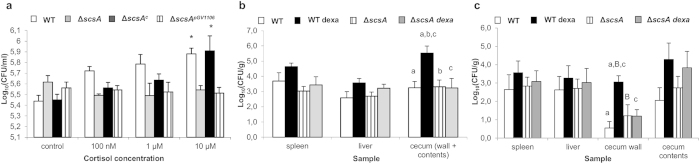
*ScsA* mediates cortisol-induced increase in proliferation of *Salmonella*, both *in vitro* and *in vivo*. (**a**) Shown is the effect of cortisol on the log_10_ values + standard deviation of intracellular *Salmonella* Typhimurium WT, Δ*scsA,* Δ*scsA*^c^ and Δ*scsA*^pGV1106^ bacteria in porcine macrophages. An asterisk (*) refers to a significant difference compared to the condition without cortisol (P ≤ 0.05). Panels (**b,c**) illustrate the effect of dexamethasone exposure on the recovery of *Salmonella* WT and Δ*scsA* from organs of DBA/2J mice. The log_10_ value of the CFU/gram sample is given as the mean + standard error of the mean. Significant differences are signed with a, b, c (P ≤ 0.017) and a tendency with B (P ≤ 0.033).

**Figure 3:**
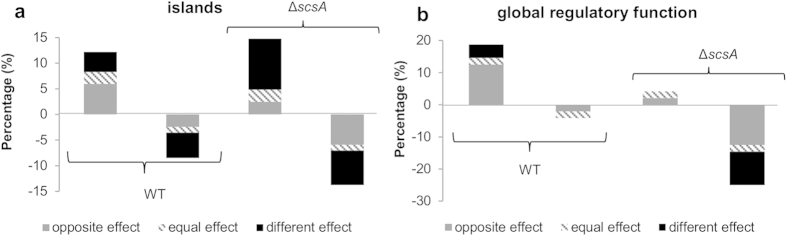
Cortisol effect on gene expression during intracellular environment mimicking conditions. For both WT and Δ*scsA* strain, the percentage of genes up or down regulated by cortisol is shown quantitatively compared to the total number of genes belonging to the (**a**) islands (894 genes) or (**b**) regulatory function (48 genes) group. Grey bars represent the percentage of genes of which the cortisol effect in WT is the opposite in Δ*scsA* strain. Striped bars reflect the genes that act similar after cortisol treatment. Black bars depict the genes that are up or down regulated after cortisol treatment in WT but not in Δ*scsA*, or vice versa. Microarray data have been deposited in the Gene Expression Omnibus at NCBI with series accession numbers GSE55430.

Using *in vivo* expression technology^15^ (IVET; Supplementary Table S2) and intracellular proliferation tests (Fig. 2a), we identified the *Salmonella* gene *scsA* as the key driver for cortisol-induced intracellular replication of *Salmonella* in porcine and murine macrophages (Fig. 2a and Supplementary Fig. S3a). Deletion of *scsA* abolished this increase in proliferation, an effect that was restored by the complementation of *scsA (*Δ*scsA*^*c*^) (Fig. 2a). We then explained the crucial contribution of the *scsA* gene by its key role in the cortisol-induced dramatic alteration of *Salmonella* gene expression under phagosome mimicking conditions (Supplementary Table S3 and Fig. 3). Indeed, under these conditions, cortisol altered the expression of more than 20% of *Salmonella* genes involved in global regulation and pathogenicity islands, both key groups for the bacterium’s virulence (KEGG database). Deletion of the *scsA* gene largely disturbed this cortisol-induced expression pattern (Fig. 3). The *scsA* gene was shown to directly or indirectly mediate the cortisol-induced increased expression of several major systems that are indispensable for intramacrophage survival: SPI-2 associated proteins (*sscA* chaperone, *sseI* effector and *ssaG* structural protein); *phoP*, belonging to the PhoQ/PhoP two-component regulatory system^16^; and the sigma factors σ^S^ (*rpoS*) and σ^E^ (*rpoE*)^17^,^18^. The involvement of *scsA* in the cytoskeletal rearrangements, required for intracellular replication, was shown by its impact on the expression of *Salmonella* genes involved in host cytoskeletal reformations (*sipC*)^19^ and SCV/*Salmonella*-induced filaments formation (*pipB*)^20^ (Supplementary Table S3) and by its impact on the expression of the host actin-related protein ACT3 (Supplementary Fig. S2).

### *ScsA* mediates the glucocorticoid-induced increase of macrophage-associated *Salmonella* loads in the cecal wall of DBA/2J mice

We then demonstrated the crucial role of both *scsA* and macrophages in glucocorticoid-driven *Salmonella* Typhimurium recrudescence in a mouse model of infection. Previously, it was shown that a single dexamethasone stimulus can be used to mimic stress-related flare-up of a *Salmonella* infection in pigs^5^. We now first optimized a model in DBA/2J mice to reproduce dexamethasone-induced *Salmonella* proliferation. As shown in Supplementary Fig. S4, organs of *Salmonella*-infected DBA/2J mice that were subsequently injected with dexamethasone showed significantly higher numbers of *Salmonella* Typhimurium bacteria, compared to DBA/2J mice that were injected with a saline solution. In BALB/c mice, no significant differences were observed in the *Salmonella* load between dexamethasone and HBSS-injected animals.

**Figure 4:**
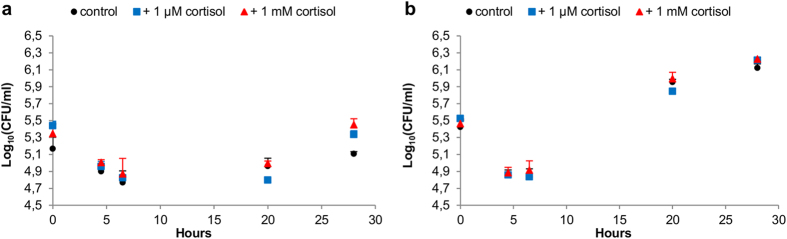
Effect of cortisol on the growth of *Salmonella* Typhimurium in cecal contents. The log_10_ values of the CFU/ml + standard deviation are given at different time points (t = 0, 4.5, 6.5, 20, 28 hours under microaerobic conditions at 37 °C) after inoculation in cecal contents. *Salmonella* Typhimurium (**a**) WT and (**b**) Δ*scsA* growth was examined in cecal contents with (1 μM or 1 mM) or without cortisol.

**Figure 5:**
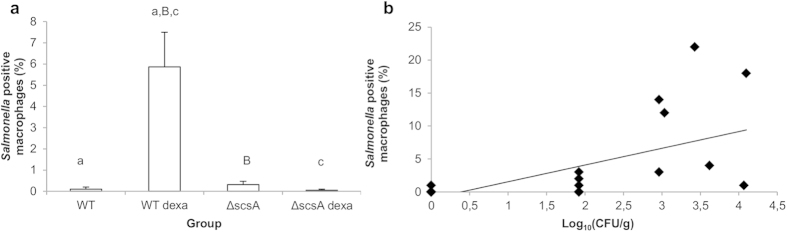
Effect of glucocorticoids on the number of *Salmonella* positive macrophages. Panel (**a**) shows the effect of dexamethasone exposure on the colocalization of *Salmonella* WT and Δ*scsA* with F4/80+ cells in the cecal wall of DBA/2J mice. Given is the percentage of the *Salmonella* positive macrophages + standard error of the mean compared to the total number of counted macrophages. Significant differences are signed with a, c (P ≤ 0.017) and a tendency with B (P ≤ 0.033). Panel (**b**) indicates a significant (P ≤ 0.05) positive correlation between the percentage of *Salmonella* positive macrophages per mouse and their respective log_10_ value of the CFU/gram cecal wall (correlation coefficient = 0.62).

**Figure 6:**
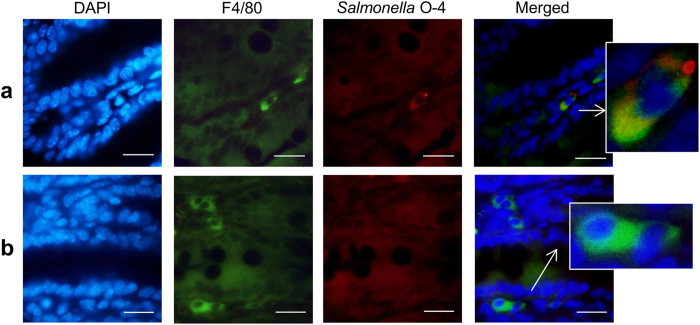
*Salmonella* colocalization with F4/80+ cells in the cecum. Cecal sections of *Salmonella*-infected DBA/2J mice were stained for immunofluorescence using an F4/80 antibody targeting macrophages (green) and a *Salmonella* O–4 antibody (red). Nuclei were stained with DAPI (blue). Shown are representative images demonstrating (**a**) colocalization of *Salmonella* and F4/80+ cells or (**b**) *Salmonella* negative F4/80+ cells (scale bar, 20 μm).

Using this DBA/2J model, we then demonstrated that *scsA* is required for the glucocorticoid-induced increase in *Salmonella* infection load in the murine cecum (wall + contents) *in vivo* (Fig. 2b). In a second experiment, we showed that the *scsA*-dependent increase of intestinal *Salmonella* Typhimurium loads coincides with higher numbers of *Salmonella* Typhimurium positive macrophages in the cecal wall, but not with intraluminal proliferation of *Salmonella* Typhimurium bacteria (Figs 2c, 4, 5 and 6), which is in agreement with macrophages being the main host cells for *Salmonella* in the murine cecum^21^. No such phenomenon was observed in spleen and liver, strengthening the hypothesis that the *scsA*-dependent increase of *Salmonella* is tissue specific and limited to the intestinal tract. Previous published work by Van Parys *et al.* (2011) indeed showed that *scsA* gene expression is relevant in the gut wall, but not in the tonsils and ileocaecal lymph nodes of pigs^15^. Moreover, our findings are in agreement with previously published work^5^ in which glucocorticoids significantly increased the number of *Salmonella* bacteria in the intestinal wall (ileum, cecum and colon) of pigs. Using DBA/2J mice, which are considered intermediately susceptible to *Salmonella* Typhimurium infections, we were able to mimic stress-related increased replication of *Salmonella* Typhimurium in a persistently infected host. The infection dynamics in this model are similar to the course of a *Salmonella* infection in pigs, which is characterized by an acute phase around day 3–5 post infection (p.i.) and the development of a chronic infection once the acute phase is controlled^22^. As such, our results demonstrate a novel bacterial mechanism that senses host stress, resulting in a flare up of bacterial loads, being of great importance for food-producing animals like pigs. From an epidemiological point of view, this is highly relevant to promote sustaining the infection at population level.

### *ScsA* is involved in *scsBCD* regulation

The *scsA* gene is part of a *scs* locus, consisting of two operons, a first one consisting of a single gene (*scsA*) and a second operon consisting of three genes (*scsB*-*C*-*D*)^23^. The *scs* genes encode for proteins with a predicted *Cys*-X-X-*Cys* motif and an enriched containment of hydrophobic amino acids between the *Cys* residues, being characteristic for the thioredoxin superfamily^24^. The Scs proteins also show homology with Dsb proteins of *Escherichia coli*, being disulfide bond formation proteins that are involved in the correct folding of target proteins by catalyzing disulfide bond isomerization^25^. Many virulence factors are secretion proteins that are posttranslationally modified via disulfide bond formation and inactivation of genes involved in the Dsb system leads to reduced virulence in many pathogens^25^. Recently, the role of ScsB as an electron transporter was confirmed and ScsB proteins were described as a new class of DsbD proteins^26^. Therefore, we hypothesize that the *scsA* gene is also involved in the formation of disulfide bonds and correct folding of proteins. Using microarray analysis, we showed that deletion of the *scsA* gene results in the upregulation of the *scsBCD* operon (Supplementary Table S4), suggesting that *scsA* acts as a regulator for the *scsBCD* operon. Gupta *et al.* (1997) stated that expression of both operons is required for the correct functioning of the *scs* locus. As such, deletion of *scsA* probably results in the incomplete and impaired operation of this locus, which could lead to impaired virulence.

We conclude that the *scsA* gene-driven glucocorticoid-induced proliferation of *Salmonella* inside host macrophages results in increased intestinal infection loads that facilitate dispersal to novel hosts. These results oppose the general idea that stress-associated exacerbation of infectious diseases merely is the result of lowered general host immune status. Rather, the acquisition of a dedicated virulence mechanism to increase chances of escape from a stressed and possibly less fit host should be considered an evolutionary advantage, which promotes pathogen maintenance in the host population.

## Methods

### Bacterial strains

*Salmonella* Typhimurium strain 112910a was used as the wild type strain (WT), in which the spontaneous nalidixic acid resistant derivative strain (WT ^nal^) was constructed^27^. *Salmonella* Typhimurium Δ*scsA* deletion mutant was constructed according to the one-step inactivation method described by Datsenko and Wanner^28^ and slightly modified for use in *Salmonella* Typhimurium as described previously^29^. Gene complementation of the deletion mutant Δ*scsA* was constructed using the vector plasmid pGV1106^30^. The resulting complemented deletion mutant is further abbreviated as Δ*scsA*^c^. As a control, we electroporated the empty pGV1106 plasmid into the deletion mutant, hereafter named Δ*scsA*^pGV1106^. Primers used to create the gene-specific linear PCR fragment for gene deletion (*scsA* forward and reverse) and complementation (*scsA*^c^ forward and reverse) are given in Supplementary Table S5. *Salmonella* Typhimurium WT and Δ*scsA* expressing green fluorescent protein (GFP) were used for confocal laser scanning microscopy (CLSM).

### Infection experiments in cell cultures

Porcine alveolar macrophages (PAM) were isolated and cultured as previously described^5^. PAM were seeded in 175 cm^2^ cell culture flasks (5 × 10^7^ cells) for the proteome analysis, in 24-well plates (1 × 10^6^ cells) for the invasion and proliferation assays, on a cover slip (1 × 10^6^ cells) for transmission electron microscopy (TEM) and CLSM, and in 6-well plates (3 × 10^6^ cells) for the IVET experiment and RNA extraction. The cells were infected with *Salmonella* Typhimurium at a multiplicity of infection (MOI) of 10:1, with the exception for TEM and IVET (50:1). To synchronize the infection, the inoculated plates or flasks were centrifuged at 365 × g for 10 min and incubated for 30 min at 37 °C under 5% CO_2_. After washing the cells, extracellular bacteria were treated with gentamicin (100 μg/ml) for 1 hour. To assess the intracellular proliferation of *Salmonella*, the medium containing 100 μg/ml gentamicin was replaced after 1 hour incubation with fresh medium containing 20 μg/ml gentamicin, supplemented with cortisol when required.

### Proteome analysis

To map out the effects of cortisol-induced increased survival of *Salmonella* Typhimurium on the protein expression of host cells, iTRAQ analysis was performed. Twenty-four hours after infection, protein extraction, digest, labeling, nano LC-MSMS analysis and data analysis of the samples, were performed as previously described^31^. The experiment was conducted in duplicate and the labeling of the samples was as follows: run 1 (untreated PAM sample 1: 114 – untreated PAM sample 2: 115 – cortisol treated PAM sample 1: 116 – cortisol treated PAM sample 2: 117) – run 2 (untreated PAM sample 3: 114 – untreated PAM sample 4: 115 – cortisol treated PAM sample 3: 116 – cortisol treated PAM sample 4: 117).

### Role of F-actin and microtubuli

To assess the intracellular proliferation of *Salmonella*, the medium containing 100 μg/ml gentamicin was replaced after 1 hour incubation with fresh medium containing 20 μg/ml gentamicin, with or without 1 μM or 10 μM cortisol, 2 μM cytochalasin D (inhibitor of F-actin polymerization) and/or 20 μM nocodazole (inhibitor for microtubule formation). Twenty-four hours after infection, the number of viable bacteria was determined by plating 10-fold dilutions on brilliant green agar (BGA).

### TEM

PAM cells previously infected with *Salmonella* Typhimurium parental strain (in the absence or presence of 1 μM cortisol) were analyzed at 2, 6 and 16 hours after infection. TEM was performed with glutaraldehyde fixation in 0.05 M sodium cacodylate buffer, 1% osmium tetroxide postfixation, and en bloc staining for 1 h in a 1% solution of uranyl acetate.

### CLSM

Sixteen hours after infection and cortisol treatment, PAM were fixed, permeabilized and stained with phalloidin-Texas Red as previously described^32^. Nuclei were stained with 4′,6′-diamidino-2-phenylindole (DAPI) (Vector).

### IVET screening

An IVET transformants pool was used, covering the major part of the *Salmonella* Typhimurium genome, to identify *Salmonella* Typhimurium genes that are intracellularly expressed in PAM after exposure to cortisol. The IVET pool was constructed by the use of a pIVET1 plasmid as previously explained^15^. Shortly, the pIVET1 plasmid is a derivate of the suicide vector pGP704 and contains a promoterless synthetic operon of *purA* coupled to *lacZY*, preceded by a *Bgl*II site. *Salmonella* Typhimurium genomic DNA was purified and subsequently digested with *Sau3AI*, resulting in a library of 1–4 kb overlapping genomic DNA fragments. These fragments were cloned in the *Bgl*II site of the pIVET1 plasmid, upstream to promoterless wild type copies of *purA* and *lacZY*. After transferring the plasmids to *Escherichia coli* DH5αpir, approximately 100 000 clones were pooled. Using a Plasmid Midi Kit (Qiagen), the pIVET1 fusion plasmids were isolated and electroporated in the conjugative *E. coli* SM10 λpir and again, approximately 1,00, 000 different *E. coli* SM10 λpir clones were pooled. These fusion plasmids were then mobilized into a *Salmonella* Typhimurium Δ*purA* strain by conjugation using the *E. coli* SM10 λpir as donor strain. PurA encodes for adenylosuccinate synthetase, an enzyme which is essential for the synthesis of adenosine monophosphate. The wild type locus of the gene was not disrupted since the integration of the pIVET1 plasmid in the chromosome resulted in a single cross over. Finally, *Salmonella* Typhimurium Δ*purA* transformants were obtained, in which te native promoter drives the expression of the *purA-lacZY* fusion, while the cloned promoter drives the expression of the wild type gene. The *Salmonella* Typhimurium Δ*purA* strain is severely attenuated in pigs^15^ and in macrophages (Supplementary Fig. S3b). This means that if the cloned DNA contains a promoter which is activated intracellularly in macrophages, the *purA* gene and the *lacZY* operon will be expressed and the bacterium will survive. If the cloned DNA fragment does not contains a promoter, or a promoter that is not activated intracellularly, no adenosine monophosphate will be synthsized and the bacterium will be severely attenuated.

The *Salmonella* Typhimurim IVET pool was inoculated into PAM and the infected cells were treated with medium with or without 1 μM cortisol. Sixteen hours after infection, PAM were washed and lysed for 10 min with 500 μl 1% Triton X-100. This was added to 9.5 ml of LB broth enriched with the additives, 50 μg/ml ampicillin, 20 μg/ml nalidixic acid, 1.35% adenine and 0.337% thiamine and grown with aeration at 37 °C. After 3 hours, the bacterial culture was centrifuged at 2300 x g for 10 min at 37 °C and the pellet was resuspended in 3 ml PAM medium without antibiotics. This was considered as one passage and in total three passages were performed. Finally, the cells were lysed and plated on MacConkey agar supplemented with the additives and 1% filter-sterilized lactose to assess the *lacZY* expression level of the IVET transformants. This allowed detection of IVET transformants containing promoters expressed intracellularly in PAM but not on MacConkey agar. These fusion strains formed white to pink colonies on MacConkey lactose agar (low-level *lacZY* expression), whereas fusion strains containing promoters that are constitutively expressed showed red colonies (high-level *lacZY* expression). As we were interested in genes that are intracellularly induced, but not extracellularly, all the colonies showing low-level *lacZY* expression were picked up, purified and sequenced as previously described^15^.

### Invasion and proliferation assays

Based on literature and IVET screening, the effect of cortisol on the intracellular proliferation of Δ*scsA* and Δ*scsA*^*c*^ was determined in comparison to the WT strain. Since *Salmonella* Typhimurium Δ*scsA*^c^ is resistant to kanamycin, polymyxin B was used instead of the commonly used gentamicin to kill the extracellular *Salmonella* bacteria. The medium containing 5 μg/ml polymyxin B was replaced after 1 hour incubation with fresh medium containing 5 μg/ml polymyxin B with or without cortisol ranging from 0,1–10 μM. After 24 hours, the cells were washed 3 times, lysed and 10-fold dilutions were plated on BGA plates.

### Microarray analysis

The effect of cortisol exposure (1 μM) on *Salmonella* gene expression was analyzed using RNA isolated from parental and Δ*scsA* strains grown to logarithmic phase in a medium that reflects the intracellular environment. The *Salmonella* strains were grown to logarithmic phase in a low pH minimal medium (MM 5.8), which was slightly modified to achieve a maximal SPI-2 expression, as previously described^33^. The analysis was performed as previously described^5^.

### Analysis of eukaryotic mRNA expression

Sixteen hours after cortisol treatment, total RNA was isolated using TRI Reagent^®^ combined with the RNeasy mini kit (Qiagen) according to the manufacturer’s guidelines. RNA concentration was measured by absorbance at 260 nm using Nanodrop spectrophotometer and purity of the RNA samples was checked using an Experion RNA StdSens Analysis kit. Total RNA (500 ng) was reverse transcribed to cDNA with the iScript cDNA synthesis kit^34^. Primers used for the amplification were designed using Primer3 software. Specificity of the primers was tested by performing a BLAST search against the genomic NCBI database. The list of genes and sequences of the primers used for quantitative PCR analysis can be found in Supplementary Table S5. HIS^34^ and BLM^35^ were used as housekeeping genes. All reference genes had a stable expression, in all the samples tested, as calculated using the geNorm software. Real-time quantitative PCR reactions were performed as earlier described^34^. For each condition, RNA was extracted from three replicates and data were collected from two independent experiments. Real-time quantitative PCR reactions were run in duplicate.

### Ethics statement

All animal experiments were carried out in strict accordance with the recommendation in the European Convention for the Protection of Vertebrate Animals used for Experimental and other Scientific Purposes. The experimental protocols and care of the animals were approved by the Ethical Committee of the Faculty of Veterinary Medicine, Ghent University (EC2011/116 and EC2012/160).

### Optimization of a stress mimicking mouse model

The *scsA*-dependent increased intracellular replication of *Salmonella* Typhimurium was also observed in murine macrophages (RAW cells; Supplementary Fig. S3a). Therefore, a mouse model was developed that mimicks stress-related increased replication of *Salmonella* Typhimurium. It was evaluated whether dexamethasone increases the number of *Salmonella* Typhimurium bacteria in the gut of *Salmonella*-infected mice, in order to create a mouse model allowing screening of bacterial genes that might be involved in dexamethasone-induced enhanced replication of *Salmonella*. Depending on the bacterial strain and host, a *Salmonella* infection can vary from a (self-limited) gastrointestinal infection, to septicemia and even death. To mimick these differences in disease outcome, we tested the effects of glucocorticoids in two different mouse models. DBA/2J mice are intermediately sensitive to *Salmonella* Typhimurium infections, resulting in carrier animals, whereas BALB/c mice are highly susceptible to *Salmonella* Typhimurium infections^36^. Eighteen, four-week-old DBA/2J mice and eighteen, four-week-old BALB/c mice, were housed in filter-topped cages at 25 °C under natural day-night rhythm with *ad libitum* access to feed and water and enriched with mouse houses and play tunnels. Five days after arrival, all mice were infected with a total of 1 × 10^6^ CFU of *Salmonella* Typhimurium WT ^nal^ by the orogastric route. At day 7 p.i. six BALB/c mice were subcutaneously injected once with 100 mg/kg dexamethasone. Simultaneously, six BALB/c mice received a subcutaneous injection of 25 mg/kg dexamethasone, which was repeated after three hours. Fourteen days p.i. six DBA/2J mice were subcutaneously injected once with 100 mg/kg dexamethasone and simultaneously six DBA/2J mice received a subcutaneous injection of 25 mg/kg dexamethasone (repeated after three hours). Six mice of each strain received a subcutaneous injection of 200 μl HBSS- (24 hours before euthanasia) and were used as control group. Twenty-four hours after the last injection of dexamethasone, all animals were humanely euthanized and samples of spleen, liver and cecum were collected. Samples were processed and the number of *Salmonella* bacteria was determined as previously described^5^. The detection limit for direct plating was 83 CFU/gram tissue or contents.

### Role of *scsA* during the glucocorticoid response of *Salmonella in vivo*

The DBA/2J mouse model was used to verify whether *scsA* plays an essential part in the glucocorticoid-related multiplication *in vivo*. Three- to four-week-old DBA/2J mice were used and randomly allocated in two groups of sixteen mice. The animals were housed in filter-topped cages at 25 °C under natural day-night rhythm with ad libitum access to feed and water and enriched with mouse houses and play tunnels. After an acclimatization period of one week, mice were inoculated with a total of 1 × 10^6^ CFU of *Salmonella* Typhimurium WT ^nal^ or its isogenic *scsA* knock-out mutant. At day 14 p.i., eight animals of each group were subcutaneously injected with 100 mg/kg dexamethasone and eight mice were subcutaneously injected with 200 μl HBSS- and served as a control group. Twenty-four hours later, all mice were humanely euthanized. Spleen, liver and cecum (wall + contents) samples were examined for the number of *Salmonella* Typhimurium bacteria. Samples were processed and the number of *Salmonella* bacteria was determined as previously described^5^. The detection limit for direct plating was 83 CFU/gram tissue or contents.

### Contribution of *scsA* to glucocorticoid-dependent intestinal *Salmonella* loads *in vivo*

The *in vivo* experiment described above, showed that *Salmonella* responds to glucocorticoids in an *scsA*-dependent way, with increased intestinal loads as a result. In this experiment we investigated whether this results from an increased number of intramural *Salmonella* containing macrophages, or increased extracellular proliferation in the intestinal contents. Therefore, 35 four-week-old DBA/2J mice were randomly divided in 5 groups of 7 mice. Per group, the animals were distributed over 3 filter-topped cages. One cage contained 3 mice and the two other cages comprised 2 mice. After an acclimatization period, one group was inoculated with HBSS- and served as a negative control group, two groups were inoculated with 1 × 10^6^ CFU of *Salmonella* Typhimurium WT^nal^ and two groups were infected with 1 × 10^6^ CFU of *Salmonella* Typhimurium Δ*scsA*. At day 14 p.i., three mice of the control group, seven *Salmonella* Typhimurium WT^nal^- and seven *Salmonella* Typhimurium Δ*scsA*-infected mice were subcutaneously injected with 100 mg/kg dexamethasone. The other mice were subcutaneously injected with 200 μl HBSS-. Twenty-four hours later, all mice were humanely euthanized. Spleen, liver, cecum and cecum contents were examined for the number of *Salmonella* Typhimurium bacteria. Samples were processed and the number of *Salmonella* bacteria was determined as previously described^5^. The detection limit for direct plating was 83 CFU/gram tissue or contents. Samples of the cecum were fixed in 4% phosphate buffered formaldehyde for immunofluorescence. They were processed by standard methods, and embedded in paraffin.

### Immunofluorescence of cecal sections

Cecal sections were de-paraffinized in xylene and hydrated through a graded series of alcohols. Heat-mediated antigen retrieval using citrate buffer (pH 6.0) was included to achieve optimum staining. After washing three times for 5 minutes with phosphate-buffered saline (PBS), the samples were incubated for 15 minutes with PBS supplemented with 0.3% Triton and 2% goat serum, washed three times with PBS and incubated with PBS supplemented with 10% goat serum, for 30 minutes. To prevent non-specific binding, the sections were subsequently incubated for 2 hours with an unconjugated Fab fragment goat anti-mouse IgG (200 μg/ml; Jackson ImmunoResearch Labs). After washing three times for 20 minutes, the samples were incubated for 90 minutes with a rabbit polyclonal antibody raised against a peptide mapping at the C-terminus of F4/80 of mouse origin (1/400 dilution; Santa Cruz biotechnology) and a mouse monoclonal antibody targeting the O–4 antigen of *Salmonella* Typhimurium (1/1000, Santa Cruz biotechnology). After washing three times for 20 minutes, the samples were incubated with a goat anti-rabbit Alexa Fluor 488 (1/300; Life Technologies) targeting the F4/80 antibody, together with an Alexa Fluor 568 goat anti-mouse IgG1 (1/500; Life Technologies) targeting the *Salmonella* antibody. After an incubation of 1 hour, the sections were washed and incubated with DAPI. Finally the sections were mounted using ProLong Gold antifade mountant (Life Technologies). *Salmonella* containing cells were quantified blinded and visually by counting the number of F4/80 positive cells that co-localized with *Salmonella*. Per animal, three different sections were analyzed and per section, ten different areas were examined at a magnification of 400 X.

### *Salmonella* growth in cecal contents

The cecal contents of three seven-week-old DBA/2J mice was collected and ten times diluted in aqua dest. To remove larger particles, the suspension was filtered using a cell strainer (70 μm). *Salmonella* Typhimurium WT or Δ*scsA* bacteria were added to the cecal suspension (approximately 10^4^ CFU/ml) supplemented with HBSS-, 1 μM or 1 mM cortisol. Growth of *Salmonella* was examined at different time points (0, 4.5, 6.5, 20, 28 hours under microaerobic conditions at 37 °C) by plating ten-fold dilutions on BGA plates. This experiment was conducted in twofold.

### Statistical Analysis

All *in vitro* experiments were conducted at least in triplicate with 3 repeats per experiment, unless otherwise stated. All statistical analyses were performed using SPSS version 22 (SPSS Inc., Chicago, IL, USA). Normally distributed data were analyzed using unpaired Student’s t-test or one-way ANOVA to address the significance of difference between mean values with significance set at P ≤ 0.05. Bonferroni as post hoc test was used when equal variances were assessed. If equal variances were not assessed or if the data were not normally distributed, they were analyzed using the non parametric Kruskal-Wallis analysis, followed by a Bonferroni-corrected Mann-Whitney U test.

## Additional Information

**Accession Number**: Microarray data have been deposited in the Gene Expression Omnibus at NCBI with series accession numbers GSE30924 and GSE55430

**How to cite this article**: Verbrugghe, E. *et al.* Host Stress Drives *Salmonella* Recrudescence. *Sci. Rep.* **6**, 20849; doi: 10.1038/srep20849 (2016).

## Supplementary Material

Supplementary Information
