# Erratum: Host Stress Drives *Salmonella* Recrudescence

**DOI:** 10.1038/srep23877

**Published:** 2016-04-15

**Authors:** Elin Verbrugghe, Maarten Dhaenens, Bregje Leyman, Filip Boyen, Neil Shearer, Alexander Van Parys, Roel Haesendonck, Wim Bert, Herman Favoreel, Dieter Deforce, Arthur Thompson, Freddy Haesebrouck, Frank Pasmans

Scientific Reports 6: Article number: 20849; 10.1038/srep20849 published online: 02092016; updated: 04152016.

In the Supplementary Information file originally published with this Article, Figures S1-4, Tables S1, S2, S4 and S5 were omitted. This error has been corrected in the Supplementary Information that now accompanies the Article.

