# Supplementary material for: Host Stress Drives Salmonella Recrudescence
Source: Sci Rep. 2016 Feb 9;6:20849. doi: 10.1038/srep20849 (PMC4746619; doi:10.1038/srep20849)
Supplement: Supplementary Information [file srep20849-s1.pdf]

<u>Supplementary information</u>

Host Stress Drives *Salmonella* Recrudescence

Elin Verbrugghe[a,*], Maarten Dhaenens[b,¶], Bregje Leyman[a,¶,#], Filip Boyen[a], Neil Shearer[c], Alexander Van Parys[a,#], Roel Haesendonck[a], Wim Bert[d], Herman Favoreel[e], Dieter Deforce[b], Arthur Thompson[c], Freddy Haesebrouck[a], Frank Pasmans[a,*]

Supplementary Table S3:

ID	Common name	WT+C	t-test P-value	SCSA+C	t-test P-value
**Amino acid transport and metabolism**					
STM0002	thrA	0,81	0,05	1,03	0,08
STM0003	thrB	1,41	0,01	1,48	0,07
STM0006	yaaJ	1,65	0,03	1,41	0,01
STM0064	dapB	1,26	0,07	0,57	0,06
STM0066	carA	1,22	0,04	1,29	0,08
STM0110	leuD	0,52	0,06	7,26	0,00
STM0111	leuC	1,42	0,01	1,48	0,07
STM0112	leuB	2,19	0,01	0,88	0,09
STM0116	ilvI	0,97	0,09	3,80	0,00
STM0117	ilvH	0,15	0,03	0,63	0,05
STM0150	aroP	2,61	0,00	0,62	0,07
STM0165	speD	0,86	0,02	0,93	0,07
STM0166	speE	0,69	0,03	1,32	0,02
STM0234	ldcC	0,78	0,08	0,81	0,01
STM0235	yaeR	1,63	0,00	0,27	0,03
STM0248	yaeD	0,69	0,01	0,58	0,01
STM0316	pepD	1,05	0,10	0,62	0,06
STM0321	proB	1,75	0,02	0,60	0,04
STM0322	proA	0,54	0,02	0,62	0,02
STM0329		0,88	0,04	1,41	0,05
STM0330		1,24	0,08	0,16	0,00
STM0365	yahN	0,46	0,06	1,66	0,06
STM0386	proC	0,91	0,06	0,57	0,01
STM0388	aroL	0,84	0,04	0,74	0,02
STM0399	brnQ	4,89	0,03	0,54	0,04
STM0400	proY	0,61	0,01	4,03	0,04
STM0426	phnV	1,02	0,09	2,09	0,00
STM0428	phnT	0,72	0,03	0,53	0,01
STM0431	phnW	5,55	0,03	2,03	0,04
STM0458		0,30	0,01	0,35	0,03
STM0462	glnK	0,76	0,03	0,79	0,08
STM0506	tesA	1,45	0,03	1,56	0,06
STM0527	allC	0,81	0,03	0,83	0,05
STM0532	arcC	0,76	0,00	1,65	0,06
STM0568	pheP	0,66	0,04	0,91	0,08
STM0603	ybdL	4,03	0,03	0,63	0,06
STM0615	ybdR	0,48	0,01	2,61	0,04
STM0644	cobD				
STM0662	gltL	0,96	0,08	0,97	0,08
STM0663	gltK	1,50	0,02	1,19	0,04

STM0664	gltJ	1,61	0,01	2,05	0,02
STM0665	gltI	0,89	0,05	0,58	0,00
STM0680	asnB	1,05	0,08	0,96	0,07
STM0700	potE	0,24	0,00	1,12	0,09
STM0701	speF	0,51	0,04	1,46	0,06
STM0710	ybgH	1,09	0,09	1,22	0,07
STM0712	ybgJ	1,05	0,08	1,11	0,08
STM0713	ybgK	1,20	0,08	0,84	0,08
STM0760	aroG	0,70	0,01	0,67	0,01
STM0788	hutG	0,53	0,06	0,73	0,05
STM0791	hutH	0,64	0,05	1,00	0,10
STM0822	ybiB	1,10	0,01	0,64	0,00
STM0828	glnQ	0,78	0,00	0,64	0,00
STM0829	glnP	1,19	0,00	2,07	0,01
STM0830	glnH	1,21	0,04	0,69	0,03
STM0847	ybiK	1,07	0,06	0,69	0,00
STM0849	yliB	0,94	0,09	0,20	0,01
STM0850	yliC	0,49	0,03	1,46	0,07
STM0851	yliD	0,74	0,07	0,81	0,08
STM0877	potF	1,27	0,03	1,65	0,04
STM0878	potG	0,78	0,03	0,77	0,02
STM0879	potH	0,94	0,08	1,54	0,07
STM0880	potI	1,17	0,03	0,50	0,00
STM0888	artM	0,76	0,01	0,62	0,01
STM0889	artQ	1,01	0,10	1,51	0,00
STM0890	artI	0,87	0,02	0,55	0,00
STM0891	artP	0,85	0,03	0,63	0,02
STM0934	ltaA	3,39	0,03	0,14	0,01
STM0935	poxB	1,18	0,09	0,37	0,02
STM0969	ycaM	1,79	0,06	0,95	0,09
STM0978	aroA	0,78	0,02	1,13	0,02
STM0998	aspC	0,90	0,04	0,76	0,00
STM1002		1,03	0,10	1,37	0,07
STM1003		3,39	0,02	0,17	0,01
STM1057	pepN	0,96	0,09	1,24	0,08
STM1094	pipD	1,02	0,09	0,56	0,03
STM1104	hpaF			0,12	
STM1125	putP	2,58	0,02	0,63	0,03
STM1128		0,27	0,00	6,43	0,00
STM1135	ycdW	0,92	0,05	0,92	0,07
STM1136	ycdX	1,05	0,08	0,25	0,00
STM1160	solA	1,33	0,07	0,32	0,02
STM1198	pabC	0,78	0,01	0,48	0,00
STM1222	potD	0,22	0,02	0,21	0,00
STM1223	potC	4,91	0,02	0,20	0,04
STM1225	potB	0,76	0,00	1,54	0,06

STM1226	potA	0,36	0,03	1,01	0,10
STM1227	pepT	0,83	0,03	0,84	0,05
STM1255		0,85	0,02	1,46	0,07
STM1256		1,20	0,07	1,35	0,07
STM1257		3,79	0,02	0,18	0,01
STM1259		0,69	0,01	1,55	0,06
STM1269		1,51	0,01	1,56	0,07
STM1270	yeaS	1,14	0,02	0,81	0,05
STM1294	ansA	1,16	0,07	1,12	0,02
STM1297	selD	1,07	0,07	0,48	0,00
STM1299	gdhA	1,06	0,08	0,61	0,00
STM1303	astC	0,50	0,03	1,21	0,08
STM1304	astA	3,00	0,04	0,27	0,02
STM1306	astB	1,05	0,07	2,16	0,05
STM1307	astE	1,00	0,10	1,97	0,03
STM1347	aroH	1,30	0,04	1,52	0,02
STM1358	aroD	0,93	0,07	1,18	0,06
STM1359	ydiB	1,18	0,03	0,61	0,02
STM1373	sufS	0,39	0,03	1,54	0,05
STM1379	orf48	1,23	0,02	1,04	0,10
STM1435	gloA	3,04	0,03	0,13	0,01
STM1452	ydgR	1,70	0,02	1,22	0,09
STM1477	ydgI	0,76	0,02	0,74	0,01
STM1484		0,84	0,05	0,70	0,02
STM1491		0,93	0,07	0,81	0,05
STM1492		1,27	0,08	0,23	0,01
STM1494		2,26	0,00	2,36	0,04
STM1506	rspB	3,66	0,02	0,16	0,01
STM1512	dcp	0,67	0,03	0,67	0,00
STM1525	yneH	0,27	0,00	0,19	0,01
STM1542		2,73	0,04	1,36	0,06
STM1557		0,71	0,01	0,75	0,01
STM1584	ansP	0,58	0,06	1,00	0,10
STM1633		0,90	0,05	10,58	0,02
STM1634		1,02	0,09	1,45	0,04
STM1635		1,11	0,07	1,18	0,06
STM1636		1,04	0,09	1,18	0,08
STM1679	mppA	0,93	0,07	0,79	0,02
STM1680	ycjI	0,84	0,01	0,78	0,05
STM1692	sapA	1,20	0,02	1,04	0,09
STM1723	trpE	0,95	0,08	1,89	0,01
STM1724	trpD	0,21	0,01	1,27	0,06
STM1725	trpC	1,43	0,03	0,95	0,09
STM1726	trpB	1,54	0,09	1,91	0,06
STM1727	trpA	0,23	0,03	0,35	0,04
STM1742	oppF	1,14	0,08	0,90	0,09

STM1743	oppD	0,76	0,03	0,63	0,05
STM1744	oppC	1,05	0,06	0,94	0,07
STM1745	oppB	0,18	0,02	2,88	0,02
STM1746	oppA	1,06	0,07	0,63	0,00
STM1795		0,94	0,09	3,18	0,02
STM1803	dadA	0,84	0,02	0,77	0,04
STM1824	pabB	0,32	0,04	0,71	0,08
STM1826	sdaA	0,91	0,04	0,82	0,02
STM1879	ptrB	1,06	0,08	0,68	0,00
STM1937	tyrP	0,42	0,04	3,78	0,02
STM1951	yecC	0,95	0,09	1,04	0,08
STM1952	yecS	0,87	0,03	0,62	0,03
STM1953	yedO	0,85	0,01	0,74	0,04
STM1954	fliY	2,09	0,03	0,86	0,09
STM2039	pudB	1,03	0,09	1,53	0,07
STM2055	pduU	0,69	0,08	1,52	0,01
STM2056	pduV	0,67	0,02	0,93	0,03
STM2068	yeeF	0,66	0,08	4,31	0,03
STM2071	hisG	1,03	0,08	1,40	0,02
STM2072	hisD	0,86	0,04	0,64	0,01
STM2073	hisC	1,16	0,01	0,87	0,02
STM2074	hisB	0,76	0,06	0,33	0,02
STM2075	hisH	1,38	0,01	0,52	0,01
STM2077	hisF	0,86	0,04	0,56	0,00
STM2078	hisI	0,82	0,06	1,59	0,03
STM2114	wcaB	1,08	0,05	0,90	0,09
STM2162	yehW	0,96	0,08	1,09	0,08
STM2163	yehX	1,38	0,06	2,13	0,03
STM2164	yehY	0,65	0,03	0,62	0,01
STM2186		1,00	0,10	1,40	0,07
STM2196		0,97	0,10	0,32	0,03
STM2197		0,65	0,05	1,72	0,07
STM2200	lysP	1,19	0,07	1,16	0,03
STM2216	yejA	2,64	0,07	0,92	0,07
STM2285	glpB	2,31	0,04	0,17	0,01
STM2331	yfbQ	1,08	0,01	0,89	0,05
STM2351	hisP	1,12	0,02	1,26	0,08
STM2352	hisM	9,39	0,00	0,67	0,07
STM2353	hisQ	0,94	0,09	0,63	0,07
STM2354	hisJ	0,88	0,03	0,55	0,01
STM2355	argT	0,47	0,05	0,74	0,07
STM2357		0,89	0,06	1,19	0,04
STM2358		2,06	0,06	0,68	0,06
STM2359		1,70	0,07	0,41	0,01
STM2360		0,54	0,00	0,53	0,01
STM2369	usg	0,94	0,08	0,76	0,03

STM2370	pdxB	0,99	0,10	0,63	0,00
STM2384	aroC	1,01	0,10	0,65	0,06
STM2402	yfdZ	0,88	0,04	0,54	0,00
STM2429	cysZ	0,97	0,08	1,10	0,07
STM2430	cysK	1,28	0,02	1,46	0,08
STM2440	cysM	1,01	0,10	1,35	0,07
STM2456	eutL	5,65	0,04	0,40	0,01
STM2457	eutC	0,70	0,02	0,66	0,00
STM2458	eutB	4,27	0,02	1,56	0,03
STM2459	eutA	0,98	0,09	1,23	0,03
STM2460	eutH	2,36	0,01	3,65	0,02
STM2462	eutJ	0,83	0,03	1,54	0,04
STM2467	eutT	0,98	0,10	0,91	0,07
STM2468	eutQ	0,80	0,04	0,76	0,01
STM2469	eutP	1,07	0,08	1,37	0,01
STM2470	eutS	2,06	0,00	0,76	0,01
STM2479	aegA	1,20	0,07	2,22	0,03
STM2483	dapE	1,37	0,06	0,83	0,07
STM2489	dapA	0,85	0,08	1,03	0,10
STM2490	gcvR	0,87	0,07	0,85	0,08
STM2536	pepB	0,30	0,04	0,92	0,08
STM2543	nifS	1,35	0,01	0,91	0,02
STM2555	glyA	0,52	0,02	0,69	0,02
STM2558	cadB	0,51	0,02	0,44	0,00
STM2559	cadA	0,90	0,08	0,97	0,10
STM2560	yjdL	0,19	0,03	0,91	0,08
STM2561	glnB	1,26	0,02	1,05	0,07
STM2569	yfhB	0,91	0,07	1,39	0,02
STM2645	yfiK	0,66	0,07	1,51	0,01
STM2667	pheA	0,78	0,03	0,95	0,08
STM2669	tyrA	1,12	0,03	0,43	0,00
STM2670	aroF	0,77	0,03	0,51	0,02
STM2792	gabT	0,93	0,09	0,45	0,04
STM2793	gabP	1,09	0,06	2,98	0,04
STM2809	proV	3,55	0,02	0,15	0,01
STM2810	proW	0,92	0,06	1,03	0,09
STM2811	proX	0,99	0,10	0,58	0,02
STM2935	cysD	1,14	0,06	0,42	0,04
STM2946	cysH	1,26	0,00	0,64	0,01
STM2970	sdaC	1,05	0,01	0,63	0,01
STM2971	sdaB	0,94	0,05	0,89	0,02
STM2984	csdA	3,30	0,00	7,34	0,00
STM2992	argA	0,90	0,07	0,34	0,01
STM3013	lysA	0,84	0,05	0,62	0,02
STM3022		0,53	0,05	6,97	0,02
STM3053	gcvP	0,85	0,02	0,69	0,00

STM3054	gcvH	0,34	0,04	0,60	0,07
STM3055	gcvT	1,08	0,07	0,40	0,01
STM3058	pepP	1,21	0,02	0,66	0,03
STM3062	serA	1,03	0,08	0,62	0,01
STM3078	speB	0,77	0,00	0,77	0,02
STM3082		0,39	0,04	1,56	0,06
STM3106	ansB	0,19	0,02	1,25	0,08
STM3114	speC	1,97	0,03	0,89	0,06
STM3117		1,26	0,08	0,15	0,01
STM3126		0,64	0,05	4,81	0,02
STM3128		1,54	0,02	1,59	0,07
STM3139	gsp	0,94	0,07	0,85	0,06
STM3161	metC	0,68	0,02	1,08	0,07
STM3188	ygiC	0,82	0,06	0,85	0,08
STM3218	oat	1,30	0,05	0,30	0,01
STM3225	ygjU	0,89	0,09	0,99	0,10
STM3239	yhaO	0,64	0,04	3,42	0,03
STM3240	tdcG	0,44	0,05	1,27	0,08
STM3243	tdcC	0,33	0,01	1,26	0,08
STM3244	tdcB	1,00	0,10	5,31	0,01
STM3261		0,75	0,00	1,61	0,06
STM3279	mtr	0,79	0,02	0,75	0,02
STM3290	argG	0,85	0,03	0,59	0,02
STM3331	gltD	1,44	0,00	1,42	0,07
STM3339	nanA	2,03	0,00	2,01	0,04
STM3360	argR	2,79	0,03	0,13	0,01
STM3401	aroE	0,84	0,03	0,60	0,00
STM3468	argD	1,26	0,01	0,42	0,01
STM3469	pabA	1,36	0,06	0,91	0,06
STM3486	aroB	0,47	0,01	1,07	0,01
STM3487	aroK	1,12	0,06	0,61	0,02
STM3532		0,69	0,02	0,80	0,07
STM3539	asd	0,78	0,04	0,71	0,01
STM3551	ggt	1,14	0,08	0,59	0,03
STM3560	livF	0,67	0,01	1,02	0,09
STM3561	livG	0,80	0,02	1,65	0,03
STM3562	livM	1,36	0,05	1,83	0,06
STM3563	livH	0,97	0,09	0,64	0,01
STM3564	livK	0,52	0,04	6,24	0,01
STM3567	livJ	0,58	0,04	10,80	0,00
STM3592	yhiP	0,75	0,02	0,98	0,10
STM3594	prlC	1,30	0,04	1,41	0,07
STM3598				0,27	0,01
STM3625	yhjV	0,84	0,02	1,80	0,03
STM3627	dppD	0,89	0,05	0,68	0,01
STM3628	dppC	0,76	0,01	1,08	0,07

STM3629	dppB	1,35	0,02	1,15	0,03
STM3630	dppA	1,31	0,03	1,44	0,07
STM3665	avtA	1,27	0,01	0,79	0,00
STM3683	selA	1,02	0,09	0,70	0,00
STM3699	cysE	1,11	0,06	0,60	0,01
STM3708	tdh	1,17	0,02	0,69	0,00
STM3746	gltS	0,11	0,01	0,75	0,08
STM3768		0,98	0,10	1,21	0,08
STM3795	ilvN	1,01	0,08	0,60	0,03
STM3796	ilvB	0,86	0,03	0,87	0,09
STM3802	dsdA	1,83	0,05	0,91	0,09
STM3859		1,00	0,10	1,03	0,09
STM3877	asnA	1,11	0,08	1,19	0,09
STM3901	ilvG	0,85	0,02	0,90	0,07
STM3903	ilvE	0,63	0,00	1,87	0,06
STM3904	ilvD	1,08	0,04	0,58	0,02
STM3905	ilvA	0,95	0,04	0,82	0,02
STM3909	ilvC	1,23	0,01	0,41	0,00
STM3930	yifK	0,75	0,03	0,72	0,01
STM3947	dapF	0,73	0,00	0,49	0,01
STM3959	rhtC	0,70	0,02	0,47	0,01
STM3960	rthB	0,79	0,05	0,72	0,01
STM3965	metE	0,71	0,01	0,87	0,04
STM3984	pepQ	3,36	0,03	0,17	0,01
STM4007	glnA	0,95	0,06	0,55	0,00
STM4042		1,26	0,04	5,93	0,02
STM4099	metJ	0,74	0,00	0,65	0,02
STM4100	metB	0,41	0,05	0,67	0,07
STM4105	metF	1,52	0,04	4,99	0,03
STM4120	argE	0,93	0,02	0,64	0,02
STM4121	argC	1,36	0,03	1,33	0,08
STM4122	argB	1,06	0,05	0,47	0,01
STM4182	metA	3,81	0,01	1,95	0,02
STM4188	metH	0,20	0,02	1,15	0,08
STM4190	pepE	0,82	0,01	0,92	0,08
STM4220	lysC	0,87	0,00	0,73	0,03
STM4248	tyrB	0,95	0,07	0,77	0,02
STM4296	adi			0,62	
STM4326	aspA	1,16	0,08	0,70	0,07
STM4328	yjeH	1,04	0,10	1,10	0,09
STM4333	yjeK	0,85	0,06	0,91	0,09
STM4345	yjeM	0,85	0,03	0,99	0,10
STM4351		0,57	0,04	0,46	0,01
STM4376	yjfC	0,62	0,04	1,69	0,07
STM4398	cycA	0,12	0,01	1,22	0,07
STM4431		1,06	0,05	2,22	0,05

STM4432		0,34	0,00	1,63	0,04
STM4446		0,39	0,04	2,28	0,04
STM4463		0,97	0,07	0,77	0,02
STM4465		0,72	0,01	0,66	0,00
STM4466		0,96	0,03	0,48	0,02
STM4467					
STM4469	argI	1,14	0,00	0,53	0,01
STM4477	pepA	0,83	0,00	0,80	0,01
STM4484	idnD	0,97	0,08	0,52	0,02
STM4578	serB	0,45	0,04	0,60	0,03
**Carbohydrate transport and metabolism**					
STM0007	talB	1,41	0,01	0,84	0,03
STM0018		0,12	0,03	0,64	0,00
STM0041		0,57	0,04	15,30	0,02
STM0042		0,68	0,01	4,78	0,03
STM0060	citE2	3,37	0,02	0,19	0,01
STM0079	yaaU	0,24	0,05	0,42	0,05
STM0101	araD	0,65	0,05	2,89	0,04
STM0149		0,78	0,02	0,80	0,02
STM0169	gcd	0,90	0,05	0,76	0,03
STM0178	yadI	0,44	0,02	0,69	0,08
STM0212		1,08	0,09	0,97	0,06
STM0257		1,97	0,00	5,25	0,01
STM0303	ybeJ	1,42	0,07	0,34	0,00
STM0310	ghmA	0,71	0,00	0,54	0,00
STM0356		0,01	0,10	4,84	0,04
STM0368	prpB	0,88	0,05	0,72	0,01
STM0382		0,84	0,01	0,99	0,09
STM0394	araJ	0,24	0,03	0,59	0,02
STM0401	malZ	0,72	0,08	3,40	0,01
STM0436	yajR	0,82	0,04	0,77	0,00
STM0444	ampG	0,63	0,00	7,62	0,02
STM0491	gsk	1,19	0,06	1,12	0,09
STM0493	fsr	0,72	0,01	0,78	0,01
STM0518	gip	0,73	0,02	0,79	0,02
STM0520		0,97	0,09	0,62	0,02
STM0525	glxK	2,16	0,00	2,38	0,04
STM0574		0,99	0,09	0,51	0,02
STM0575		1,09	0,05	1,48	0,07
STM0576		0,78	0,01	1,65	0,03
STM0577		1,01	0,10	1,14	0,09
STM0593	ybdA	0,99	0,10	1,31	0,01
STM0622	citE	0,85	0,03	2,59	0,05
STM0643	cobC	1,45	0,01	3,77	0,00
STM0649		0,24	0,00	1,64	0,06

STM0650		1,01	0,10	1,17	0,09
STM0681	nagD	1,44	0,02	1,17	0,01
STM0683	nagA	0,91	0,04	1,14	0,04
STM0684	nagB	1,20	0,02	1,79	0,03
STM0685	nagE	0,18	0,02	1,30	0,02
STM0689	citA	0,84	0,02	2,09	0,05
STM0698	pgm	0,48	0,04	0,60	0,04
STM0722		0,75	0,02	0,84	0,02
STM0723		0,69	0,04	0,97	0,10
STM0772	gpmA	1,10	0,06	0,41	0,00
STM0773	galM	1,06	0,07	0,46	0,04
STM0785	ybhE	1,50	0,02	0,71	0,00
STM0786	ybhC	1,46	0,02	1,35	0,00
STM0854		0,59	0,04	1,86	0,07
STM0860		1,38	0,05	2,75	0,06
STM0861	yliI	3,56	0,02	0,27	0,02
STM0866	mdfA	1,61	0,06	0,97	0,10
STM0868		0,94	0,08	1,32	0,06
STM0885		0,40	0,02	3,91	0,02
STM0928	nanH	0,92	0,07	1,30	0,08
STM0968	ycaD	1,06	0,07	1,25	0,08
STM1076	mgsA	1,02	0,10	0,79	0,08
STM1106	hpaI	1,15	0,06	1,25	0,05
STM1107	hpaX	0,94	0,07	1,04	0,09
STM1129		1,04	0,09	2,35	0,03
STM1132		0,74	0,01	1,50	0,04
STM1154	yceE	0,08	0,00	2,38	0,00
STM1166	yceL	0,17	0,02	0,91	0,08
STM1203	ptsG	0,81	0,02	0,55	0,00
STM1209	nagZ	0,91	0,02	0,76	0,01
STM1252		0,88	0,02	1,13	0,06
STM1289	yeaD	0,98	0,09	0,66	0,00
STM1290	gapA	1,35	0,04	1,44	0,07
STM1313	celB	1,03	0,09	1,87	0,01
STM1314	celC	0,25	0,03	1,41	0,07
STM1316	celF	1,06	0,07	2,56	0,04
STM1326	pfkB	0,78	0,01	0,75	0,00
STM1349	pps	1,11	0,04	0,61	0,00
STM1360	ydiN	0,84	0,03	2,68	0,05
STM1378	pykF	0,89	0,03	0,76	0,01
STM1428	ydhC	1,11	0,09	0,95	0,09
STM1467	manA	1,67	0,05	0,63	0,05
STM1486	ynfM	0,93	0,06	1,07	0,06
STM1507	ydfJ	0,60	0,03	1,63	0,07
STM1508	ydfI	0,78	0,01	1,53	0,06
STM1516	ydeE	13,96	0,00	2,66	0,04

STM1517	ydeD	2,30	0,03	0,45	0,00
STM1522	ydeA				
STM1543		1,08	0,05	1,71	0,06
STM1545		0,33	0,03	5,57	0,02
STM1558		0,23	0,04	0,64	0,00
STM1559		0,99	0,10	0,57	0,01
STM1560		7,27	0,00	0,82	0,08
STM1571	yddG	0,57	0,00	6,06	0,00
STM1574	smvA	1,14	0,06	1,11	0,06
STM1612		0,98	0,07	0,80	0,03
STM1613		1,12	0,05	1,96	0,05
STM1614		0,85	0,04	0,57	0,01
STM1616		0,93	0,05	0,63	0,02
STM1617		1,73	0,05	0,69	0,07
STM1708	yciM	0,85	0,05	0,87	0,09
STM1796	treA	0,42	0,04	0,94	0,08
STM1830	manX	0,96	0,07	0,54	0,01
STM1831	manY	0,84	0,04	0,60	0,00
STM1832	manZ	0,84	0,05	1,27	0,08
STM1843		1,04	0,07	0,69	0,07
STM1862	pagO	0,68	0,08	0,43	0,04
STM1884	eda	1,03	0,09	0,81	0,06
STM1886	zwf	1,03	0,10	0,70	0,06
STM1888	pykA	1,32	0,03	0,66	0,03
STM1928	otsA	1,49	0,03	1,38	0,07
STM1929	otsB	0,94	0,08	0,88	0,06
STM1933		0,83	0,04	0,64	0,06
STM1963	amyA	1,29	0,04	0,85	0,08
STM1990	yedA	1,09	0,07	1,47	0,04
STM2037	pduF	1,15	0,06	0,79	0,03
STM2081	gnd	0,86	0,06	0,72	0,06
STM2083	rfbK	2,58	0,02	0,79	0,06
STM2129	yegB	1,33	0,08	0,46	0,03
STM2141	fbaB	0,88	0,05	0,41	0,00
STM2142	yegT	1,04	0,08	2,27	0,05
STM2144	yegV	0,41	0,01	0,77	0,01
STM2166	bglX	2,72	0,04	1,56	0,07
STM2179		0,94	0,06	0,93	0,07
STM2188	mglC	1,09	0,09	1,42	0,07
STM2189	mglA	0,74	0,00	1,76	0,06
STM2190	mglB	0,85	0,03	1,09	0,08
STM2198		0,71	0,02	1,28	0,07
STM2205	fruK	0,69	0,02	0,66	0,05
STM2206	fruF	0,83	0,04	0,58	0,00
STM2207	setB	0,32	0,01	2,24	0,02
STM2221	bcr	0,47	0,06	0,76	0,08

STM2274		0,60	0,04	7,18	0,02
STM2280		3,48	0,02	0,18	0,01
STM2283	glpT	1,16	0,09	1,09	0,09
STM2289		1,12	0,03	3,74	0,02
STM2290	yfaV	0,38	0,02	2,85	0,00
STM2300		1,12	0,01	0,99	0,10
STM2302		1,19	0,04	0,76	0,03
STM2303		1,19	0,05	1,29	0,08
STM2340		0,37	0,00	2,06	0,04
STM2341		0,93	0,08	1,37	0,01
STM2343		1,69	0,06	0,65	0,02
STM2344		2,25	0,00	5,91	0,01
STM2372		1,03	0,10	0,56	0,00
STM2399	pgtP	3,04	0,03	0,46	0,04
STM2403	glk	0,93	0,04	0,79	0,01
STM2405		1,07	0,08	1,27	0,09
STM2421	xapB	1,14	0,05	0,61	0,01
STM2431	ptsH	1,58	0,00	0,69	0,03
STM2432	ptsI	1,14	0,02	0,64	0,01
STM2433	crr	1,51	0,03	1,25	0,08
STM2473	talA	1,07	0,08	0,35	0,04
STM2474	tktB	1,07	0,06	0,83	0,02
STM2492		2,16	0,07	0,28	0,02
STM2546	suhB	1,18	0,08	0,69	0,08
STM2554	hcaT	0,92	0,08	1,54	0,06
STM2570		0,81	0,05	1,40	0,07
STM2574		1,18	0,02	0,58	0,01
STM2654	kgtP	1,78	0,06	1,53	0,06
STM2668		1,33	0,01	0,57	0,04
STM2750		0,67	0,05	1,77	0,07
STM2751		0,79	0,01	1,39	0,07
STM2752		0,51	0,02	1,24	0,08
STM2755		0,74	0,00	1,50	0,06
STM2757		3,69	0,02	0,14	0,01
STM2758		0,66	0,05	1,60	0,07
STM2773	iroB	0,96	0,08	1,20	0,05
STM2815	emrB	0,83	0,07	0,74	0,07
STM2832	srlA	1,01	0,10	1,49	0,04
STM2833	srlE	0,70	0,02	0,88	0,09
STM2834	slrB	0,89	0,05	0,98	0,10
STM2911		1,09	0,08	1,19	0,08
STM2913		0,65	0,05	2,34	0,05
STM2915	ygbM	0,77	0,01	1,52	0,06
STM2916	ygbL	0,85	0,03	4,90	0,03
STM2952	eno	1,18	0,04	0,34	0,00
STM2959		1,24	0,02	0,53	0,05

STM2962	gudT	1,87	0,05	1,88	0,04
STM2974	fucA	1,00	0,10	0,50	0,01
STM2976	fucI	0,72	0,02	1,51	0,06
STM2977	fucK	0,80	0,04	0,23	0,00
STM2978	fucU	2,08	0,00	2,29	0,04
STM3009	ygeD	1,06	0,05	0,33	0,03
STM3016	araE	1,02	0,09	2,60	0,04
STM3018	kduI	0,96	0,05	0,65	0,02
STM3051	bglA	0,79	0,02	0,97	0,04
STM3068	fba	0,92	0,06	0,61	0,00
STM3069	pgk	1,01	0,09	1,40	0,08
STM3076	tktA	1,13	0,05	0,76	0,03
STM3083		1,05	0,09	1,15	0,06
STM3091	galP	1,13	0,08	2,51	0,04
STM3113	nupG	9,18	0,01	7,38	0,00
STM3120		1,23	0,06	1,19	0,07
STM3132		0,41	0,05	0,36	0,00
STM3134		0,42	0,00	1,08	0,05
STM3136		0,33	0,04	1,60	0,05
STM3137		0,17	0,00	2,02	0,02
STM3169		0,88	0,06	1,22	0,02
STM3170		0,77	0,02	0,80	0,03
STM3171	ygiK	0,75	0,00	1,62	0,06
STM3247	garK	0,88	0,05	0,89	0,06
STM3249	garL	0,23	0,01	1,40	0,01
STM3250	garD	0,57	0,06	1,52	0,05
STM3251		0,77	0,01	0,80	0,06
STM3253		0,70	0,00	3,50	0,02
STM3254		0,60	0,04	9,99	0,02
STM3255		0,50	0,03	1,27	0,08
STM3256		0,79	0,08	0,47	0,04
STM3257		3,61	0,02	0,17	0,01
STM3258		0,54	0,03	1,77	0,07
STM3259		0,97	0,09	1,32	0,01
STM3260		1,10	0,08	1,30	0,07
STM3266	yraO	1,79	0,01	1,44	0,06
STM3294	mrsA	1,14	0,07	1,00	0,10
STM3322	ptsN	1,18	0,03	0,83	0,01
STM3324	ptsO	0,81	0,07	1,05	0,10
STM3335	yhcH	0,92	0,05	0,57	0,00
STM3337	nanE	1,21	0,08	1,04	0,10
STM3338	nanT	0,11	0,01	0,98	0,10
STM3473	yhfC	0,92	0,09	1,83	0,01
STM3483	rpe	1,01	0,09	0,75	0,01
STM3512	gntT	0,42	0,04	1,17	0,09
STM3534	glgP	3,08	0,03	0,12	0,01

STM3535	glgA	0,81	0,04	1,29	0,08
STM3537	glgX	0,67	0,06	0,95	0,10
STM3538	glgB	0,75	0,04	0,74	0,07
STM3541	gntU	0,49	0,02	1,23	0,08
STM3542	gntK	3,42	0,02	0,17	0,01
STM3547		1,02	0,09	1,36	0,01
STM3554	ugpC	0,96	0,10	0,66	0,04
STM3555	ugpE	0,94	0,09	0,39	0,01
STM3556	ugpA	0,93	0,08	1,26	0,01
STM3557	ugpB	2,17	0,03	2,00	0,05
STM3581	yhhS	0,66	0,04	17,13	0,00
STM3600		0,74	0,04	0,56	0,02
STM3603	treF	0,90	0,06	0,66	0,01
STM3609	yhjE	1,06	0,07	0,82	0,04
STM3612	kdgK	0,89	0,07	0,68	0,07
STM3617	bcsC	0,24	0,02	5,98	0,01
STM3660	xylB	0,82	0,02	0,52	0,04
STM3661	xylA	1,07	0,07	1,18	0,08
STM3664	malS	1,04	0,09	1,42	0,04
STM3669	yiaL	0,51	0,04	3,12	0,02
STM3671	yiaM	3,47	0,02	0,14	0,01
STM3672	yiaN	1,05	0,07	1,38	0,06
STM3673	yiaO	0,95	0,07	2,75	0,05
STM3674	lyxK	1,19	0,08	0,82	0,08
STM3676	sgbU	1,09	0,07	1,41	0,04
STM3685	mtlA	1,12	0,03	0,74	0,01
STM3686	mtlD	1,02	0,09	0,62	0,00
STM3698		0,80	0,01	0,84	0,08
STM3704	pmgI	1,40	0,01	1,32	0,07
STM3750	yicJ	1,00	0,10	1,16	0,06
STM3765	yicL	2,02	0,04	1,17	0,05
STM3769		0,57	0,03	1,49	0,08
STM3770		3,38	0,02	0,13	0,01
STM3771					
STM3772		0,27		4,21	0,05
STM3775		4,39	0,03	0,38	0,03
STM3776	yicM	1,15	0,03	2,27	0,05
STM3779		0,72	0,00	1,48	0,07
STM3780		0,92	0,08	1,44	0,07
STM3781		0,65	0,06	1,31	0,08
STM3782		0,85	0,09	0,70	0,07
STM3783		0,16	0,03	10,40	0,00
STM3784		1,92	0,05	0,30	0,02
STM3787	uhpT	0,78	0,01	0,54	0,00
STM3788	uhpC	1,00	0,10	1,21	0,04
STM3792		0,48	0,04	0,18	0,01

STM3793		1,36	0,05	2,28	0,03
STM3798	emrD	1,10	0,08	0,55	0,00
STM3801	dsdX	1,03	0,10	0,32	0,01
STM3825	torT	0,72	0,03	1,31	0,07
STM3827	dgoT	0,90	0,07	1,32	0,01
STM3829	dgoK	0,77	0,05	0,74	0,04
STM3832		0,80	0,02	0,80	0,02
STM3847	yidY	0,13	0,02	1,11	0,09
STM3858		1,11	0,01	1,33	0,02
STM3881	rbsD	0,77	0,01	0,79	0,08
STM3882	rbsA	1,02	0,09	1,54	0,01
STM3883	rbsC	0,66	0,02	4,29	0,01
STM3884	rbsB	0,95	0,08	0,65	0,00
STM3885	rbsK	1,99	0,06	0,89	0,07
STM3887	yieO	1,26	0,08	1,41	0,08
STM3963	yigM	0,70	0,00	1,11	0,08
STM4017	yihO	1,11	0,08	1,20	0,08
STM4018	yihP	0,77	0,01	1,08	0,07
STM4019	yihQ	0,78	0,01	1,60	0,06
STM4020	yihR	0,93	0,09	0,27	0,01
STM4021	yihS	1,46	0,06	6,16	0,02
STM4022	yihT	1,10	0,03	0,57	0,02
STM4024	yihV	0,86	0,04	0,82	0,01
STM4045	rhaD	3,64	0,02	0,57	0,05
STM4046	rhaA	1,87	0,03	1,42	0,04
STM4047	rhaB	0,99	0,09	2,53	0,01
STM4050	rhaT	0,60	0,01	0,72	0,02
STM4052		0,11	0,00	2,32	0,00
STM4053		0,60	0,04	5,48	0,02
STM4054		0,82	0,05	0,66	0,02
STM4062	pfkA	0,86	0,04	0,65	0,01
STM4065		0,97	0,07	0,55	0,02
STM4066		1,12	0,05	2,45	0,04
STM4072	ydeV	0,27	0,01	0,31	0,02
STM4074	ego	2,08	0,00	4,74	0,01
STM4076	ydeZ	1,03	0,08	2,10	0,05
STM4077	yneA	0,76	0,01	0,74	0,06
STM4078	yneB	0,65	0,01	1,21	0,01
STM4080		0,53	0,00	0,75	0,02
STM4081	tpiA	1,19	0,04	0,62	0,06
STM4085	glpX	0,85	0,02	1,00	0,10
STM4087	glpF	0,89	0,06	0,67	0,01
STM4109	talC	0,25	0,01	1,40	0,04
STM4110	ptsA	1,23	0,06	1,09	0,09
STM4112	frwC	2,65	0,00	1,13	0,07
STM4113	frwB			0,32	0,02

STM4116	frwD	0,69	0,05	0,78	0,01
STM4221	pgi	1,06	0,08	0,65	0,01
STM4227	malG	1,03	0,09	0,44	0,01
STM4228	malF	0,98	0,09	0,67	0,03
STM4229	malE	0,16	0,03	2,30	0,05
STM4230	malK	1,24	0,04	1,05	0,08
STM4290	proP	0,99	0,09	0,69	0,03
STM4298	melA	5,79	0,04	1,20	0,04
STM4299	melB	0,80	0,02	1,36	0,07
STM4384	sgaB	2,60	0,00	2,13	0,04
STM4385	ptxA	0,95	0,07	0,44	0,00
STM4386	sgaH	1,01	0,10	1,17	0,02
STM4387	sgaU	1,03	0,09	1,50	0,07
STM4388	sgaE	0,91	0,09	1,73	0,05
STM4395	yifZ	1,31	0,06	1,78	0,04
STM4400	ytfF	1,69	0,04	1,37	0,07
STM4412		0,59	0,07	1,00	0,10
STM4415	fbp	3,46	0,03	0,12	0,01
STM4418		2,26	0,00	2,37	0,04
STM4419		1,05	0,08	1,20	0,01
STM4420		0,97	0,10	0,99	0,10
STM4424		0,77	0,03	0,72	0,01
STM4427		0,52	0,03	1,20	0,02
STM4428		2,80	0,01	0,67	0,01
STM4430		0,70	0,03	0,60	0,02
STM4434		1,01	0,09	0,73	0,05
STM4435		1,14	0,04	2,15	0,05
STM4436		0,79	0,01	1,43	0,04
STM4448		1,52	0,05	0,70	0,05
STM4453	treC	0,91	0,06	2,97	0,03
STM4468	yjgK	0,80	0,02	0,75	0,01
STM4482	idnT	0,56	0,07	0,70	0,07
STM4485	idnK	1,17	0,04	2,08	0,05
STM4515	yjiJ	0,53	0,02	1,15	0,09
STM4517	yjiO	0,89	0,09	1,44	0,02
STM4535		1,10	0,08	0,96	0,09
STM4536		1,11	0,07	1,14	0,07
STM4537		1,02	0,09	0,73	0,04
STM4538		1,02	0,08	0,43	0,01
STM4569	deoB	1,25	0,02	0,65	0,02
STM4585	gpmB	1,10	0,09	0,86	0,08

Cell motility and secretion

STM0021	bcfA	1,00	0,10	0,14	0,00
STM0022	bcfB	1,02	0,09	1,67	0,06
STM0023	bcfC	0,80	0,01	7,46	0,02

STM0024	bcfD	0,39	0,00	2,02	0,04
STM0025	bcfE	0,72	0,01	1,54	0,04
STM0026	bcfF	0,23	0,03	0,18	0,00
STM0027	bcfG	1,08	0,05	0,59	0,01
STM0142	hofC	2,07	0,00	2,59	0,03
STM0143	hofB	0,77	0,01	1,57	0,04
STM0144	ppdD	1,05	0,08	0,69	0,01
STM0144	ppdD				
STM0174	stiH	0,43	0,04	0,35	0,02
STM0175	stiC				
STM0176	stiB	1,07	0,05	0,49	0,02
STM0177	stiA	1,06	0,07	1,03	0,10
STM0195	stfA	2,33	0,00	2,68	0,03
STM0196	stfC	0,74	0,01	1,24	0,07
STM0197	stfD	0,98	0,09	1,21	0,03
STM0198	stfE	0,84	0,04	0,75	0,01
STM0199	stfF	0,72	0,03	0,56	0,02
STM0200	stfG	0,90	0,07	0,80	0,03
STM0300	safB	1,98	0,01	2,37	0,04
STM0301	safC	1,02	0,10	1,58	0,02
STM0336	stbE	0,88	0,08	0,74	0,05
STM0338	stbC	0,52	0,02	6,27	0,00
STM0339	stbB	1,04	0,07	8,45	0,02
STM0340	stbA	0,64	0,02	0,90	0,09
STM0543	fimA	1,05	0,02	0,66	0,01
STM0544	fimI	8,45	0,01	1,14	0,09
STM0545	fimC			0,57	0,05
STM0546	fimD	0,93	0,06	3,41	0,01
STM0548	fimF	0,96	0,08	0,70	0,02
STM1171	flgN	0,10	0,00	4,35	0,01
STM1173	flgA	2,46	0,04	0,65	0,03
STM1174	flgB	20,72		0,53	0,02
STM1175	flgC	1,13	0,04	0,68	0,03
STM1176	flgD	0,76	0,01	1,62	0,03
STM1177	flgE	0,86	0,02	3,33	0,01
STM1178	flgF	1,13	0,04	0,62	0,02
STM1179	flgG	0,88	0,04	2,55	0,05
STM1180	flgH	4,02	0,02	1,79	0,06
STM1181	flgI	1,10	0,04	0,66	0,02
STM1183	flgK	0,79	0,02	0,61	0,00
STM1184	flgL	0,94	0,10	0,58	0,06
STM1394	ssaC	1,31	0,06	1,90	0,06
STM1415	ssaN	0,51	0,00	0,55	0,02
STM1626	trg	1,08	0,07	1,44	0,04
STM1657		1,57	0,01	3,72	0,04
STM1915	cheZ	0,63	0,01	1,72	0,06

STM1917	cheB	0,69	0,01	0,45	0,03
STM1918	cheR	0,66	0,02	0,80	0,00
STM1920	cheW	0,92	0,10	0,42	0,03
STM1921	cheA	1,01	0,10	0,54	0,01
STM1922	motB	3,76	0,02	0,17	0,01
STM1923	motA	1,04	0,09	1,59	0,06
STM1959	fliC	0,74	0,01	0,72	0,03
STM1960	fliD	0,79	0,04	0,62	0,07
STM1968	fliE			2,37	0,00
STM1969	fliF			0,39	0,00
STM1970	fliG	0,84	0,03	7,18	0,02
STM1971	fliH	0,54	0,00	0,49	0,01
STM1972	fliI	1,00	0,10	2,06	0,00
STM1973	fliJ	0,96	0,09	1,43	0,04
STM1974	fliK	1,04	0,08	0,69	0,03
STM1975	fliL	0,93	0,04	1,02	0,09
STM1976	fliM	0,30	0,00	3,29	0,01
STM1977	fliN	2,05	0,05	1,68	0,03
STM1978	fliO	1,74	0,05	0,34	0,02
STM1979	fliP	0,50	0,04	3,09	0,03
STM1980	fliQ	0,50	0,02	0,97	0,09
STM1981	fliR	1,10	0,08	1,17	0,09
STM2150	stcC	0,94	0,09	1,20	0,08
STM2151	stcB	1,05	0,05	0,45	0,02
STM2152	stcA	0,32	0,00	2,11	0,03
STM2771	fljB	0,90	0,00	0,81	0,04
STM2887	spaS	0,87	0,07	1,63	0,04
STM2894	invC	0,94	0,05	2,27	0,05
STM2997	ppdC	0,73	0,00	2,52	0,02
STM3000	ppdA	1,93	0,05	0,58	0,06
STM3027	stdC	0,62	0,00	1,05	0,09
STM3028	stdB	0,92	0,06	1,07	0,05
STM3029	stdA	0,69	0,01	2,61	0,04
STM3138		0,72	0,07	0,84	0,08
STM3152		1,07	0,09	1,29	0,08
STM3216		0,64	0,05	4,22	0,03
STM3442	hopD	0,61	0,07	0,37	0,04
STM3491	yrfC	1,11	0,09	0,54	0,05
STM3636	lpfE	1,05	0,07	0,38	0,00
STM3637	lpfD	1,82	0,05	0,42	0,01
STM3638	lpfC	0,87	0,03	2,30	0,05
STM3639	lpfB	0,99	0,10	1,15	0,07
STM3640	lpfA	0,79	0,03	4,12	0,03
STM4533	tsr	0,33	0,02	2,48	0,03
STM4572	stjB	0,26	0,00	1,39	0,07
STM4573	stjC	1,11	0,08	1,09	0,09

STM4591	sthE	1,21	0,07	1,09	0,09
STM4592	sthD	0,64	0,07	3,37	0,01
STM4594	sthA	0,67	0,01	0,78	0,00
**Cell envelope biogenesis and OM**					
STM0047	lspA	1,03	0,09	0,66	0,02
STM0074	caiT	1,05	0,08	1,57	0,04
STM0093	imp	1,01	0,09	1,07	0,07
STM0120	yabC	0,99	0,08	0,72	0,01
STM0122	ftsI	0,64	0,01	0,77	0,03
STM0123	murE	1,27	0,04	1,37	0,07
STM0124	murF	1,01	0,09	0,75	0,01
STM0125	mraY	0,92	0,07	0,65	0,01
STM0126	murD	0,88	0,04	0,73	0,00
STM0128	murG	0,92	0,04	0,68	0,01
STM0129	murC	0,82	0,03	0,77	0,01
STM0130	ddlB	1,09	0,03	0,63	0,03
STM0131	ftsQ	1,16	0,04	0,58	0,01
STM0134	lpxC	1,77	0,02	1,37	0,07
STM0190	mrcB	2,67	0,00	0,74	0,05
STM0223	yaeL	1,33	0,00	1,55	0,06
STM0224	yaeT	1,23	0,00	0,77	0,03
STM0225	hlpA	1,35	0,01	0,93	0,09
STM0226	lpxD	1,21	0,03	0,69	0,03
STM0228	lpxA	1,33	0,07	0,90	0,09
STM0229	lpxB	1,01	0,08	1,18	0,00
STM0260	dniR	1,19	0,00	1,02	0,10
STM0292		2,10	0,01	0,64	0,07
STM0306		1,19	0,08	2,76	0,03
STM0320	phoE	0,87	0,08	0,98	0,10
STM0346		0,84	0,04	0,62	0,03
STM0350		1,13	0,08	1,17	0,09
STM0352		3,65	0,02	0,14	0,01
STM0373	yaiU	0,68	0,03	1,24	0,06
STM0380	ddlA	1,43	0,02	0,50	0,05
STM0413	tsx	0,75	0,02	0,45	0,00
STM0445	yajG	1,44	0,01	1,15	0,07
STM0476	acrA	3,53	0,03	0,12	0,01
STM0509		0,43	0,01	1,48	0,07
STM0558	yfdH	0,36	0,04	1,16	0,08
STM0569	ybdG	0,62	0,01	0,87	0,04
STM0572		0,23	0,03	0,71	0,07
STM0573		1,31	0,01	0,61	0,05
STM0589	fepE	0,88	0,04	0,87	0,07
STM0637	dacA	2,30	0,01	1,19	0,05

STM0640	mrdA	0,12	0,02	2,08	0,04
STM0647	rlpB	1,02	0,09	0,84	0,05
STM0666	lnt	1,50	0,00	1,91	0,02
STM0719		2,20	0,00	2,22	0,04
STM0721		0,86	0,06	1,36	0,02
STM0725		0,36	0,00	2,06	0,04
STM0747	tolA	0,58	0,05	1,03	0,10
STM0749	pal	1,01	0,09	0,63	0,00
STM0749	pal				
STM0776	galE	0,85	0,00	0,50	0,00
STM0818		1,35	0,02	0,88	0,06
STM0827	ybiO	1,26	0,07	0,68	0,05
STM0833	ompX	1,39	0,06	0,64	0,06
STM0863	dacC	1,13	0,02	0,79	0,02
STM0920					
STM0932		0,90	0,01	0,83	0,07
STM0933	ybjT	0,85	0,08	0,56	0,01
STM0941	ybjY	1,08	0,06	0,69	0,02
STM0961	lolA	0,89	0,07	0,77	0,08
STM0985	lpxK	0,59	0,05	1,42	0,06
STM0988	kdsB	0,89	0,08	0,76	0,08
STM1043		0,68	0,02	1,06	0,09
STM1070	ompA	5,10	0,02	0,12	0,01
STM1139	csgG	0,81	0,06	1,44	0,05
STM1151	mdoH				
STM1155	htrB	0,58	0,06	1,89	0,04
STM1182	flgJ	0,84	0,02	6,84	0,02
STM1208	ycfN	0,83	0,03	0,50	0,01
STM1217	ycfU	1,56	0,02	1,69	0,01
STM1219	ycfW	0,61	0,00	1,10	0,07
STM1246	pagC	1,54	0,00	0,79	0,08
STM1260		0,82	0,03	1,02	0,09
STM1286	mipA	1,27	0,04	1,26	0,08
STM1327	ydiY	1,32	0,01	4,27	0,00
STM1343	nlpC	0,91	0,06	0,89	0,08
STM1376	lppB	1,12	0,09	0,76	0,08
STM1377	lpp	0,75	0,02	0,67	0,02
STM1427	cfa	1,10	0,03	0,65	0,01
STM1432	ydhO	1,42	0,03	1,27	0,08
STM1445	slyB	0,90	0,04	0,53	0,01
STM1473	ompN	0,12	0,01	1,27	0,08
STM1493		0,91	0,06	0,71	0,07
STM1505	rspA	1,06	0,09	1,35	0,07
STM1530		3,34	0,02	0,20	0,01
STM1540		1,17	0,06	0,65	0,05
STM1572	nmpC	1,07	0,02	0,73	0,01

STM1590	yncA	1,40	0,06	0,84	0,08
STM1599	pdgL	1,24	0,01	0,95	0,08
STM1663	ynaI	2,68	0,03	0,75	0,07
STM1681	ycjG	0,41	0,05	1,55	0,08
STM1732	ompW	0,79	0,01	0,91	0,04
STM1737	tonB	2,10	0,00	3,21	0,03
STM1752	galU	1,32	0,04	1,56	0,06
STM1772	kdsA	1,61	0,02	1,32	0,07
STM1778	lolB	1,00	0,10	0,53	0,01
STM1799	emtA	1,06	0,03	0,90	0,05
STM1802	dadX	1,15	0,05	0,62	0,00
STM1819	slp	0,72	0,03	1,56	0,00
STM1836		0,31	0,00	6,03	0,01
STM1845	prc	1,31	0,00	0,31	0,02
STM1889	msbB	1,52	0,02	0,37	0,01
STM1890	yebA	0,92	0,09	1,19	0,02
STM1910		2,74	0,03	0,15	0,01
STM1940		2,54	0,02	17,97	0,01
STM2062	dacD	1,37	0,06	1,88	0,01
STM2070	yeeZ	0,96	0,09	1,29	0,08
STM2079	wzzB	1,08	0,06	0,73	0,04
STM2080	udg	1,46	0,05	0,65	0,02
STM2082	rfbP	0,64	0,04	1,00	0,10
STM2084	rfbM	0,55	0,07	1,19	0,06
STM2085	rfbN	2,55	0,04	0,23	0,01
STM2086	rfbU	11,86	0,00	0,19	0,03
STM2087	rfbV	0,82	0,09	0,27	0,00
STM2089	rfbJ	0,73	0,00	1,20	0,05
STM2090	rfbH	0,92	0,01	0,78	0,02
STM2091	rfbG	1,22	0,01	0,61	0,00
STM2092	rfbF	0,96	0,08	1,15	0,08
STM2094	rfbC	1,19	0,01	0,77	0,02
STM2095	rfbA	0,94	0,07	0,68	0,00
STM2096	rfbD	0,70	0,06	0,26	0,01
STM2097	rfbB	1,38	0,04	1,13	0,09
STM2098	galF	1,02	0,09	0,84	0,07
STM2100	wcaL	0,82	0,01	1,43	0,07
STM2103	wcaJ	0,22	0,00	1,37	0,07
STM2106	wcaI	1,08	0,09	3,06	0,00
STM2108	wcaG	3,68	0,02	0,14	0,01
STM2109	gmd	1,07	0,05	0,51	0,02
STM2113	wcaC	0,80	0,01	1,58	0,04
STM2115	wcaA	1,23	0,08	0,92	0,07
STM2116	wzc	0,98	0,08	0,60	0,02
STM2118	wza	1,10	0,05	2,39	0,04
STM2120	asmA	0,82	0,02	0,68	0,02

STM2165	yehZ	1,50	0,05	0,92	0,09
STM2168	pbpG	1,26	0,02	0,63	0,04
STM2172	yohG	0,67	0,02	1,28	0,03
STM2182	yohK	4,85	0,00	0,98	0,10
STM2214	spr	1,17	0,04	1,60	0,07
STM2267	ompC	1,43	0,00	0,75	0,03
STM2291	yfaW	0,31	0,00	1,18	0,08
STM2297	yfbE	4,04	0,02	0,18	0,01
STM2298	pmrF	0,66	0,07	0,37	0,01
STM2299	yfbG	1,45	0,01	1,03	0,10
STM2301	pqaB	1,88	0,04	1,68	0,07
STM2383	mepA	0,99	0,08	0,52	0,02
STM2392	vacJ	1,07	0,07	0,76	0,04
STM2395	pgtE	1,04	0,08	1,17	0,08
STM2401	ddg	0,71	0,02	0,93	0,09
STM2439	yfeL	0,39	0,03	0,49	0,05
STM2450	amiA	3,94	0,03	1,15	0,09
STM2488	nlpB	3,58	0,03	0,14	0,01
STM2567	yfhD	0,50	0,04	0,70	0,07
STM2591		1,02	0,09	1,29	0,01
STM2690		0,40	0,01	6,04	0,00
STM2692		1,02	0,08	0,76	0,08
STM2756		1,08	0,09	1,09	0,09
STM2831	mltB	1,94	0,01	1,03	0,09
STM2877	iagB	3,25	0,03	0,19	0,01
STM2914		0,34	0,05	1,15	0,09
STM2925	nlpD	1,38	0,06	0,76	0,08
STM2960	gudD	0,83	0,02	18,73	0,00
STM2961	ygcY	0,22	0,03	0,43	0,05
STM2988	mltA	0,81	0,03	0,99	0,10
STM2991	amiC	0,95	0,08	0,68	0,02
STM3002	lgt	0,49	0,04	0,95	0,09
STM3015	ygeA	1,02	0,09	0,44	0,01
STM3031		0,63	0,07	0,27	0,02
STM3038		0,98	0,09	0,84	0,06
STM3067	yggB	1,17	0,03	0,91	0,02
STM3112	mltC	0,47	0,05	3,35	0,01
STM3186	tolC	1,18	0,01	0,88	0,05
STM3200	rfaE	1,08	0,05	0,60	0,01
STM3268	yraR	3,12	0,07	0,67	0,07
STM3300	dacB	1,66	0,01	0,53	0,04
STM3307	murA	0,80	0,02	0,58	0,01
STM3326	mtgA	0,87	0,08	3,03	0,02
STM3372	mreD	2,19	0,05	1,28	0,07
STM3390	acrE	0,70	0,02	1,99	0,00
STM3410	mscL	0,83	0,08	1,89	0,06

STM3587	yhiI	0,72	0,01	0,64	0,01
STM3601		1,08	0,05	2,07	0,05
STM3610	yhjG	0,99	0,10	0,51	0,00
STM3645	yiaD	1,25	0,07	1,06	0,09
STM3697		1,07	0,08	1,52	0,04
STM3707	yibD	0,77	0,02	1,02	0,09
STM3710	rfaD	0,82	0,08	1,18	0,09
STM3711	rfaF	0,74	0,08	1,12	0,08
STM3712	rfaC	0,59	0,01	0,63	0,05
STM3713	rfaL	0,33	0,05	0,89	0,07
STM3714	rfaK	0,55	0,04	1,30	0,07
STM3717	rfaJ	3,31	0,03	1,61	0,05
STM3718	rfaI	0,70	0,04	0,63	0,01
STM3719	rfaB	1,20	0,01	1,09	0,07
STM3722	rfaG	0,93	0,05	0,58	0,00
STM3723	rfaQ	0,81	0,02	0,65	0,00
STM3724	kdtA	1,43	0,02	0,88	0,07
STM3757	misL				
STM3828	dgoA	0,88	0,09	1,35	0,07
STM3833		0,84	0,05	0,68	0,02
STM3861	glmS	0,92	0,07	1,43	0,08
STM3862	glmU	0,93	0,07	0,84	0,06
STM3873	gidB	0,98	0,09	1,18	0,04
STM3918	rfe	0,94	0,08	0,72	0,02
STM3919	wzzE	0,76	0,04	1,42	0,05
STM3920	wecB	0,82	0,05	1,32	0,08
STM3921	wecC	0,69	0,03	0,42	0,01
STM3922	rffG	0,78	0,04	0,74	0,02
STM3923	rffH	0,64	0,01	1,12	0,08
STM3925	wecE	0,65	0,03	0,41	0,01
STM3929	wecG	0,63	0,02	0,35	0,01
STM3957	pldA	0,81	0,00	0,91	0,06
STM4102		2,27	0,05	1,73	0,03
STM4137	murB	0,81	0,03	1,01	0,10
STM4205		0,96	0,07	2,06	0,05
STM4217		1,08	0,05	0,55	0,02
STM4236	dgkA	0,84	0,01	1,00	0,10
STM4247	alr	0,60	0,01	0,56	0,01
STM4259		0,99	0,09	0,55	0,02
STM4260		1,12	0,03	2,97	0,04
STM4272		1,43	0,02	1,22	0,08
STM4339	blc	0,97	0,10	0,80	0,08
STM4347	yjeP	0,41	0,02	0,99	0,10
STM4358	amiB	0,99	0,10	1,41	0,07
STM4396	ytfB	2,57	0,03	0,18	0,01
STM4401	ytfG	0,95	0,08	1,25	0,01

STM4409	ytfM	1,33	0,02	1,31	0,07
STM4416	mpl	0,73	0,01	0,60	0,00
STM4510		1,06	0,07	1,44	0,08
STM4539		0,96	0,00	1,79	0,07
STM4540		0,49	0,02	2,84	0,01
STM4541	mdoB	0,28		0,62	0,06
STM4582	slt	0,75	0,08	0,67	0,03

Coenzyme metabolism

STM0008	mog	0,75	0,02	1,24	0,03
STM0045	ribF	1,03	0,09	1,58	0,07
STM0062	citX2	1,12	0,03	0,79	0,05
STM0063	citG2	0,75	0,00	1,75	0,05
STM0087	folA	0,65	0,05	0,79	0,07
STM0091	pdxA	1,67	0,01	1,51	0,06
STM0106	yabJ	0,88	0,06	1,44	0,01
STM0140	yacE	1,20	0,05	1,63	0,06
STM0145	nadC	1,36	0,07	0,82	0,08
STM0163		11,62	0,00	0,85	0,01
STM0180	panD	1,20	0,04	0,77	0,04
STM0181	panC	0,39	0,00	9,80	0,00
STM0182	panB	1,68	0,04	2,68	0,02
STM0183	folK	1,12	0,05	4,06	0,02
STM0202	hemL	1,08	0,07	0,92	0,08
STM0372	hemB	0,83	0,08	1,00	0,10
STM0416	ribD	0,81	0,08	1,03	0,10
STM0417	ribH	0,82	0,06	0,77	0,08
STM0422	dxs	0,91	0,09	0,71	0,06
STM0423	ispA	1,11	0,03	0,88	0,06
STM0434	apbA	1,10	0,09	0,44	0,00
STM0439	cyoE	1,26	0,00	0,73	0,02
STM0542	folD	0,87	0,03	0,56	0,00
STM0595	entC	0,45	0,02	1,90	0,05
STM0619	citG	0,44	0,00	0,57	0,02
STM0620	citX	1,06	0,08	1,42	0,04
STM0633	lipA	1,16	0,03	0,72	0,02
STM0635	lipB	0,60	0,00	2,36	0,04
STM0645	nadD	0,98	0,09	1,82	0,06
STM0671	ubiF	0,92	0,03	0,81	0,01
STM0756	nadA	0,85	0,02	0,56	0,06
STM0757	pnuC	1,43	0,03	0,82	0,00
STM0769		0,97	0,08	1,05	0,08
STM0793	bioA	0,90	0,07	0,48	0,00
STM0794	bioB	1,22	0,05	1,58	0,07
STM0795	bioF	1,00	0,10	0,74	0,02
STM0797	bioD	0,95	0,09	0,95	0,07

STM0802	moaA	2,56	0,00	1,25	0,06
STM0804	moaC	0,91	0,03	0,72	0,02
STM0805	moaD	3,58	0,01	2,72	0,04
STM0806	moaE	0,74	0,04	3,77	0,03
STM0845	moeB	3,99	0,03	16,30	0,00
STM0846	moeA	1,64	0,06	2,21	0,05
STM0875	rimK	0,88	0,07	1,71	0,02
STM0977	serC	1,04	0,08	0,97	0,08
STM1004	pncB	0,73	0,02	0,74	0,02
STM1310	nadE	0,98	0,07	0,73	0,01
STM1340	btuC	0,89	0,05	20,78	0,00
STM1342	btuD	0,97	0,09	0,83	0,07
STM1426	ribE	1,09	0,05	0,94	0,09
STM1448	pdxH	0,86	0,05	0,50	0,01
STM1450	pdxY	1,02	0,09	0,56	0,02
STM1489	ynfK	0,75	0,01	0,80	0,05
STM1546		1,95	0,00	2,23	0,04
STM1711	ribA	3,63	0,03	0,14	0,01
STM1718	btuR	0,85	0,08	1,09	0,09
STM1777	hemA	0,96	0,09	0,66	0,03
STM2016	cobT	0,20	0,01	3,06	0,01
STM2017	cobS	1,10	0,08	0,61	0,01
STM2018	cobU	0,77	0,00	0,65	0,01
STM2019	cbiP	1,47	0,01	0,69	0,08
STM2024	cbiL	0,79	0,02	0,89	0,01
STM2025	cbiK	1,39	0,02	1,26	0,08
STM2026	cbiJ	2,51	0,02	0,19	0,00
STM2027	cbiH	1,22	0,07	0,66	0,04
STM2028	cbiG	1,04	0,07	0,84	0,08
STM2029	cbiF	0,70	0,06	1,31	0,08
STM2030	cbiT	3,22	0,02	0,14	0,00
STM2031	cbiE	0,97	0,09	0,63	0,02
STM2032	cbiD	1,19	0,04	0,82	0,08
STM2033	cbiC	0,58	0,04	1,53	0,07
STM2034	cibB	1,79	0,05	0,16	0,00
STM2093	rfbI	1,00	0,10	0,72	0,07
STM2146	thiD	1,26	0,08	0,62	0,00
STM2147	thiM	1,98	0,07	1,74	0,06
STM2175		6,52	0,00	0,41	0,05
STM2193	folE	1,80	0,02	1,52	0,07
STM2266	apbE	0,83	0,08	1,26	0,04
STM2276	ubiG	0,55	0,01	1,29	0,04
STM2306	menC	0,92	0,07	0,51	0,01
STM2307	menB	1,04	0,08	0,65	0,01
STM2310	menF	0,80	0,06	0,59	0,07
STM2356	ubiX	0,90	0,07	0,50	0,01

STM2365	folC	0,97	0,09	0,76	0,00
STM2435	pdxK	2,21	0,04	1,78	0,05
STM2451	hemF	0,88	0,06	0,89	0,07
STM2549	asrB	0,45	0,02	0,97	0,10
STM2573		1,16	0,04	0,77	0,07
STM2578	pdxJ	1,09	0,02	0,62	0,01
STM2641	nadB	1,13	0,04	0,19	0,03
STM2818	gshA	1,30	0,07	1,30	0,08
STM2921		0,91	0,09	0,80	0,08
STM2922		0,28	0,03	4,27	0,01
STM2949	ptpS	1,29	0,07	0,98	0,10
STM2987	ygdL	0,83	0,04	0,85	0,06
STM3056	visC	1,31	0,01	0,79	0,04
STM3057	ubiH	1,09	0,04	0,73	0,02
STM3061	ygfA	0,96	0,08	1,32	0,07
STM3090	metK	0,90	0,03	1,48	0,07
STM3095	gshB	1,04	0,08	0,69	0,00
STM3104	yggW	0,69	0,04	0,97	0,09
STM3195	ribB	0,90	0,05	1,08	0,07
STM3206	folB	0,32	0,01	0,75	0,06
STM3295	folP	1,08	0,08	1,02	0,09
STM3305	ispB	1,11	0,01	0,99	0,10
STM3350		0,94	0,06	0,43	0,01
STM3382	panF	2,45	0,03	0,94	0,06
STM3477	cysG	1,11	0,06	0,71	0,01
STM3583	acpT	1,01	0,09	1,84	0,00
STM3709	kbl	0,94	0,08	1,21	0,08
STM3725	kdtB	1,53		0,63	
STM3730	dfp	1,23	0,08	0,74	0,07
STM3849	yieE	1,38	0,08	0,87	0,09
STM3911					
STM3936	hemX	0,73	0,02	1,09	0,08
STM3937	hemD	0,87	0,06	1,16	0,09
STM3938	hemC	1,05	0,09	0,43	0,01
STM3970	ubiE	1,76	0,02	1,39	0,07
STM3978	yigC	0,56	0,03	1,05	0,10
STM3979	ubiB	0,71	0,07	0,90	0,09
STM3993	mobB	1,06	0,02	0,72	0,03
STM3994	mobA	0,53	0,02	1,25	0,02
STM4004	hemN			0,20	
STM4012		0,11	0,01	2,03	0,06
STM4089	menG	3,18	0,03	0,14	0,01
STM4090	menA	0,79	0,08	1,69	0,03
STM4130	btuB	1,10	0,09	1,49	0,02
STM4138	birA	0,89	0,03	0,77	0,03
STM4139	coaA	2,53	0,00	0,97	0,10

STM4159	thiH	1,08	0,03	0,68	0,00
STM4161		1,27	0,03	1,21	0,06
STM4162	thiF	1,51	0,02	0,82	0,06
STM4163	thiE	1,84	0,01	0,67	0,03
STM4164	thiC	1,50	0,02	0,67	0,02
STM4167	hemE	0,99	0,09	1,16	0,06
STM4233	ubiC	0,79	0,02	0,78	0,02
STM4234	ubiA	0,42	0,01	2,12	0,00
STM4576	lplA	0,93	0,09	1,19	0,07

Drug/analog resistance

STM0090	ksgA	3,95	0,02	0,24	0,01
STM0147	ampE	0,80	0,06	0,64	0,03
STM0376	sbmA	0,79	0,06	0,88	0,09
STM0444	ampG	0,63	0,00	7,62	0,02
STM0476	acrA	3,53	0,03	0,12	0,01
STM0477	acrR	0,74	0,07	1,30	0,09
STM0503	ybbM	1,40	0,06	1,19	0,07
STM0578	nfnB	3,31	0,03	0,15	0,00
STM1518	marB	0,25	0,00	0,46	0,04
STM1519	marA	0,97	0,10	5,76	0,03
STM1520	marR	5,15	0,03	0,78	0,08
STM1522	ydeA				
STM1608	tehB	0,79	0,03	0,55	0,00
STM1609	tehA	1,22	0,06	1,01	0,09
STM2184	sanA	1,17	0,03	0,72	0,03
STM2221	bcr	0,47	0,06	0,76	0,08
STM2304	pmrD	1,28	0,00	0,82	0,05
STM2814	emrA	0,49	0,04	1,12	0,09
STM2815	emrB	0,83	0,07	0,74	0,07
STM3009	ygeD	1,06	0,05	0,33	0,03
STM3015	ygeA	1,02	0,09	0,44	0,01
STM3101	yggT	0,98	0,09	0,79	0,03
STM3205	bacA	1,33	0,05	1,11	0,08
STM3391	acrF	1,10	0,09	0,68	0,02
STM3798	emrD	1,10	0,08	0,55	0,00

Energy production and conversion

STM0056		0,51		1,21	0,08
STM0057		1,11	0,08	1,37	0,07
STM0058	citC2	1,00	0,10	0,55	0,01
STM0059	citD2	0,94	0,05	0,73	0,03
STM0061	citF2	1,28	0,03	1,94	0,07
STM0072	caiB	0,51	0,03	0,35	0,03
STM0075	fixA	3,64	0,02	0,17	0,01
STM0076	fixB	1,00	0,10	2,63	0,03

STM0077	fixC	0,32	0,00	2,58	0,05
STM0078	fixX	0,62	0,03	0,11	0,00
STM0152	aceE	1,61	0,01	1,45	0,07
STM0153	aceF	1,29	0,03	1,21	0,08
STM0154	lpdA	0,83	0,02	0,86	0,05
STM0158	acnB	1,16	0,04	0,49	0,00
STM0360		0,96	0,06	0,77	0,04
STM0361		0,89	0,03	0,77	0,03
STM0369	prpC	0,87	0,06	0,74	0,03
STM0421	yajO	0,98	0,06	0,81	0,05
STM0440	cyoD	1,06	0,08	0,48	0,01
STM0441	cyoC	1,00	0,10	0,97	0,10
STM0442	cyoB			4,18	0,01
STM0443	cyoA	0,91	0,07	0,41	0,00
STM0528	allD	3,10	0,00	2,24	0,04
STM0529	fdrA	1,52	0,04	3,26	0,00
STM0564		1,44	0,07	0,76	0,05
STM0578	nfnB	3,31	0,03	0,15	0,00
STM0611		0,65	0,04	2,61	0,01
STM0612		0,58	0,01	1,62	0,03
STM0621	citF	1,14	0,06	0,90	0,05
STM0623	citD	1,24	0,00	0,64	0,01
STM0627	dcuC	1,06	0,00	1,12	0,06
STM0691		3,32	0,02	0,16	0,01
STM0694	fldA	1,64	0,01	1,08	0,09
STM0730	gltA	1,48	0,00	0,78	0,02
STM0732	sdhC	1,33	0,01	0,51	0,00
STM0733	sdhD	0,64	0,03	0,75	0,03
STM0734	sdhA	1,38	0,03	1,25	0,07
STM0735	sdhB	0,99	0,08	0,72	0,02
STM0737	sucB	1,25	0,01	0,91	0,08
STM0738	sucC	1,38	0,00	1,33	0,07
STM0739	sucD	1,27	0,00	0,57	0,00
STM0740	cydA	0,85	0,07	0,83	0,08
STM0741	cydB	1,04	0,10	0,67	0,06
STM0761		0,15	0,00	6,46	0,01
STM0762		0,78	0,01	1,75	0,03
STM0775	galT	1,22	0,02	1,60	0,07
STM0843	pflF	1,07	0,09	1,23	0,08
STM0855		0,41	0,00	1,64	0,06
STM0856		1,11	0,08	1,22	0,08
STM0858		0,64	0,05	1,83	0,07
STM0874	mdaA	0,31	0,04	1,36	0,08
STM0936	hcr	1,96	0,05	0,82	0,05
STM0937	hcp	1,10	0,05	0,74	0,04
STM0956	cydC	2,71	0,04	0,16	0,00

STM0957	cydD	1,00	0,10	1,32	0,07
STM0964	dmsA	0,83	0,03	0,63	0,01
STM0965	dmsB	1,71	0,05	0,23	0,00
STM0973	pflB	1,00	0,10	0,10	0,02
STM1083	yccX	0,44	0,06	0,69	0,02
STM1124	putA	0,86	0,09	1,18	0,08
STM1211	ndh	0,95	0,07	0,55	0,00
STM1238	icdA	1,50	0,02	1,26	0,07
STM1253		0,90	0,00	0,71	0,00
STM1261		0,31	0,00	3,10	0,02
STM1296	ydjA	0,99	0,10	0,34	0,02
STM1305	astD	1,43	0,03	0,60	0,04
STM1351	ydiT	0,21	0,00	12,72	0,00
STM1352	ydiS	1,09	0,07	2,08	0,00
STM1353	ydiR	5,37	0,03	1,11	0,08
STM1354	ydiQ	1,35	0,06	0,69	0,07
STM1383	ttrA	1,51	0,02	1,04	0,07
STM1436	nemA	3,18	0,03	0,11	0,01
STM1454	ydgQ	2,06	0,06	1,95	0,06
STM1455	ydgP	0,41	0,04	1,00	0,10
STM1456	ydgO	1,49	0,07	0,39	0,04
STM1458	ydgM	1,14	0,08	2,45	0,02
STM1459		0,97	0,09	5,18	0,00
STM1468	fumA	0,83	0,00	0,91	0,04
STM1469	fumC	0,93	0,06	0,59	0,00
STM1479	pntA	1,23	0,02	0,72	0,01
STM1480	pntB	1,33	0,02	0,66	0,01
STM1498		0,12	0,00	7,09	0,01
STM1499		0,48	0,02	0,77	0,01
STM1524	yneI	0,53	0,07	0,85	0,05
STM1533		1,11	0,06	1,13	0,09
STM1536		0,89	0,09	2,61	0,00
STM1537		0,97	0,10	1,54	0,06
STM1538		1,04	0,10	0,82	0,09
STM1539		0,65	0,01	0,49	0,00
STM1556		1,28	0,08	0,09	0,00
STM1566	sfcA	0,93	0,04	0,62	0,01
STM1568	fdnI	0,55	0,02	0,92	0,06
STM1569	fdnH	0,91	0,09	2,27	0,05
STM1570	fdnG	0,91	0,08	0,98	0,09
STM1577	narZ				
STM1579	narW	1,08	0,09	1,22	0,08
STM1580	narV	3,57	0,02	0,24	0,02
STM1597	ydcW	0,87	0,02	0,27	0,03
STM1620		1,15	0,04	2,07	0,05
STM1627		23,49	0,00	1,31	0,08

STM1639	cybB	1,35	0,01	1,83	0,03
STM1647	ldhA	1,41	0,02	1,51	0,07
STM1651	nifJ			4,73	0,00
STM1712	acnA	1,38	0,02	0,97	0,10
STM1749	adhE	1,27	0,05	1,44	0,07
STM1761	narI	2,61	0,00	3,34	0,01
STM1762	narJ	0,82	0,03	0,80	0,02
STM1763	narH	0,75	0,04	0,71	0,04
STM1786		1,11	0,05	2,17	0,05
STM1787		1,35	0,01	1,07	0,09
STM1788		1,09	0,05	1,37	0,08
STM1789		1,02	0,09	1,01	0,10
STM1792		0,82	0,02	1,61	0,03
STM1793		1,20	0,09	0,61	0,05
STM2051	pduP	1,01	0,10	0,76	0,07
STM2052	pduQ	1,30	0,03	0,99	0,09
STM2053	pduS	0,75	0,00	0,55	0,00
STM2057	pduW	2,08	0,01	0,30	0,02
STM2063	phsC	0,76	0,07	3,65	0,00
STM2064	phsB	8,34	0,05	0,74	0,03
STM2065	phsA	1,19	0,04	0,88	0,08
STM2167	dld	1,03	0,09	1,06	0,10
STM2255	napC	0,57	0,00	0,98	0,10
STM2256	napB	1,12	0,08	1,41	0,07
STM2261	napF	3,33	0,02	0,17	0,01
STM2279	yfaE	2,71	0,05	1,61	0,07
STM2282	glpQ	0,65	0,01	0,87	0,05
STM2284	glpA	1,23	0,08	0,77	0,08
STM2286	glpC	0,86	0,05	0,54	0,01
STM2316	nuoN	0,85	0,07	0,91	0,01
STM2317	nuoM	0,90	0,01	1,29	0,07
STM2318	nuoL	0,95	0,04	0,51	0,05
STM2319	nuoK	4,79	0,00	1,72	0,01
STM2320	nuoJ	1,00	0,09	0,52	0,01
STM2321	nuoI	0,99	0,08	0,59	0,00
STM2322	nuoH	0,99	0,10	0,74	0,00
STM2323	nuoG	0,76	0,07	0,94	0,10
STM2324	nuoF	0,99	0,10	0,78	0,08
STM2325	nuoE	0,83	0,08	0,99	0,10
STM2326	nuoC	0,85	0,03	0,65	0,01
STM2327	nuoB	0,97	0,09	0,57	0,00
STM2328	nuoA	1,83	0,02	1,44	0,06
STM2337	ackA	1,40	0,01	0,73	0,03
STM2338	pta	1,57	0,01	2,00	0,05
STM2406		0,71	0,04	0,45	0,05
STM2463	eutE			0,23	0,04

STM2466	eutD	1,80	0,00	2,23	0,04
STM2472	maeB	0,88	0,05	0,57	0,02
STM2527		1,01	0,09	0,81	0,03
STM2529		1,26	0,02	0,36	0,04
STM2530		0,44	0,01	1,13	0,05
STM2538	fdx	0,90	0,05	0,55	0,01
STM2542	nifU	1,91	0,02	1,26	0,08
STM2548	asrA	1,00	0,10	0,39	0,02
STM2550	asrC	0,06	0,00	0,78	0,07
STM2556	hmpA	1,22	0,03	1,89	0,06
STM2576	yfhL	1,11	0,04	1,06	0,09
STM2651	yfiQ	0,69	0,02	0,39	0,05
STM2791	gabD	1,07	0,07	0,95	0,09
STM2840		0,20	0,03	1,36	0,06
STM2843	hydN	1,07	0,08	1,81	0,02
STM2845	hycI	1,07	0,09	0,54	0,04
STM2847	hycG	0,91	0,04	15,09	0,00
STM2848	hycF	0,70	0,03	0,51	0,01
STM2849	hycE	0,69	0,01	0,96	0,09
STM2850	hycD	0,64	0,05	1,86	0,06
STM2851	hycC	1,15	0,03	0,72	0,04
STM2852	hycB	0,95	0,07	2,66	0,05
STM2963		1,01	0,09	0,76	0,01
STM2973	fucO	1,85	0,04	0,68	0,03
STM3008	tas	1,04	0,08	0,63	0,00
STM3045	fldB	0,89	0,05	0,71	0,03
STM3081		0,10	0,00	0,61	0,07
STM3118		1,57	0,07	2,41	0,02
STM3129		1,07	0,09	2,28	0,00
STM3146	hybD	0,74	0,02	0,60	0,02
STM3147	hybC	0,80	0,04	0,80	0,04
STM3149	hybA	0,87	0,05	0,91	0,09
STM3150	hypO	0,81	0,05	0,78	0,08
STM3164	yqhD	1,48	0,03	1,28	0,07
STM3168	ygiR	0,72	0,07	1,78	0,03
STM3241	tdcE	0,25	0,00	3,58	0,01
STM3242	tdcD	0,62	0,05	4,17	0,03
STM3276	yhbW	0,92	0,08	0,74	0,02
STM3353	oadG	3,28	0,03	0,14	0,01
STM3354		1,03	0,08	0,52	0,02
STM3355		1,41	0,00	0,20	0,01
STM3376	yhdH	0,76	0,03	0,80	0,06
STM3464	prkB	0,58	0,02	0,68	0,06
STM3474	nirB	0,61	0,06	2,73	0,03
STM3500	pckA	1,06	0,06	0,74	0,04
STM3526	glpD	0,72	0,07	0,96	0,10

STM3529		0,55	0,05	0,57	0,01
STM3553	ugpQ	1,68	0,00	0,80	0,00
STM3597	gor	1,23	0,03	1,28	0,08
STM3614	dctA	1,20	0,06	1,53	0,02
STM3644	bisC	1,35	0,03	0,97	0,08
STM3646	yiaE	0,53	0,01	0,72	0,06
STM3666	ysaA	0,93	0,07	0,61	0,01
STM3668	yiaK	1,07	0,09	4,68	0,00
STM3680	aldB	0,92	0,06	0,87	0,08
STM3692	lldP	1,03	0,09	1,24	0,05
STM3694	lldD	1,51	0,01	0,88	0,05
STM3700	gpsA	1,26	0,04	1,10	0,08
STM3822	torA	0,86	0,04	3,45	0,02
STM3823	torC	4,56	0,05	0,70	0,02
STM3864	atpC	0,92	0,08	0,59	0,01
STM3865	atpD	1,19	0,01	0,82	0,01
STM3866	atpG	1,41	0,00	0,74	0,02
STM3867	atpA	1,08	0,07	1,61	0,06
STM3868	atpH	1,15	0,05	0,46	0,00
STM3869	atpF	0,94	0,08	0,71	0,02
STM3870	atpE	0,60	0,01	0,75	0,02
STM3871	atpB	1,05	0,08	1,32	0,07
STM3872	atpI	1,24	0,01	0,73	0,05
STM3875	mioC	1,01	0,10	1,43	0,08
STM3987	hemG	0,56	0,05	1,25	0,09
STM4035	fdoI	1,32	0,02	0,98	0,10
STM4036	fdoH	0,82	0,08	0,90	0,09
STM4037	fdoG	1,85	0,04	0,29	0,02
STM4038	fdhD	0,87	0,03	0,58	0,00
STM4044		1,97	0,06	0,67	0,00
STM4084	fpr	0,84	0,08	0,93	0,09
STM4114	pflD	0,88	0,03	1,99	0,05
STM4119	ppc	1,07	0,05	0,79	0,03
STM4126	udhA	0,93	0,07	1,34	0,08
STM4183	aceB	3,18	0,03	0,18	0,01
STM4184	aceA	0,68	0,00	0,73	0,02
STM4245	qor	0,90	0,05	0,72	0,01
STM4279	nrfC	0,87	0,06	0,64	0,02
STM4283	gltP	1,41	0,01	0,82	0,04
STM4285	fdhF	1,74	0,07	0,52	0,03
STM4300	fumB	0,96	0,08	0,81	0,03
STM4305		0,15	0,02	1,65	0,06
STM4306		0,10	0,00	1,94	0,05
STM4340	frdD	0,41	0,03	0,47	0,01
STM4341	frdC	2,15	0,04	0,16	0,00
STM4342	frdB	2,03	0,05	0,14	0,01

STM4343	frdA	0,74	0,07	0,96	0,10
STM4355	yjeS	0,75	0,04	0,72	0,06
STM4414	ppa	1,10	0,09	0,76	0,08
STM4421		0,84	0,06	0,66	0,02
STM4439	cybC	1,25	0,02	1,51	0,07
STM4519		2,01	0,02	0,79	0,08

Global regulatory function

STM0049	lytB	1,16	0,02	0,87	0,07
STM0397	phoB	2,91	0,03	0,14	0,01
STM0398	phoR	0,65	0,05	0,41	0,01
STM0450	lon	0,80	0,08	1,06	0,10
STM0600	cstA	0,93	0,06	0,88	0,01
STM0693	fur	1,12	0,06	0,72	0,07
STM0702	kdpE	4,09	0,02	0,23	0,02
STM0703	kdpD	0,68	0,06	1,25	0,09
STM0831	dps	1,41	0,01	0,76	0,04
STM1230	phoQ	3,45	0,04	4,95	0,02
STM1231	phoP	3,89	0,00	1,37	0,06
STM1311	osmE	1,05	0,06	0,46	0,00
STM1471	rstB	1,09	0,09	1,50	0,05
STM1505	rspA	1,06	0,09	1,35	0,07
STM1506	rspB	3,66	0,02	0,16	0,01
STM1660	fnr	0,99	0,10	0,39	0,03
STM2546	suhB	1,18	0,08	0,69	0,08
STM2580	era	1,30	0,01	0,61	0,00
STM2637	rseC	0,40	0,00	19,60	0,00
STM2638	rseB	1,70	0,00	0,73	0,04
STM2639	rseA	1,42	0,00	1,71	0,07
STM2640	rpoE	3,84	0,02	0,13	0,01
STM2826	csrA	0,88	0,01	0,73	0,07
STM2924	rpoS	2,38	0,02	0,28	0,03
STM2956	relA	1,62	0,02	1,26	0,08
STM2958	barA	0,79	0,02	0,99	0,09
STM3211	rpoD	0,73	0,01	0,63	0,02
STM3320	rpoN	1,18	0,05	1,37	0,07
STM3321	yhbH	1,00	0,10	0,78	0,08
STM3328	arcB	3,29	0,04	0,30	0,01
STM3341	sspB	0,86	0,04	0,53	0,00
STM3342	sspA	1,21	0,05	1,34	0,08
STM3466	crp	1,16	0,08	1,02	0,09
STM3501	envZ	1,98		7,95	
STM3502	ompR	0,65	0,04	0,99	0,10
STM3568	rpoH	1,00	0,10	0,49	0,01
STM3742	spoT	0,58	0,04	1,30	0,02
STM3853	phoU	0,93	0,08	0,40	0,02

STM3939	cyaA	0,93	0,07	0,68	0,02
STM4058	cpxA	0,67	0,05	1,19	0,07
STM4094	cytR	1,22	0,01	0,71	0,03
STM4125	oxyR	0,54	0,01	0,91	0,07
STM4237	lexA	0,76	0,01	0,97	0,09
STM4265	soxS	0,49	0,01	0,44	0,00
STM4266	soxR	0,92	0,08	1,02	0,09
STM4588	creB	3,26	0,04	1,13	0,07
STM4589	creC	1,22	0,08	1,29	0,07
STM4598	arcA	0,76	0,03	1,43	0,06

**Inorganic ion transport and metabolism**					
STM0035		3,49	0,00	5,31	0,01
STM0039	nhaA	1,74	0,07	0,87	0,08
STM0084		0,55	0,03	2,46	0,04
STM0086	kefC	0,76	0,01	0,88	0,09
STM0089	apaG	0,74	0,02	0,86	0,03
STM0107	yabK				
STM0171	yadF	1,28	0,03	0,78	0,03
STM0191	fhuA	2,13	0,04	0,86	0,08
STM0192	fhuC	1,07	0,07	1,59	0,04
STM0193	fhuD	1,22	0,05	1,22	0,06
STM0194	fhuB	1,11	0,05	0,71	0,03
STM0206	btuF	0,70	0,04	1,30	0,02
STM0245	yaeC	1,16	0,04	0,70	0,01
STM0246	yaeE				
STM0247	abc	0,25	0,00	0,96	0,09
STM0355		0,16	0,03	10,53	0,01
STM0364	foxA	3,50	0,03	8,49	0,01
STM0425	thiI	1,34	0,03	0,63	0,00
STM0429	phnS	1,23	0,07	0,84	0,08
STM0463	amtB	0,98	0,10	0,65	0,06
STM0492	ybaL	0,59	0,05	0,39	0,04
STM0510	sfbA	0,73	0,02	0,51	0,03
STM0511	sfbB	0,16	0,02	1,77	0,03
STM0512	sfbC	0,56	0,04	1,03	0,10
STM0562		0,53	0,01	1,30	0,09
STM0585	fepA	0,81	0,04	0,48	0,01
STM0586	fes	0,20	0,01	2,30	0,04
STM0590	fepC	0,59	0,06	3,20	0,01
STM0591	fepG	0,92	0,05	0,79	0,04
STM0592	fepD	0,73	0,03	0,79	0,01
STM0594	fepB	0,84	0,06	0,69	0,03
STM0618	citT	0,56	0,06	1,60	0,06
STM0667	ybeX	1,15	0,05	0,58	0,01
STM0693	fur	1,12	0,06	0,72	0,07

STM0704	kdpC	0,86	0,08	1,08	0,09
STM0705	kdpB	1,04	0,09	1,07	0,09
STM0706	kdpA	0,88	0,05	0,65	0,02
STM0758	ybgR	0,50	0,02	0,62	0,06
STM0765		1,10	0,05	2,01	0,05
STM0770		0,28	0,04	0,70	0,07
STM0771		0,89	0,04	7,46	0,02
STM0778	modF	1,02	0,09	0,80	0,05
STM0781	modA	1,45	0,02	1,10	0,09
STM0782	modB	0,64	0,06	5,12	0,01
STM0783	modC	1,73	0,06	1,90	0,05
STM0831	dps	1,41	0,01	0,76	0,04
STM0836	ybiR	1,55	0,00	0,92	0,09
STM0924					
STM0974	focA	1,52	0,00	0,75	0,05
STM1044	sodC	1,08	0,08	0,64	0,01
STM1084	yccK	0,92	0,06	0,60	0,02
STM1150	mdoG	1,29	0,07	0,75	0,08
STM1204	fhuE	3,36	0,02	0,16	0,01
STM1271	yeaR	0,15	0,02	1,30	0,08
STM1278	yeaN				
STM1318	katE	0,68	0,01	0,88	0,05
STM1346	ydiE	0,27	0,06	0,18	0,00
STM1431	sodB	0,88	0,02	0,50	0,00
STM1440	sodC	1,04	0,07	0,66	0,00
STM1482	ydgF	1,15	0,09	1,99	0,01
STM1483	ydgE	1,17	0,03	0,79	0,04
STM1490		0,44	0,03	1,66	0,01
STM1587	yncD	2,14	0,00	1,50	0,03
STM1609	tehA	1,22	0,06	1,01	0,09
STM1622	ydcG	0,89	0,04	0,65	0,00
STM1653		1,09	0,06	1,18	0,09
STM1656		1,71	0,02	1,42	0,07
STM1686	pspE				
STM1731		0,99	0,10	4,68	0,00
STM1741		0,88	0,03	1,15	0,05
STM1765	narK	0,77	0,01	1,47	0,07
STM1769	ychN	0,86	0,01	0,65	0,01
STM1771	chaA	0,81	0,02	0,83	0,01
STM1781	ychM	0,11	0,02	0,87	0,09
STM1801	ycgO	0,96	0,09	1,00	0,10
STM1806	nhaB	0,93	0,08	1,36	0,00
STM1808		0,26	0,03	0,89	0,07
STM1874		1,27	0,03	0,93	0,09
STM1891	znuA	0,64	0,01	0,68	0,07
STM1892	znuC	2,06	0,04	1,21	0,08

STM1893	zunB	1,01	0,10	1,49	0,05
STM1907	cutC	1,49	0,04	0,47	0,03
STM1932	ftnB	0,83	0,08	1,00	0,10
STM1935	ftn	0,93	0,05	0,63	0,02
STM2020	cbiO	1,06	0,03	0,77	0,02
STM2021	cboQ	0,96	0,06	1,53	0,01
STM2022	cbiN	0,40	0,01	0,62	0,00
STM2023	cbiM	0,87	0,02	0,67	0,02
STM2199	cirA	0,62	0,05	1,61	0,07
STM2260	napD	1,12	0,08	1,20	0,08
STM2333	yfbS	0,88	0,07	0,61	0,03
STM2398	pgtC	2,70	0,04	3,00	0,04
STM2404		8,22	0,01	1,52	0,07
STM2408	mntH	0,65	0,05	5,94	0,01
STM2441	cysA	1,31	0,01	0,96	0,09
STM2442	cysW	1,90	0,01	1,20	0,08
STM2444	cysP	1,14	0,02	0,74	0,00
STM2446		1,01	0,09	1,08	0,06
STM2482	yffB	1,09	0,09	0,49	0,02
STM2495	yfgD	0,73	0,04	0,43	0,00
STM2501	ppk	0,88	0,05	0,93	0,08
STM2533	sseA	2,29	0,03	0,16	0,01
STM2679	yfjD	1,21	0,09	3,00	0,02
STM2775	iroD	1,17	0,07	1,21	0,08
STM2777	iroN	3,47	0,02	0,14	0,01
STM2783	nxiA	2,12	0,04	0,19	0,00
STM2798	ygaP	0,11	0,02	0,96	0,07
STM2861	sitA	0,75	0,02	3,51	0,01
STM2862	sitB	1,22	0,08	0,25	0,01
STM2863	sitC	2,43	0,00	2,20	0,04
STM2864	sitD	1,98	0,07	0,49	0,00
STM2933	cysC	1,28	0,02	0,78	0,05
STM2934	cysN	1,40	0,00	0,91	0,08
STM2948	cysJ	1,28	0,03	0,93	0,06
STM3006	ygdQ	1,85	0,03	0,69	0,04
STM3073		2,17	0,00	2,19	0,04
STM3074		1,31	0,08	0,78	0,08
STM3075		1,05	0,09	2,55	0,02
STM3122		0,64	0,06	1,46	0,08
STM3141		1,06	0,10	0,46	0,02
STM3142		0,71	0,01	1,72	0,05
STM3166		2,06	0,01	4,31	0,01
STM3190	ygiE	0,53	0,06	2,15	0,04
STM3214	yqjH	1,07	0,05	1,98	0,06
STM3224	ygjT	1,44	0,07	1,44	0,06
STM3314	yrbG	1,35	0,01	0,98	0,10

STM3356		1,05	0,07	1,80	0,06
STM3409	trkA	0,51	0,05	0,22	0,02
STM3443	bfr	1,11	0,06	1,50	0,06
STM3444	bfd	0,42	0,06	0,44	0,04
STM3449	yheL	1,05	0,09	1,08	0,08
STM3450	yheM	1,17	0,07	0,94	0,08
STM3451	yheN	0,80	0,02	0,55	0,01
STM3457	kefB	1,37	0,02	1,14	0,09
STM3475	nirD	0,63	0,05	1,78	0,00
STM3476	nirC	0,69	0,02	1,33	0,07
STM3505	feoA	0,73	0,02	0,89	0,09
STM3506	feoB	0,80	0,04	0,84	0,03
STM3525	glpE	1,16	0,02	0,98	0,10
STM3528		0,44	0,01	1,28	0,08
STM3576	zntA	0,52	0,06	0,95	0,10
STM3703	yibN	1,46	0,01	0,51	0,01
STM3763	mgtB	1,04	0,08	1,02	0,09
STM3820		0,39	0,05	1,64	0,05
STM3853	phoU	0,93	0,08	0,40	0,02
STM3854	pstB				
STM3855	pstA				
STM3856	pstC				
STM3857	pstS				
STM3880	kup	1,21	0,08	1,88	0,04
STM3943	cyaY	0,83	0,05	0,72	0,03
STM3952	corA	0,82	0,05	0,81	0,01
STM3986	trkH	0,45	0,02	1,16	0,09
STM4055	sodA	1,05	0,08	0,65	0,07
STM4061	yiiP	1,34	0,02	0,58	0,01
STM4063	sbp	1,02	0,09	0,72	0,04
STM4106	katG	1,04		1,26	0,06
STM4189	yjbB	1,62	0,02	1,36	0,07
STM4241	zur	1,47	0,07	0,73	0,03
STM4269	yjcE	0,73	0,00	0,74	0,02
STM4277	nrfA	1,03	0,08	2,13	0,05
STM4280	nrfD	0,71	0,02	0,64	0,01
STM4289	phnA	4,57	0,02	1,33	0,01
STM4324	cutA	0,84	0,01	1,78	0,05
STM4338	sugE	0,81	0,03	1,00	0,10
STM4404	cysQ	1,22	0,00	0,84	0,02

Islands

STM0014		1,07	0,06	0,64	0,02
STM0015		0,87	0,04	2,69	0,05
STM0016		1,00	0,10	1,35	0,05
STM0017		1,00	0,10	1,56	0,04

STM0018		0,12	0,03	0,64	0,00
STM0019		1,08	0,06	0,70	0,03
STM0020		0,91	0,06	2,80	0,05
STM0021	bcfA	1,00	0,10	0,14	0,00
STM0022	bcfB	1,02	0,09	1,67	0,06
STM0023	bcfC	0,80	0,01	7,46	0,02
STM0024	bcfD	0,39	0,00	2,02	0,04
STM0025	bcfE	0,72	0,01	1,54	0,04
STM0026	bcfF	0,23	0,03	0,18	0,00
STM0027	bcfG	1,08	0,05	0,59	0,01
STM0028	bcfH	0,81	0,05	2,21	0,05
STM0029		0,98	0,09	1,38	0,05
STM0030		1,07	0,08	1,14	0,06
STM0031		1,08	0,05	0,61	0,02
STM0032		1,00	0,10	0,90	0,06
STM0033		2,15	0,06	1,52	0,01
STM0034		0,82	0,03	2,46	0,05
STM0035		3,49	0,00	5,31	0,01
STM0036		5,75	0,03	1,12	0,09
STM0037		0,99	0,10	1,63	0,04
STM0038		4,27	0,03	1,27	0,08
STM0052		0,49	0,03	5,23	0,00
STM0053		1,67	0,01	1,54	0,05
STM0055		0,99	0,10	0,98	0,09
STM0056		0,51		1,21	0,08
STM0057		1,11	0,08	1,37	0,07
STM0058	citC2	1,00	0,10	0,55	0,01
STM0059	citD2	0,94	0,05	0,73	0,03
STM0060	citE2	3,37	0,02	0,19	0,01
STM0061	citF2	1,28	0,03	1,94	0,07
STM0062	citX2	1,12	0,03	0,79	0,05
STM0063	citG2	0,75	0,00	1,75	0,05
STM0174	stiH	0,43	0,04	0,35	0,02
STM0175	stiC				
STM0176	stiB	1,07	0,05	0,49	0,02
STM0177	stiA	1,06	0,07	1,03	0,10
STM0195	stfA	2,33	0,00	2,68	0,03
STM0196	stfC	0,74	0,01	1,24	0,07
STM0197	stfD	0,98	0,09	1,21	0,03
STM0198	stfE	0,84	0,04	0,75	0,01
STM0199	stfF	0,72	0,03	0,56	0,02
STM0200	stfG	0,90	0,07	0,80	0,03
STM0266		0,62	0,04	1,74	0,07
STM0267		0,84	0,02	1,34	0,07
STM0269		0,32	0,04	1,29	0,07
STM0270		1,14	0,05	2,36	0,04

STM0271		0,63	0,05	5,77	0,02
STM0273		0,89	0,05	0,72	0,02
STM0274		0,85	0,06	0,67	0,03
STM0275		1,96	0,00	2,53	0,04
STM0276		1,01	0,10	1,09	0,07
STM0277		0,46	0,01	1,33	0,01
STM0278		0,81	0,03	0,84	0,02
STM0279		0,73	0,01	1,62	0,06
STM0280		0,74	0,06	0,58	0,07
STM0281		3,34	0,02	0,17	0,01
STM0282		0,88	0,06	0,68	0,03
STM0284		1,90	0,00	2,53	0,03
STM0286		0,93	0,08	1,27	0,01
STM0287		0,77	0,02	0,78	0,02
STM0288		0,83	0,05	0,69	0,03
STM0289		1,11	0,08	1,17	0,09
STM0290		0,47	0,05	1,04	0,09
STM0292		2,10	0,01	0,64	0,07
STM0293		15,90	0,00	0,42	0,05
STM0294		0,93	0,07	1,33	0,01
STM0295		0,99	0,10	1,33	0,05
STM0296		0,87	0,05	0,74	0,01
STM0297		30,94	0,05		
STM0298		0,77	0,01	1,21	0,06
STM0299	safA	1,10	0,07	1,05	0,08
STM0300	safB	1,98	0,01	2,37	0,04
STM0301	safC	1,02	0,10	1,58	0,02
STM0302	safD	11,54	0,02	0,16	0,01
STM0303	ybeJ	1,42	0,07	0,34	0,00
STM0304	sinR	0,91	0,03	2,81	0,04
STM0305		0,64	0,00	0,91	0,07
STM0306		1,19	0,08	2,76	0,03
STM0307		0,86	0,05	1,20	0,01
STM0329		0,88	0,04	1,41	0,05
STM0330		1,24	0,08	0,16	0,00
STM0331		1,08	0,06	0,51	0,02
STM0332		0,15	0,00	4,70	0,02
STM0333		0,28	0,03	0,31	0,02
STM0334		0,69	0,06	0,24	0,01
STM0335		0,85	0,03	2,46	0,05
STM0336	stbE	0,88	0,08	0,74	0,05
STM0337	stbD	0,95	0,10	0,68	0,05
STM0338	stbC	0,52	0,02	6,27	0,00
STM0339	stbB	1,04	0,07	8,45	0,02
STM0340	stbA	0,64	0,02	0,90	0,09
STM0341		0,37	0,00	1,46	0,04

STM0342		1,03	0,09	1,04	0,09
STM0343		2,12	0,03	1,17	0,08
STM0344		3,09	0,04	0,91	0,06
STM0345		1,01	0,10	1,40	0,01
STM0346		0,84	0,04	0,62	0,03
STM0347		0,67	0,08	0,47	0,04
STM0348		0,21	0,00	4,06	0,06
STM0349		0,76	0,01	1,63	0,06
STM0350		1,13	0,08	1,17	0,09
STM0351		0,79	0,02	0,75	0,02
STM0352		3,65	0,02	0,14	0,01
STM0354		0,35	0,02	1,11	0,06
STM0355		0,16	0,03	10,53	0,01
STM0356		0,01	0,10	4,84	0,04
STM0357	mod	1,00	0,10	0,96	0,04
STM0358	res	1,16	0,06	0,77	0,03
STM0359					
STM0360		0,96	0,06	0,77	0,04
STM0361		0,89	0,03	0,77	0,03
STM0362		0,85	0,09	1,96	0,06
STM0363		1,56	0,05	1,06	0,09
STM0364	foxA	3,50	0,03	8,49	0,01
STM0426	phnV	1,02	0,09	2,09	0,00
STM0427	phnU	0,95	0,07	12,54	0,02
STM0428	phnT	0,72	0,03	0,53	0,01
STM0429	phnS	1,23	0,07	0,84	0,08
STM0430	phnR	1,42	0,03	0,68	0,04
STM0431	phnW	5,55	0,03	2,03	0,04
STM0432	phnX	3,31	0,03	0,14	0,01
STM0543	fimA	1,05	0,02	0,66	0,01
STM0544	fimI	8,45	0,01	1,14	0,09
STM0545	fimC			0,57	0,05
STM0546	fimD	0,93	0,06	3,41	0,01
STM0548	fimF	0,96	0,08	0,70	0,02
STM0549	fimZ	0,79	0,07	0,96	0,09
STM0550	fimY	0,16	0,02	9,19	0,03
STM0551		1,02	0,09	0,46	0,02
STM0552	fimW	0,73	0,03	1,23	0,05
STM0571		1,09	0,07	1,27	0,08
STM0572		0,23	0,03	0,71	0,07
STM0573		1,31	0,01	0,61	0,05
STM0574		0,99	0,09	0,51	0,02
STM0575		1,09	0,05	1,48	0,07
STM0576		0,78	0,01	1,65	0,03
STM0577		1,01	0,10	1,14	0,09
STM0715		0,11	0,02	0,55	0,02

STM0716	0,89	0,04	2,88	0,03
STM0717	0,85	0,03	2,77	0,05
STM0718	0,90	0,06	0,63	0,02
STM0719	2,20	0,00	2,22	0,04
STM0720	1,19	0,06	1,48	0,00
STM0721	0,86	0,06	1,36	0,02
STM0722	0,75	0,02	0,84	0,02
STM0723	0,69	0,04	0,97	0,10
STM0724				
STM0725	0,36	0,00	2,06	0,04
STM0726	0,90	0,07	1,30	0,02
STM0727	0,20	0,02	0,78	0,07
STM0854	0,59	0,04	1,86	0,07
STM0855	0,41	0,00	1,64	0,06
STM0856	1,11	0,08	1,22	0,08
STM0857	2,96	0,02	0,13	0,01
STM0858	0,64	0,05	1,83	0,07
STM0859	4,04	0,01	1,04	0,10
STM0893				
STM0894				
STM0895				
STM0896				
STM0897				
STM0898				
STM0899	1,01	0,10	1,46	0,01
STM0900				
STM0901				
STM0902				
STM0903				
STM0904				
STM0905				
STM0906				
STM0907	0,87	0,03	1,03	0,09
STM0908	0,25			
STM0909	1,69	0,04	0,95	0,08
STM0910				
STM0911				
STM0912				
STM0914				
STM0915				
STM0916				
STM0917				
STM0918				
STM0919				
STM0920				
STM0921				

STM0922		0,23	0,00	1,45	0,02
STM0923					
STM0924					
STM0925					
STM0926		2,09	0,01	0,36	0,04
STM0927		0,86	0,03	0,95	0,08
STM0928	nanH	0,92	0,07	1,30	0,08
STM0929					
STM0930	orfB	1,04	0,09	1,38	0,08
STM0931	ybjR	1,00	0,10	0,49	0,01
STM1005		0,82	0,08	0,96	0,10
STM1006		0,86	0,00	1,71	0,05
STM1007		0,97	0,08	1,62	0,07
STM1008		1,04	0,09	1,18	0,07
STM1009		1,09	0,06	2,35	0,04
STM1011		2,50	0,04	0,58	0,02
STM1012		1,04	0,06	0,78	0,06
STM1013		1,03	0,09	0,84	0,02
STM1014		6,53	0,03	0,40	0,04
STM1015		1,03	0,08	0,56	0,02
STM1016		1,06	0,06	2,09	0,05
STM1017		0,85	0,03	0,85	0,06
STM1018		1,02	0,09	0,73	0,03
STM1019		0,52	0,02	0,78	0,05
STM1020		3,69	0,02	0,18	0,01
STM1021		0,92	0,05	2,83	0,02
STM1022					
STM1023					
STM1024				11,39	0,01
STM1025		1,24	0,08	0,48	0,03
STM1026		1,14	0,09	0,37	0,03
STM1027		3,64	0,02	0,17	0,01
STM1028		0,74	0,03	2,61	0,04
STM1029				1,38	
STM1030					
STM1031					
STM1032					
STM1033					
STM1034					
STM1035					
STM1036					
STM1037					
STM1038					
STM1039					
STM1040					
STM1041		0,52	0,00	6,43	0,01

STM1042		1,16	0,03	1,06	0,09
STM1043		0,68	0,02	1,06	0,09
STM1044	sodC	1,08	0,08	0,64	0,01
STM1045		1,85	0,03	0,82	0,02
STM1046		2,93	0,03	0,53	0,02
STM1047		1,01	0,10	2,38	0,05
STM1048		1,03	0,10	0,52	0,05
STM1050		1,48	0,06	1,59	0,04
STM1051	sseI	4,13	0,02	0,16	0,01
STM1052		1,06	0,10	1,55	0,05
STM1053		1,03	0,08	1,04	0,10
STM1054		0,62	0,04	1,80	0,07
STM1055		7,66	0,00	0,34	0,00
STM1056		1,46	0,04	2,49	0,01
STM1087	pipA	0,74	0,03	0,71	0,06
STM1088	pipB	6,91	0,00	0,66	0,03
STM1089		1,05	0,08	1,71	0,02
STM1090	pipC	1,25	0,06	1,29	0,08
STM1091	sopB	0,63	0,04	0,50	0,00
STM1092	orfX	0,52	0,03	0,40	0,06
STM1093					
STM1094	pipD	1,02	0,09	0,56	0,03
STM1113	scsA	1,66	0,06	0,20	0,01
STM1114	scsB	0,65	0,05	1,01	0,10
STM1115	scsC	0,74	0,01	0,86	0,08
STM1116	scsD	1,09	0,09	0,76	0,07
STM1127		0,95	0,09	0,84	0,08
STM1128		0,27	0,00	6,43	0,00
STM1129		1,04	0,09	2,35	0,03
STM1130		0,91	0,04	2,56	0,05
STM1131		1,05	0,08	1,34	0,05
STM1132		0,74	0,01	1,50	0,04
STM1133		0,84	0,01	0,91	0,07
STM1139	csgG	0,81	0,06	1,44	0,05
STM1140	csgF	0,84	0,02	0,68	0,03
STM1141	csgE	0,85	0,04	2,51	0,05
STM1142	csgD	1,09	0,07	2,66	0,01
STM1143	csgB	0,77	0,04	1,42	0,07
STM1144	csgA	0,26	0,02	0,28	0,01
STM1145	csgC	0,74	0,01	1,69	0,03
STM1171	flgN	0,10	0,00	4,35	0,01
STM1172	flgM	0,12	0,05	2,99	0,00
STM1173	flgA	2,46	0,04	0,65	0,03
STM1174	flgB	20,72		0,53	0,02
STM1175	flgC	1,13	0,04	0,68	0,03
STM1176	flgD	0,76	0,01	1,62	0,03

STM1177	flgE	0,86	0,02	3,33	0,01
STM1178	flgF	1,13	0,04	0,62	0,02
STM1179	flgG	0,88	0,04	2,55	0,05
STM1180	flgH	4,02	0,02	1,79	0,06
STM1181	flgI	1,10	0,04	0,66	0,02
STM1182	flgJ	0,84	0,02	6,84	0,02
STM1183	flgK	0,79	0,02	0,61	0,00
STM1184	flgL	0,94	0,10	0,58	0,06
STM1239		0,58	0,03	2,10	0,06
STM1240	envF	1,04	0,07	1,89	0,05
STM1241	msgA	2,43	0,00	2,07	0,01
STM1242	envE	1,03	0,10	15,21	0,00
STM1243		1,85	0,01	1,26	0,03
STM1244	pagD	4,23	0,04	0,36	0,02
STM1246	pagC	1,54	0,00	0,79	0,08
STM1249		1,21	0,04	1,73	0,06
STM1250		0,69	0,01	0,82	0,06
STM1251		0,62	0,00	0,18	0,00
STM1252		0,88	0,02	1,13	0,06
STM1253		0,90	0,00	0,71	0,00
STM1255		0,85	0,02	1,46	0,07
STM1256		1,20	0,07	1,35	0,07
STM1257		3,79	0,02	0,18	0,01
STM1258		0,65	0,05	4,62	0,03
STM1259		0,69	0,01	1,55	0,06
STM1260		0,82	0,03	1,02	0,09
STM1261		0,31	0,00	3,10	0,02
STM1264	aadA	0,84	0,02	0,68	0,01
STM1265		3,06	0,00	0,94	0,09
STM1267		1,27	0,04	0,84	0,09
STM1268		7,98	0,02	11,11	
STM1269		1,51	0,01	1,56	0,07
STM1328		1,62	0,06	0,86	0,08
STM1330		1,00	0,10	2,78	0,04
STM1331		0,72	0,01	0,71	0,02
STM1379	orf48	1,23	0,02	1,04	0,10
STM1380	orf32	1,17	0,05	2,12	0,05
STM1381	orf245	1,08	0,09	1,16	0,09
STM1382	orf408	0,94	0,08	1,61	0,00
STM1383	ttrA	1,51	0,02	1,04	0,07
STM1384	ttrC	0,86	0,03	2,67	0,05
STM1386	ttrS	0,28	0,04	0,86	0,08
STM1387	ttrR	3,25	0,03	1,98	0,05
STM1388	orf70	0,39	0,03	0,50	0,01
STM1389	orf319	1,29	0,07	0,64	0,01
STM1390	orf242	0,84	0,04	0,41	0,02

STM1391	ssrB	0,99	0,09	0,59	0,02
STM1392	ssrA	0,70	0,04	0,65	0,03
STM1393	ssaB	1,09	0,09	0,15	0,01
STM1394	ssaC	1,31	0,06	1,90	0,06
STM1396	ssaE	1,01	0,10	0,95	0,08
STM1397	sseA	0,85	0,06	0,88	0,09
STM1398	sseB	1,31	0,06	0,94	0,09
STM1399	sscA	3,40	0,02	0,22	0,02
STM1400	sseC	1,30	0,03	0,84	0,09
STM1401	sseD	1,50	0,01	0,74	0,04
STM1402	sseE	1,11	0,06	0,55	0,02
STM1403	sscB	0,84	0,05	6,97	0,00
STM1404	sseF	0,66	0,03	1,89	0,06
STM1405	sseG	0,27	0,01	0,77	0,08
STM1406	ssaG	3,55	0,02	0,13	0,02
STM1407	ssaH	0,91	0,09	0,78	0,09
STM1408	ssaI	1,03	0,09	0,95	0,10
STM1409	ssaJ	1,51	0,04	0,71	0,08
STM1411	ssaK	0,23	0,04	0,49	0,03
STM1412	ssaL	1,54	0,01	0,52	0,05
STM1413	ssaM	0,20	0,01	0,20	
STM1414	ssaV	0,32	0,04	0,28	0,00
STM1415	ssaN	0,51	0,00	0,55	0,02
STM1416	ssaO	1,04	0,08	0,47	0,06
STM1417	ssaP	2,29	0,05	3,99	0,02
STM1419	ssaR	1,05	0,09	0,50	0,03
STM1420	ssaS	1,70	0,06	0,28	0,00
STM1421	ssaT	0,59	0,07	0,36	0,01
STM1422	ssaU	0,35	0,00	4,29	0,02
STM1491		0,93	0,07	0,81	0,05
STM1492		1,27	0,08	0,23	0,01
STM1493		0,91	0,06	0,71	0,07
STM1494		2,26	0,00	2,36	0,04
STM1495	ynfI	1,13	0,06	0,69	0,04
STM1528		0,69	0,02	1,42	0,05
STM1529		1,09	0,09	1,32	0,08
STM1530		3,34	0,02	0,20	0,01
STM1531		2,38		37,43	0,05
STM1532		0,72	0,07	2,98	0,02
STM1533		1,11	0,06	1,13	0,09
STM1534		0,89	0,05	1,05	0,08
STM1535		0,90	0,05	0,71	0,01
STM1536		0,89	0,09	2,61	0,00
STM1537		0,97	0,10	1,54	0,06
STM1538		1,04	0,10	0,82	0,09
STM1539		0,65	0,01	0,49	0,00

STM1540		1,17	0,06	0,65	0,05
STM1541		18,59	0,00	0,79	0,08
STM1542		2,73	0,04	1,36	0,06
STM1543		1,08	0,05	1,71	0,06
STM1544	pqaA	1,42	0,07	0,35	0,01
STM1545		0,33	0,03	5,57	0,02
STM1546		1,95	0,00	2,23	0,04
STM1547		10,08	0,04	0,21	0,00
STM1548		0,71	0,02	0,75	0,04
STM1549		1,43	0,07	2,76	0,00
STM1550		0,76	0,08	1,14	0,08
STM1551		1,44	0,03	1,03	0,09
STM1552		1,37	0,07	0,38	0,01
STM1553		0,92	0,08	0,71	0,03
STM1554		0,52	0,03	0,85	0,08
STM1555		1,04	0,09	4,14	0,02
STM1556		1,28	0,08	0,09	0,00
STM1557		0,71	0,01	0,75	0,01
STM1558		0,23	0,04	0,64	0,00
STM1559		0,99	0,10	0,57	0,01
STM1560		7,27	0,00	0,82	0,08
STM1561		3,05	0,00	2,64	0,03
STM1562		0,28	0,04	0,57	0,01
STM1599	pdgL	1,24	0,01	0,95	0,08
STM1600		0,59	0,07	1,98	0,03
STM1601	ugtL	1,04	0,08	0,56	0,02
STM1602	sifB	1,49	0,01	1,22	0,09
STM1629		0,23	0,00	8,49	0,02
STM1630		0,93	0,08	1,70	0,03
STM1631	sseJ	2,41	0,03	1,17	0,09
STM1632		2,00	0,03	0,56	0,02
STM1633		0,90	0,05	10,58	0,02
STM1634		1,02	0,09	1,45	0,04
STM1635		1,11	0,07	1,18	0,06
STM1636		1,04	0,09	1,18	0,08
STM1637		3,30	0,02	0,13	0,01
STM1638		1,78	0,07	1,71	0,06
STM1664		1,58	0,00	0,79	0,06
STM1665		3,58	0,02	0,15	0,01
STM1666		0,44	0,05	0,69	0,03
STM1667		0,72	0,08	1,24	0,09
STM1668		0,73	0,01	1,52	0,06
STM1669		1,18	0,03	2,28	0,04
STM1670		0,99	0,10	1,25	0,08
STM1671		2,80	0,03	0,15	0,01
STM1672		2,10	0,03	1,22	0,09

STM1673		0,32	0,04	0,52	0,06
STM1855	sopE2	0,81	0,08	0,45	0,02
STM1856		0,97	0,08	2,99	0,04
STM1857		1,25	0,02	0,63	0,06
STM1858		0,08	0,00	0,78	0,08
STM1859		0,13	0,00	3,87	0,03
STM1860		1,01	0,10	1,26	0,05
STM1862	pagO	0,68	0,08	0,43	0,04
STM1864		2,42	0,05	0,60	0,02
STM1865		5,65	0,02	0,14	0,00
STM1866		0,83	0,04	0,79	0,08
STM1867	pagK	1,65	0,02	1,86	0,06
STM1868	mig-3	4,26	0,01	3,61	0,02
STM1869		0,90	0,05	2,80	0,05
STM1911		1,00	0,10	0,61	0,02
STM1915	cheZ	0,63	0,01	1,72	0,06
STM1916	cheY	1,57	0,07	10,57	0,00
STM1917	cheB	0,69	0,01	0,45	0,03
STM1918	cheR	0,66	0,02	0,80	0,00
STM1920	cheW	0,92	0,10	0,42	0,03
STM1921	cheA	1,01	0,10	0,54	0,01
STM1922	motB	3,76	0,02	0,17	0,01
STM1923	motA	1,04	0,09	1,59	0,06
STM1924	flhC	0,86	0,07	0,61	0,06
STM1925	flhD	1,01	0,10	1,02	0,10
STM2019	cbiP	1,47	0,01	0,69	0,08
STM2020	cbiO	1,06	0,03	0,77	0,02
STM2021	cboQ	0,96	0,06	1,53	0,01
STM2022	cbiN	0,40	0,01	0,62	0,00
STM2023	cbiM	0,87	0,02	0,67	0,02
STM2024	cbiL	0,79	0,02	0,89	0,01
STM2025	cbiK	1,39	0,02	1,26	0,08
STM2026	cbiJ	2,51	0,02	0,19	0,00
STM2027	cbiH	1,22	0,07	0,66	0,04
STM2028	cbiG	1,04	0,07	0,84	0,08
STM2029	cbiF	0,70	0,06	1,31	0,08
STM2030	cbiT	3,22	0,02	0,14	0,00
STM2031	cbiE	0,97	0,09	0,63	0,02
STM2032	cbiD	1,19	0,04	0,82	0,08
STM2033	cbiC	0,58	0,04	1,53	0,07
STM2034	cibB	1,79	0,05	0,16	0,00
STM2037	pduF	1,15	0,06	0,79	0,03
STM2038	pduA	0,87	0,03	0,52	0,01
STM2039	pudB	1,03	0,09	1,53	0,07
STM2040	pduC	0,99	0,08	0,74	0,01
STM2041	pduD	0,94	0,04	0,69	0,01

STM2042	pduE	0,99	0,09	0,59	0,00
STM2043	pduG	0,87	0,01	0,62	0,01
STM2044	pduH	1,00	0,10	1,54	0,07
STM2045	pduJ	1,07	0,05	0,75	0,03
STM2046	pduK	1,08	0,02	0,70	0,01
STM2047	pduL	0,99	0,09	0,61	0,00
STM2048	pduM	0,73	0,05	1,32	0,08
STM2049	pduN	1,08	0,07	1,11	0,08
STM2050	pduO	0,81	0,04	0,70	0,07
STM2051	pduP	1,01	0,10	0,76	0,07
STM2052	pduQ	1,30	0,03	0,99	0,09
STM2053	pduS	0,75	0,00	0,55	0,00
STM2054	pduT	0,78	0,08	0,27	0,00
STM2055	pduU	0,69	0,08	1,52	0,01
STM2056	pduV	0,67	0,02	0,93	0,03
STM2057	pduW	2,08	0,01	0,30	0,02
STM2058	pduX	1,76	0,05	0,71	0,07
STM2063	phsC	0,76	0,07	3,65	0,00
STM2064	phsB	8,34	0,05	0,74	0,03
STM2065	phsA	1,19	0,04	0,88	0,08
STM2082	rfbP	0,64	0,04	1,00	0,10
STM2083	rfbK	2,58	0,02	0,79	0,06
STM2084	rfbM	0,55	0,07	1,19	0,06
STM2085	rfbN	2,55	0,04	0,23	0,01
STM2086	rfbU	11,86	0,00	0,19	0,03
STM2087	rfbV	0,82	0,09	0,27	0,00
STM2088	rfbX	0,98	0,09	1,00	0,10
STM2089	rfbJ	0,73	0,00	1,20	0,05
STM2090	rfbH	0,92	0,01	0,78	0,02
STM2091	rfbG	1,22	0,01	0,61	0,00
STM2092	rfbF	0,96	0,08	1,15	0,08
STM2093	rfbI	1,00	0,10	0,72	0,07
STM2149	stcD	1,00	0,10	1,30	0,08
STM2150	stcC	0,94	0,09	1,20	0,08
STM2151	stcB	1,05	0,05	0,45	0,02
STM2152	stcA	0,32	0,00	2,11	0,03
STM2175		6,52	0,00	0,41	0,05
STM2176		3,68	0,02	0,14	0,01
STM2177		0,63	0,05	1,69	0,07
STM2178		0,78	0,01	1,45	0,04
STM2179		0,94	0,06	0,93	0,07
STM2180		1,80	0,04	0,67	0,03
STM2230		0,89	0,06	1,62	0,04
STM2231		0,72	0,03	0,64	0,02
STM2232	oafA	1,46	0,05	2,24	0,02
STM2233		0,86	0,02	0,95	0,08

STM2234		0,99	0,08	0,50	0,02
STM2235		2,16	0,00	2,47	0,04
STM2236		0,96	0,09	1,25	0,01
STM2237		0,76	0,01	0,71	0,00
STM2239		1,12	0,04	3,30	0,02
STM2240		0,73	0,04	0,65	0,02
STM2241	sspH2	0,73	0,05	1,02	0,10
STM2242		0,86	0,06	0,65	0,03
STM2243		3,81	0,02	0,13	0,01
STM2244		2,03	0,01	0,29	0,06
STM2340		0,37	0,00	2,06	0,04
STM2341		0,93	0,08	1,37	0,01
STM2342		0,82	0,03	0,84	0,02
STM2343		1,69	0,06	0,65	0,02
STM2344		2,25	0,00	5,91	0,01
STM2345		0,80	0,04	0,49	0,01
STM2357		0,89	0,06	1,19	0,04
STM2358		2,06	0,06	0,68	0,06
STM2359		1,70	0,07	0,41	0,01
STM2360		0,54	0,00	0,53	0,01
STM2361		1,09	0,05	1,73	0,05
STM2395	pgtE	1,04	0,08	1,17	0,08
STM2396	pgtA	0,29	0,00	0,56	0,01
STM2397	pgtB	0,94	0,10	6,64	0,00
STM2398	pgtC	2,70	0,04	3,00	0,04
STM2399	pgtP	3,04	0,03	0,46	0,04
STM2514	ratB	0,76	0,01	2,47	0,00
STM2516	sinI	0,86	0,03	0,96	0,09
STM2518	yfgJ	3,00		0,19	0,01
STM2584	gogB	5,71	0,02	2,68	0,02
STM2585		1,36	0,02	0,64	0,02
STM2586		0,89	0,09	3,07	0,02
STM2587		0,82	0,05	0,76	0,03
STM2590		2,23	0,00	1,96	0,05
STM2591		1,02	0,09	1,29	0,01
STM2592		0,73	0,02	0,80	0,02
STM2593		0,79	0,04	0,70	0,02
STM2594		0,14	0,00	6,86	0,01
STM2595		0,53	0,01	1,38	0,07
STM2596		1,05	0,07	1,01	0,10
STM2597		0,78	0,07	0,39	0,01
STM2598		0,17	0,00	5,15	0,01
STM2599		0,42	0,01	1,01	0,10
STM2600		1,99	0,04	0,44	0,03
STM2602		1,12	0,03	0,52	0,02
STM2603		1,10	0,04	1,96	0,05

STM2604	0,75	0,01	1,67	0,04
STM2605	0,90	0,05	0,95	0,08
STM2606	0,63	0,00	0,38	0,04
STM2607			0,20	
STM2608	0,77	0,01	1,67	0,03
STM2609	0,92	0,06	1,01	0,09
STM2610	0,26	0,04	1,33	0,01
STM2611	1,15	0,04	0,46	0,01
STM2613	0,77	0,02	1,77	0,03
STM2614	1,24	0,07	0,85	0,07
STM2616	1,90	0,04	0,68	0,04
STM2617	1,06	0,07	2,24	0,05
STM2618	0,57	0,05	0,01	0,00
STM2620	0,94	0,02	1,16	0,08
STM2630	0,24	0,01	0,85	0,07
STM2635	1,03	0,09	1,48	0,04
STM2636	2,67	0,00	2,18	0,04
STM2694				
STM2695				
STM2696				
STM2697				
STM2698				
STM2699	0,23	0,03	0,38	0,04
STM2700				
STM2701				
STM2702				
STM2703				
STM2704	0,57	0,05	0,50	0,00
STM2705				
STM2706	0,03	0,00	1,55	0,04
STM2707				
STM2708				
STM2709				
STM2710	0,82	0,03	0,54	0,01
STM2711	1,83	0,03	0,52	0,00
STM2712	1,01	0,09	0,77	0,04
STM2713				
STM2714				
STM2715				
STM2716	1,54	0,02	1,38	0,07
STM2717	0,73	0,05	0,95	0,09
STM2718	1,05	0,03	0,59	0,01
STM2719				
STM2720				
STM2721				
STM2722	1,74	0,02	1,54	0,06

STM2723					
STM2726					
STM2727					
STM2728					
STM2729					
STM2730					
STM2731		0,67	0,01	1,33	0,06
STM2732					
STM2733					
STM2734					
STM2735					
STM2736					
STM2737					
STM2738					
STM2739		0,90	0,03	0,64	0,02
STM2740		1,31	0,01	0,85	0,03
STM2742		0,81	0,03	0,69	0,03
STM2744				0,10	0,01
STM2746		1,39	0,07	1,21	0,09
STM2747		0,46	0,03	1,28	0,02
STM2748		2,46	0,02	0,47	0,00
STM2749		0,81	0,01	1,50	0,06
STM2750		0,67	0,05	1,77	0,07
STM2751		0,79	0,01	1,39	0,07
STM2752		0,51	0,02	1,24	0,08
STM2753		3,75	0,02	0,20	0,01
STM2754		0,41	0,02	3,62	0,03
STM2755		0,74	0,00	1,50	0,06
STM2756		1,08	0,09	1,09	0,09
STM2757		3,69	0,02	0,14	0,01
STM2758		0,66	0,05	1,60	0,07
STM2759		0,77	0,01	1,34	0,08
STM2760		1,10	0,08	1,10	0,09
STM2761		1,14	0,08	1,16	0,09
STM2762		3,46	0,02	0,15	0,01
STM2763		0,69	0,06	1,44	0,08
STM2764		0,75	0,00	1,28	0,08
STM2765		1,07	0,09	1,18	0,08
STM2766		1,31	0,07	0,16	0,01
STM2767		3,39	0,03	0,14	0,01
STM2770	fljA	0,75	0,07	2,28	0,03
STM2771	fljB	0,90	0,00	0,81	0,04
STM2772	hin	1,98	0,00	0,65	0,02
STM2773	iroB	0,96	0,08	1,20	0,05
STM2775	iroD	1,17	0,07	1,21	0,08
STM2776	iroE	0,75	0,02	2,08	0,06

STM2777	iroN	3,47	0,02	0,14	0,01
STM2778		1,16	0,08	1,68	0,02
STM2779		0,94	0,07	0,86	0,09
STM2780		1,65	0,00	0,71	0,08
STM2781	virK	1,31	0,00	0,88	0,06
STM2782	mig-14	0,94	0,08	1,03	0,09
STM2783	nxiA	2,12	0,04	0,19	0,00
STM2784	tctE	0,30	0,01	1,03	0,10
STM2785	tctD	0,54	0,04	5,00	0,03
STM2787		0,34		0,59	0,01
STM2788					
STM2865	avrA	1,06	0,05	1,04	0,09
STM2866	sprB	0,98	0,08	1,79	0,06
STM2867	hilC	0,02	0,00	1,28	0,08
STM2868		1,14	0,07	1,14	0,07
STM2869	orgA	0,64	0,07	0,52	0,03
STM2871	prgK	2,64	0,03	0,53	0,01
STM2872	prgJ	10,67	0,05	0,19	0,01
STM2873	prgI	1,02		0,32	0,07
STM2874	prgH	1,41	0,02	0,67	0,02
STM2875	hilD	0,63	0,01	1,38	0,06
STM2876	hilA	0,09	0,00	0,26	0,00
STM2877	iagB	3,25	0,03	0,19	0,01
STM2878	sptP	1,48	0,05	0,70	0,04
STM2879	sicP	0,88	0,05	0,73	0,07
STM2880		1,58	0,03	1,60	0,06
STM2881	iacP	0,96	0,08	1,78	0,02
STM2882	sipA	0,94	0,08	0,74	0,02
STM2883	sipD	0,78	0,03	1,12	0,07
STM2884	sipC	3,10	0,02	0,21	0,00
STM2885	sipB	0,10			
STM2886	sicA	1,04	0,08	0,81	0,05
STM2887	spaS	0,87	0,07	1,63	0,04
STM2888	spaR	0,77	0,02	2,24	0,05
STM2889	spaQ	2,55	0,00	2,02	0,04
STM2890	spaP	1,15	0,03	0,64	0,02
STM2892	invJ	1,00	0,10	0,94	0,08
STM2893	invI	0,82	0,04	1,35	0,04
STM2894	invC	0,94	0,05	2,27	0,05
STM2895	invB	0,97	0,09	0,70	0,03
STM2896	invA	0,52	0,05	0,66	0,01
STM2897	invE	1,07	0,08	1,23	0,05
STM2899	invF	3,92	0,05	1,45	0,01
STM2900	invH	1,02	0,08	0,47	0,01
STM2901		1,28	0,03	1,14	0,08
STM2902		1,45	0,06	0,85	0,02

STM2903		0,48		0,41	0,05
STM2904		0,75	0,06	0,54	0,04
STM2905		0,65	0,05	1,05	0,09
STM2906				7,81	
STM2907	pphB	0,63	0,04	1,79	0,07
STM2908		0,66	0,07	1,38	0,07
STM2909	mutS			0,25	0,03
STM2911		1,09	0,08	1,19	0,08
STM2912		3,50	0,02	0,13	0,01
STM2913		0,65	0,05	2,34	0,05
STM2914		0,34	0,05	1,15	0,09
STM3025		0,51	0,03	0,31	0,02
STM3026		1,61	0,06	0,82	0,05
STM3027	stdC	0,62	0,00	1,05	0,09
STM3028	stdB	0,92	0,06	1,07	0,05
STM3029	stdA	0,69	0,01	2,61	0,04
STM3030		1,23	0,08	0,66	0,05
STM3031		0,63	0,07	0,27	0,02
STM3033		1,04	0,08	0,46	0,01
STM3034		0,91	0,05	0,52	0,01
STM3036		0,94	0,07	1,84	0,06
STM3079		1,12	0,08	1,20	0,08
STM3081		0,10	0,00	0,61	0,07
STM3082		0,39	0,04	1,56	0,06
STM3083		1,05	0,09	1,15	0,06
STM3084		1,02	0,09	0,68	0,02
STM3117		1,26	0,08	0,15	0,01
STM3118		1,57	0,07	2,41	0,02
STM3119		0,31	0,01	1,14	0,08
STM3120		1,23	0,06	1,19	0,07
STM3121		0,92	0,07	0,73	0,02
STM3122		0,64	0,06	1,46	0,08
STM3123		0,30	0,00	1,52	0,06
STM3124		1,03	0,10	1,42	0,07
STM3125		0,70	0,05	0,15	0,01
STM3126		0,64	0,05	4,81	0,02
STM3127		0,76	0,00	1,77	0,05
STM3128		1,54	0,02	1,59	0,07
STM3129		1,07	0,09	2,28	0,00
STM3130		1,10	0,08	1,12	0,09
STM3131		0,93	0,06	2,55	0,05
STM3132		0,41	0,05	0,36	0,00
STM3133		1,08	0,09	1,34	0,07
STM3134		0,42	0,00	1,08	0,05
STM3191		0,88	0,04	2,43	0,05
STM3192		1,01	0,10	1,49	0,04

STM3193		0,64	0,05	4,77	0,03
STM3194		3,51	0,02	0,15	0,01
STM3195	ribB	0,90	0,05	1,08	0,07
STM3196	yqiC	0,91	0,06	0,66	0,01
STM3197	glgS	1,10	0,05	1,52	0,07
STM3198		0,93	0,09	1,34	0,07
STM3199	yqiK	1,07	0,09	1,97	0,00
STM3200	rfaE	1,08	0,05	0,60	0,01
STM3202	ygiF	0,87	0,04	0,40	0,03
STM3203	ygiM	3,67	0,00	0,91	0,09
STM3204	cca	0,58	0,07	2,18	0,05
STM3205	bacA	1,33	0,05	1,11	0,08
STM3206	folB	0,32	0,01	0,75	0,06
STM3207	ygiH	1,22	0,06	0,29	0,01
STM3208	gcp	0,83	0,04	0,91	0,07
STM3353	oadG	3,28	0,03	0,14	0,01
STM3354		1,03	0,08	0,52	0,02
STM3355		1,41	0,00	0,20	0,01
STM3356		1,05	0,07	1,80	0,06
STM3357		1,02	0,09	0,52	0,02
STM3358		1,21	0,02	1,68	0,00
STM3527		0,95	0,09	0,87	0,09
STM3528		0,44	0,01	1,28	0,08
STM3529		0,55	0,05	0,57	0,01
STM3530		0,58	0,04	0,76	0,04
STM3532		0,69	0,02	0,80	0,07
STM3533		1,07	0,09	0,87	0,09
STM3636	lpfE	1,05	0,07	0,38	0,00
STM3637	lpfD	1,82	0,05	0,42	0,01
STM3638	lpfC	0,87	0,03	2,30	0,05
STM3639	lpfB	0,99	0,10	1,15	0,07
STM3640	lpfA	0,79	0,03	4,12	0,03
STM3752		0,94	0,09	1,13	0,09
STM3753	sugR	0,89	0,03	0,89	0,03
STM3755	rhuM	2,35	0,03	0,48	0,05
STM3756	rmbA	0,98	0,09	1,23	0,06
STM3757	misL				
STM3758	fidL	1,09	0,09	1,65	0,05
STM3759	marT	1,81	0,06	0,75	0,07
STM3761	slsA	0,18	0,00	5,56	0,02
STM3762	cigR	1,34	0,02	0,66	0,00
STM3763	mgtB	1,04	0,08	1,02	0,09
STM3764	mgtC	0,77	0,05	2,81	0,04
STM3766		0,90	0,08	6,47	0,00
STM3767		0,71	0,01	1,66	0,06
STM3768		0,98	0,10	1,21	0,08

STM3769	0,57	0,03	1,49	0,08
STM3770	3,38	0,02	0,13	0,01
STM3771				
STM3772	0,27		4,21	0,05
STM3773	1,54	0,06	0,91	0,02
STM3774	0,86	0,07	1,34	0,07
STM3775	4,39	0,03	0,38	0,03
STM3779	0,72	0,00	1,48	0,07
STM3780	0,92	0,08	1,44	0,07
STM3781	0,65	0,06	1,31	0,08
STM3782	0,85	0,09	0,70	0,07
STM3783	0,16	0,03	10,40	0,00
STM3784	1,92	0,05	0,30	0,02
STM4010	1,02	0,10	2,60	0,03
STM4011	1,24	0,02	0,69	0,06
STM4012	0,11	0,01	2,03	0,06
STM4013	2,65	0,04	0,13	0,01
STM4014	1,19	0,08	0,61	0,02
STM4015	2,28	0,00	2,87	0,03
STM4195	1,84	0,03	0,22	0,01
STM4196	3,55	0,05	0,67	0,03
STM4197	2,23	0,00	2,22	0,04
STM4198	0,91	0,07	1,39	0,01
STM4199	0,75	0,02	0,79	0,01
STM4200	0,77	0,04	1,00	0,10
STM4201	2,12	0,00	2,76	0,03
STM4202	0,96	0,09	1,14	0,05
STM4203	1,41	0,05	0,74	0,01
STM4204	3,64	0,04	0,40	0,01
STM4205	0,96	0,07	2,06	0,05
STM4206	0,75	0,01	1,70	0,03
STM4207	1,14	0,08	2,54	0,04
STM4208	0,95	0,04	0,46	0,01
STM4209	0,78	0,08	0,31	0,03
STM4210	0,80	0,01	1,58	0,04
STM4211	0,70	0,01	1,69	0,04
STM4212	1,04	0,07	0,45	0,01
STM4213	1,00	0,10	2,55	0,04
STM4214	1,05	0,06	0,89	0,07
STM4215	0,84	0,02	1,71	0,03
STM4216	0,92	0,06	1,03	0,09
STM4217	1,08	0,05	0,55	0,02
STM4218	1,10	0,05	1,64	0,06
STM4219	4,39	0,03	0,50	0,04
STM4257	0,73	0,01	1,50	0,05
STM4258	0,83	0,02	1,13	0,06

STM4259		0,99	0,09	0,55	0,02
STM4260		1,12	0,03	2,97	0,04
STM4261		5,35	0,04	1,12	0,05
STM4262		0,75	0,02	1,90	0,03
STM4305		0,15	0,02	1,65	0,06
STM4306		0,10	0,00	1,94	0,05
STM4307		0,40	0,00	1,98	0,02
STM4308		1,09	0,09	0,66	0,04
STM4309		0,59	0,02	0,60	0,02
STM4310		0,60	0,04	3,58	0,03
STM4312		1,09	0,08	1,13	0,09
STM4313		1,06	0,06	0,55	0,02
STM4314		0,79	0,01	1,44	0,07
STM4315		1,04	0,09	1,26	0,08
STM4316		0,71	0,08	0,96	0,09
STM4317		1,07	0,08	1,27	0,08
STM4318		3,63	0,02	0,84	0,05
STM4319	phoN	1,15	0,05	0,88	0,09
STM4320		1,08	0,09	0,50	0,06
STM4418		2,26	0,00	2,37	0,04
STM4419		1,05	0,08	1,20	0,01
STM4420		0,97	0,10	0,99	0,10
STM4421		0,84	0,06	0,66	0,02
STM4422		2,05	0,00	2,29	0,04
STM4423		8,13	0,04	1,24	0,03
STM4424		0,77	0,03	0,72	0,01
STM4425		1,28	0,08	0,37	0,00
STM4426	srfJ	2,67	0,00	2,34	0,04
STM4427		0,52	0,03	1,20	0,02
STM4428		2,80	0,01	0,67	0,01
STM4429		3,80	0,02	0,14	0,01
STM4430		0,70	0,03	0,60	0,02
STM4431		1,06	0,05	2,22	0,05
STM4432		0,34	0,00	1,63	0,04
STM4433		0,70	0,01	0,30	0,01
STM4434		1,01	0,09	0,73	0,05
STM4435		1,14	0,04	2,15	0,05
STM4436		0,79	0,01	1,43	0,04
STM4440		0,86	0,03	1,17	0,03
STM4441		0,41	0,00	0,54	0,02
STM4442		1,01	0,10	2,76	0,04
STM4444		0,71	0,02	2,30	0,00
STM4445		0,68	0,03	2,19	0,05
STM4446		0,39	0,04	2,28	0,04
STM4447		0,57	0,01	1,54	0,03
STM4448		1,52	0,05	0,70	0,05

STM4450		0,54	0,00	0,93	0,09
STM4488		1,24	0,09	0,42	0,00
STM4490		0,86	0,04	0,57	0,01
STM4491		1,01	0,09	1,35	0,07
STM4492		1,32	0,00	0,79	0,04
STM4493		0,91	0,01	0,71	0,02
STM4494		1,12	0,06	0,73	0,03
STM4496		0,91	0,05	1,53	0,06
STM4571		0,60	0,00	0,49	0,06
STM4572	stjB	0,26	0,00	1,39	0,07
STM4573	stjC	1,11	0,08	1,09	0,09
STM4574		0,95	0,04	0,47	0,05
STM4575		2,23	0,03	1,05	0,10
STM4591	sthE	1,21	0,07	1,09	0,09
STM4592	sthD	0,64	0,07	3,37	0,01
STM4594	sthA	0,67	0,01	0,78	0,00
STM4596		0,93	0,09	5,67	0,00

Lipid metabolism

STM0049	lytB	1,16	0,02	0,87	0,07
STM0055		0,99	0,10	0,98	0,09
STM0070	caiD	0,80	0,02	1,56	0,04
STM0071	caiC	0,29	0,00	0,50	0,05
STM0220	dxr	0,81	0,04	0,76	0,02
STM0221	uppS	1,20	0,02	0,79	0,01
STM0222	cdsA	1,57	0,00	0,97	0,06
STM0227	fabZ	1,27	0,02	0,73	0,02
STM0232	accA	1,71	0,01	1,24	0,07
STM0309	yafH	1,45	0,08	2,44	0,03
STM0371	prpE	3,86	0,00	0,96	0,10
STM0376	sbmA	0,79	0,06	0,88	0,09
STM0464	tesB	0,91	0,04	0,73	0,02
STM0490	aes	0,24	0,00	1,44	0,07
STM0519	glxR	3,83	0,02	0,15	0,01
STM0570	apeE	0,79	0,06	0,37	0,01
STM0812	ybhO	0,88	0,03	8,97	0,02
STM0857		2,96	0,02	0,13	0,01
STM0865	ybjG	1,43	0,02	1,21	0,03
STM1067	fabA	0,79	0,05	0,85	0,09
STM1148	ymdC	1,54	0,01	0,40	0,01
STM1192	plsX	0,62	0,03	0,83	0,03
STM1193	fabH	1,22	0,00	1,21	0,06
STM1194	fabD	0,87	0,02	0,83	0,01
STM1197	fabF	0,77	0,07	0,89	0,09
STM1350	ydiD	2,33	0,05	2,11	0,04
STM1356	ydiO	3,60	0,02	0,16	0,01

STM1357	ydiF	1,09	0,07	1,32	0,05
STM1623		0,35	0,05	0,37	0,05
STM1631	sseJ	2,41	0,03	1,17	0,09
STM1642	acpD	0,17	0,03	1,39	0,06
STM1700	fabI	0,94	0,08	0,49	0,01
STM1710	pgpB	1,05	0,09	1,16	0,08
STM1736	yciA	0,68	0,01	0,92	0,08
STM1739	cls	0,11	0,01	0,86	0,08
STM1779	ipk	0,65	0,01	1,08	0,09
STM1818	fadD	0,76	0,08	1,97	0,02
STM1945	pgsA	3,25	0,03	0,20	0,02
STM2140	yegS	1,23	0,02	0,83	0,05
STM2213	yeiU	1,98	0,01	0,98	0,10
STM2232	oafA	1,46	0,05	2,24	0,02
STM2305	menE	0,90	0,06	1,12	0,09
STM2366	accD	0,91	0,07	0,45	0,00
STM2378	fabB	0,94	0,08	0,56	0,00
STM2389	yfcY	0,97	0,08	2,33	0,05
STM2391	fadL	1,02	0,09	0,54	0,02
STM2523	gcpE	0,87	0,09	0,90	0,09
STM2577	acpS	1,18	0,03	0,93	0,06
STM2652	pssA				
STM2687	yfjG	0,97	0,08	0,61	0,01
STM2881	iacP	0,96	0,08	1,78	0,02
STM2918	ygbJ	3,78	0,02	0,14	0,01
STM2929	ispF	1,18	0,08	0,75	0,07
STM2930	ispD	1,01	0,10	0,63	0,00
STM3010	aas	1,47	0,01	1,40	0,02
STM3019	yqeF	1,99	0,04	2,04	0,04
STM3039	idi	1,02	0,10	1,11	0,09
STM3119		0,31	0,01	1,14	0,08
STM3173	plsC	0,38	0,04	1,01	0,10
STM3248	garR	2,08	0,03	0,21	0,00
STM3273	yhbT	1,01	0,10	0,65	0,02
STM3379	accB	0,97	0,09	0,48	0,01
STM3380	accC	0,84	0,04	0,60	0,00
STM3595		0,95	0,07	0,83	0,02
STM3961	pldB	0,61	0,00	0,93	0,06
STM3982	fadA	0,51	0,03	1,13	0,09
STM3983	fadB	0,78	0,08	1,13	0,09
STM3998	yihG	0,32	0,00	2,32	0,01
STM4023	yihU	0,98	0,09	1,13	0,07
STM4032		1,18	0,08	0,80	0,08
STM4064	ushB	0,89	0,02	0,77	0,04
STM4235	plsB	1,10	0,08	0,73	0,03
STM4319	phoN	1,15	0,05	0,88	0,09

STM4348	psd	0,70	0,06	1,04	0,10

**Nucleotide transport and metabolism**					
STM0033		2,15	0,06	1,52	0,01
STM0051	rihC	0,98	0,09	1,61	0,07
STM0141	guaC	1,00	0,10	0,45	0,00
STM0170	hpt	0,35	0,02	1,59	0,05
STM0207	pfs	0,48	0,04	1,31	0,05
STM0208	dgt	1,07	0,06	0,65	0,01
STM0218	pyrH	1,57	0,02	1,22	0,08
STM0317	gpt	0,78	0,03	1,32	0,08
STM0483	apt	1,15	0,08	0,94	0,10
STM0488	adk	0,92	0,09	0,71	0,07
STM0515	allA	0,97	0,09	2,25	0,00
STM0522	allP	2,14	0,00	2,42	0,04
STM0523	allB	0,77	0,02	0,78	0,02
STM0524	ybbY	0,91	0,06	0,64	0,02
STM0533	purK	0,14	0,03	0,32	0,04
STM0533	purK				
STM0534	purE	0,97	0,09	1,10	0,04
STM0534	purE				
STM0661	ybeK	1,07	0,07	0,64	0,01
STM0980	cmk	1,20	0,03	0,57	0,02
STM1058	pyrD	0,99	0,10	0,27	0,04
STM1200	tmk	0,15	0,00	0,39	0,02
STM1232	purB	0,28	0,03	0,20	0,01
STM1330		1,00	0,10	2,78	0,04
STM1463	add	1,05	0,09	0,99	0,10
STM1707	pyrF	0,97	0,07	0,81	0,03
STM1750	tdk	0,91	0,07	1,46	0,06
STM1756	purU	0,91	0,04	0,57	0,01
STM1780	prsA	0,89	0,05	0,59	0,00
STM1883	purT	0,63	0,05	1,67	0,07
STM2121	dcd	3,75	0,03	0,21	0,01
STM2122	udk	1,61	0,06	1,17	0,09
STM2183	cdd	1,20	0,03	0,72	0,02
STM2187	yeiA	0,64	0,00	0,78	0,02
STM2277	nrdA	1,31	0,05	1,43	0,06
STM2278	nrdB	1,27	0,01	0,97	0,09
STM2362	purF	0,84	0,02	0,69	0,01
STM2409	nupC	1,26	0,03	0,43	0,02
STM2422	xapA	1,11	0,04	0,63	0,02
STM2437	yfeJ	0,85	0,07	0,60	0,03
STM2487	purC	0,59	0,04	1,91	0,01
STM2497	uraA	2,00	0,04	3,49	0,02
STM2498	upp	1,07	0,03	0,77	0,03

STM2499	purM	0,79	0,08	0,56	0,06
STM2500	purN	1,77	0,05	0,26	0,00
STM2502	ppx	0,94	0,07	0,51	0,00
STM2510	guaA	1,03	0,10	0,51	0,04
STM2526	ndk	0,57	0,05	1,48	0,06
STM2565	purG	3,17		0,20	0,01
STM2568	yfhC	1,00	0,08	1,02	0,09
STM2806	nrdI	0,68	0,06	1,56	0,02
STM2808	nrdF	1,90	0,05	0,55	0,05
STM2953	pyrG	0,83	0,03	0,55	0,01
STM3001	thyA	1,04	0,07	0,69	0,01
STM3103	yggV	1,49	0,02	1,33	0,01
STM3167		2,14	0,03	1,59	0,03
STM3333		0,66	0,07	2,77	0,03
STM3334		0,84	0,03	1,29	0,00
STM3631		1,11	0,06	4,75	0,01
STM3731	dut	1,36	0,01	1,02	0,09
STM3733	pyrE	0,94	0,06	34,81	0,00
STM3740	gmk	1,33	0,02	0,72	0,03
STM3747	yicE	0,55	0,08	12,85	0,01
STM3939	cyaA	0,93	0,07	0,68	0,02
STM3968	udp	0,66	0,04	0,82	0,06
STM4104		0,37	0,04	0,77	0,06
STM4160	thiG	1,77	0,01	0,76	0,02
STM4175	purD	2,19	0,02	10,22	0,00
STM4176	purH	0,53	0,06	0,89	0,09
STM4366	purA	1,05	0,08	0,69	0,02
STM4403	cpdB	1,06	0,08	0,86	0,06
STM4452	nrdD	3,47	0,03	0,11	0,01
STM4459	pyrI	0,76	0,01	2,53	0,04
STM4460	pyrB	1,13	0,05	2,12	0,01
STM4471	miaE	0,99	0,08	1,03	0,09
STM4567	deoC	0,62	0,08	0,35	0,01
STM4568	deoA	0,41	0,03	0,27	0,00
STM4570	deoD	0,89	0,01	0,74	0,02

Pseudogenes

PSLT021					
PSLT022					
PSLT058					
PSLT065					
PSLT071					
PSLT109					
STM0241	cutF				
STM0314		0,72	0,06	3,27	0,00
STM0326		1,22	0,08	0,48	0,04

STM0555		0,34	0,01	0,26	0,02
STM0560		0,76	0,02	1,63	0,04
STM0767	dcoA				
STM0790	hutU				
STM1052		1,06	0,10	1,55	0,05
STM1120	ycdF				
STM1186		3,43	0,03	0,14	0,01
STM1464	malY				
STM1465	malX				
STM1474		0,84	0,03	0,82	0,08
STM1553		0,92	0,08	0,71	0,03
STM1666		0,44	0,05	0,69	0,03
STM1866		0,83	0,04	0,79	0,08
STM1930		2,15	0,01	2,28	0,05
STM2003		0,89	0,06	0,64	0,02
STM2664		0,56	0,01	0,51	0,03
STM2778		1,16	0,08	1,68	0,02
STM2975	fucP				
STM3302	yhbE				
STM3460		0,29	0,02	1,35	0,08
STM3530		0,58	0,04	0,76	0,04
STM3654		1,58	0,06	1,45	0,06
STM3806		0,45	0,03	1,03	0,09
STM3844		1,15	0,09	0,29	0,01
STM3945		0,68	0,05	2,09	0,06
STM4111		0,44		0,47	0,01
STM4140					
STM4454	treB				
pSLT					
PSLT001					
PSLT003	repC				
PSLT004	repA3				
PSLT005	tap				
PSLT006	repA				
PSLT008	srgC				
PSLT009	rsK				
PSLT011	srgA				
PSLT012	orf7				
PSLT013	pefI				
PSLT014	orf6				
PSLT015	orf5				
PSLT016	pefD				
PSLT017	pefC				
PSLT018	pefA				
PSLT019	pefB				
PSLT021					

PSLT022	
PSLT023	repA2
PSLT024	
PSLT025	
PSLT026	
PSLT027	ccdA
PSLT028	ccdB
PSLT029	
PSLT030	
PSLT031	rsdB
PSLT032	
PSLT034	
PSLT035	
PSLT036	
PSLT037	spvD
PSLT038	spvC
PSLT039	spvB
PSLT040	spvA
PSLT041	spvR
PSLT042	
PSLT043	
PSLT044	
PSLT045	rlgA
PSLT046	
PSLT047	
PSLT048	tlpA
PSLT049	
PSLT050	
PSLT051	
PSLT052	parA
PSLT053	parB
PSLT054	samB
PSLT055	samA
PSLT056	
PSLT057	
PSLT058	
PSLT059	
PSLT060	
PSLT061	
PSLT062	
PSLT063	
PSLT065	
PSLT066	ssbB
PSLT067	
PSLT068	
PSLT069	psiB

PSLT070	psiA				
PSLT071					
PSLT072					
PSLT073	traM				
PSLT074	finP				
PSLT076	traY				
PSLT077	traA				
PSLT078	traL				
PSLT079	traE				
PSLT080	traK				
PSLT081	traB				
PSLT082	traP				
PSLT083	trbD				
PSLT084	traV				
PSLT085	traR				
PSLT087					
PSLT088	traC				
PSLT089	trbI				
PSLT091	traW				
PSLT092	traU				
PSLT093					
PSLT094	trbC				
PSLT095	traN				
PSLT096	trbE				
PSLT097	traF				
PSLT098	traQ				
PSLT099	trbB				
PSLT100	traH				
PSLT101	traG				
PSLT102	traS				
PSLT103	traT				
PSLT104	traD				
PSLT105	trbH				
PSLT106					
PSLT107					
PSLT109					
PSLT110	traX				
PSLT111	finO				

**Signal transduction**					
STM2876	hilA	0,09	0,00	0,26	0,00
SPI1 to SPI5					
STM0053		1,67	0,01	1,54	0,05
STM0186	dksA	1,11	0,08	1,02	0,10
STM0343		2,12	0,03	1,17	0,08
STM0367	prpR	1,12	0,07	1,26	0,04

STM0385	yaiC	1,29	0,00	0,44	0,04
STM0397	phoB	2,91	0,03	0,14	0,01
STM0398	phoR	0,65	0,05	0,41	0,01
STM0446	bolA	0,95	0,03	0,57	0,01
STM0468	ylaB	2,93	0,02	2,59	0,03
STM0549	fimZ	0,79	0,07	0,96	0,09
STM0561		1,04	0,07	1,00	0,10
STM0600	cstA	0,93	0,06	0,88	0,01
STM0614	ybdQ	0,90	0,01	0,79	0,06
STM0625	dpiB	1,35	0,05	0,65	0,02
STM0669	phoL	1,14	0,02	0,83	0,06
STM0702	kdpE	4,09	0,02	0,23	0,02
STM0703	kdpD	0,68	0,06	1,25	0,09
STM0825	ybiI	0,23	0,01	0,80	0,05
STM1087	pipA	0,74	0,03	0,71	0,06
STM1088	pipB	6,91	0,00	0,66	0,03
STM1089		1,05	0,08	1,71	0,02
STM1090	pipC	1,25	0,06	1,29	0,08
STM1091	sopB	0,63	0,04	0,50	0,00
STM1092	orfX	0,52	0,03	0,40	0,06
STM1093					
STM1094	pipD	1,02	0,09	0,56	0,03
STM1095	copS	0,17	0,00	6,26	0,02
STM1096	copR	0,60	0,06	1,18	0,09
STM1126	phoH	2,64	0,02	0,12	0,00
STM1230	phoQ	3,45	0,04	4,95	0,02
STM1231	phoP	3,89	0,00	1,37	0,06
STM1283	yeaJ	0,71	0,05	3,08	0,01
STM1285	yeaG	1,27	0,03	0,45	0,00
STM1344	ydiV	1,09	0,05	1,08	0,07
STM1379	orf48	1,23	0,02	1,04	0,10
STM1380	orf32	1,17	0,05	2,12	0,05
STM1381	orf245	1,08	0,09	1,16	0,09
STM1382	orf408	0,94	0,08	1,61	0,00
STM1383	ttrA	1,51	0,02	1,04	0,07
STM1384	ttrC	0,86	0,03	2,67	0,05
STM1386	ttrS	0,28	0,04	0,86	0,08
STM1386	ttrS	0,28	0,04	0,86	0,08
STM1387	ttrR	3,25	0,03	1,98	0,05
STM1387	ttrR	3,25	0,03	1,98	0,05
STM1388	orf70	0,39	0,03	0,50	0,01
STM1389	orf319	1,29	0,07	0,64	0,01
STM1390	orf242	0,84	0,04	0,41	0,02
STM1391	ssrB	0,99	0,09	0,59	0,02
STM1391	ssrB	0,99	0,09	0,59	0,02
STM1392	ssrA	0,70	0,04	0,65	0,03

STM1392	ssrA	0,70	0,04	0,65	0,03
STM1393	ssaB	1,09	0,09	0,15	0,01
STM1394	ssaC	1,31	0,06	1,90	0,06
STM1396	ssaE	1,01	0,10	0,95	0,08
STM1397	sseA	0,85	0,06	0,88	0,09
STM1398	sseB	1,31	0,06	0,94	0,09
STM1399	sscA	3,40	0,02	0,22	0,02
STM1400	sseC	1,30	0,03	0,84	0,09
STM1401	sseD	1,50	0,01	0,74	0,04
STM1402	sseE	1,11	0,06	0,55	0,02
STM1403	sscB	0,84	0,05	6,97	0,00
STM1404	sseF	0,66	0,03	1,89	0,06
STM1405	sseG	0,27	0,01	0,77	0,08
STM1406	ssaG	3,55	0,02	0,13	0,02
STM1407	ssaH	0,91	0,09	0,78	0,09
STM1408	ssaI	1,03	0,09	0,95	0,10
STM1409	ssaJ	1,51	0,04	0,71	0,08
STM1411	ssaK	0,23	0,04	0,49	0,03
STM1412	ssaL	1,54	0,01	0,52	0,05
STM1413	ssaM	0,20	0,01	0,20	
STM1414	ssaV	0,32	0,04	0,28	0,00
STM1415	ssaN	0,51	0,00	0,55	0,02
STM1416	ssaO	1,04	0,08	0,47	0,06
STM1417	ssaP	2,29	0,05	3,99	0,02
STM1419	ssaR	1,05	0,09	0,50	0,03
STM1420	ssaS	1,70	0,06	0,28	0,00
STM1421	ssaT	0,59	0,07	0,36	0,01
STM1422	ssaU	0,35	0,00	4,29	0,02
STM1471	rstB	1,09	0,09	1,50	0,05
STM1475	rstA	1,53	0,02	1,14	0,09
STM1652	ynaF	0,86	0,06	0,87	0,09
STM1660	fnr	0,99	0,10	0,39	0,03
STM1661	ydaA	0,34	0,03	0,77	0,08
STM1691	pspF				
STM1697		0,48	0,02	1,33	0,07
STM1703	yciR	1,82	0,05	0,90	0,07
STM1753	hnr	0,74	0,03	0,77	0,04
STM1766	narX	0,59	0,04	1,12	0,07
STM1767	narL	1,26	0,02	0,44	0,01
STM1827		0,73	0,08	0,81	0,05
STM1846	proQ	1,10	0,07	0,91	0,09
STM1847	yebR	1,11	0,05	0,77	0,04
STM1853	prpA	0,69	0,06	1,99	0,05
STM1916	cheY	1,57	0,07	10,57	0,00
STM1927	yecG	4,24	0,02	0,13	0,00
STM1947	uvrY	0,88	0,09	1,18	0,09

STM1987		1,29	0,04	0,88	0,09
STM2117	wzb	1,11	0,08	4,78	0,00
STM2130	baeS	0,87	0,02	1,10	0,08
STM2131	baeR	1,37	0,01	0,43	0,00
STM2159	yehU	1,25	0,02	1,39	0,07
STM2215	rtn	1,13	0,09	0,86	0,09
STM2246	narP	0,87	0,03	0,75	0,03
STM2269	yojN	1,15	0,01	1,16	0,06
STM2270	rcsB	3,75	0,02	0,12	0,01
STM2271	rcsC	1,12	0,08	1,21	0,07
STM2314		2,19	0,01	0,96	0,09
STM2387	sixA	1,44	0,03	1,17	0,08
STM2396	pgtA	0,29	0,00	0,56	0,01
STM2397	pgtB	0,94	0,10	6,64	0,00
STM2410	yfeA	0,93	0,06	2,47	0,05
STM2480	narQ	0,53	0,05	1,87	0,04
STM2503		0,70	0,06	0,88	0,08
STM2562	yfhA	0,90	0,04	0,68	0,00
STM2564	yfhK	1,24	0,03	1,98	0,03
STM2637	rseC	0,40	0,00	19,60	0,00
STM2638	rseB	1,70	0,00	0,73	0,04
STM2639	rseA	1,42	0,00	1,71	0,07
STM2672	yfiN	0,93	0,04	1,29	0,03
STM2731		0,67	0,01	1,33	0,06
STM2784	tctE	0,30	0,01	1,03	0,10
STM2785	tctD	0,54	0,04	5,00	0,03
STM2817	luxS	1,05	0,08	0,51	0,01
STM2826	csrA	0,88	0,01	0,73	0,07
STM2865	avrA	1,06	0,05	1,04	0,09
STM2866	sprB	0,98	0,08	1,79	0,06
STM2867	hilC	0,02	0,00	1,28	0,08
STM2868		1,14	0,07	1,14	0,07
STM2869	orgA	0,64	0,07	0,52	0,03
STM2871	prgK	2,64	0,03	0,53	0,01
STM2872	prgJ	10,67	0,05	0,19	0,01
STM2873	prgI	1,02		0,32	0,07
STM2874	prgH	1,41	0,02	0,67	0,02
STM2875	hilD	0,63	0,01	1,38	0,06
STM2877	iagB	3,25	0,03	0,19	0,01
STM2878	sptP	1,48	0,05	0,70	0,04
STM2879	sicP	0,88	0,05	0,73	0,07
STM2880		1,58	0,03	1,60	0,06
STM2881	iacP	0,96	0,08	1,78	0,02
STM2882	sipA	0,94	0,08	0,74	0,02
STM2883	sipD	0,78	0,03	1,12	0,07
STM2884	sipC	3,10	0,02	0,21	0,00

STM2885	sipB	0,10			
STM2886	sicA	1,04	0,08	0,81	0,05
STM2887	spaS	0,87	0,07	1,63	0,04
STM2888	spaR	0,77	0,02	2,24	0,05
STM2889	spaQ	2,55	0,00	2,02	0,04
STM2890	spaP	1,15	0,03	0,64	0,02
STM2892	invJ	1,00	0,10	0,94	0,08
STM2893	invI	0,82	0,04	1,35	0,04
STM2894	invC	0,94	0,05	2,27	0,05
STM2895	invB	0,97	0,09	0,70	0,03
STM2896	invA	0,52	0,05	0,66	0,01
STM2897	invE	1,07	0,08	1,23	0,05
STM2899	invF	3,92	0,05	1,45	0,01
STM2900	invH	1,02	0,08	0,47	0,01
STM2901		1,28	0,03	1,14	0,08
STM2902		1,45	0,06	0,85	0,02
STM2903		0,48		0,41	0,05
STM2904		0,75	0,06	0,54	0,04
STM2905		0,65	0,05	1,05	0,09
STM2906				7,81	
STM2907	pphB	0,63	0,04	1,79	0,07
STM2907	pphB	0,63	0,04	1,79	0,07
STM2908		0,66	0,07	1,38	0,07
STM2909	mutS			0,25	0,03
STM2911		1,09	0,08	1,19	0,08
STM2912		3,50	0,02	0,13	0,01
STM2913		0,65	0,05	2,34	0,05
STM2914		0,34	0,05	1,15	0,09
STM2956	relA	1,62	0,02	1,26	0,08
STM2958	barA	0,79	0,02	0,99	0,09
STM3003	ptsP	0,95	0,07	0,67	0,02
STM3177	ygiX				
STM3178	ygiY				
STM3203	ygiM	3,67	0,00	0,91	0,09
STM3217	aer	0,79	0,03	0,52	0,00
STM3328	arcB	3,29	0,04	0,30	0,01
STM3375	yhdA	1,25	0,03	1,13	0,05
STM3388		0,99	0,09	0,82	0,05
STM3466	crp	1,16	0,08	1,02	0,09
STM3501	envZ	1,98		7,95	
STM3502	ompR	0,65	0,04	0,99	0,10
STM3522	rtcR	0,64	0,05	0,19	0,00
STM3591	uspA	1,48	0,06	0,71	0,07
STM3606	yhjB	0,64	0,01	1,08	0,07
STM3611	yhjH	1,02	0,10	1,52	0,07
STM3615	yhjK	0,99	0,08	1,61	0,03

STM3742	spoT	0,58	0,04	1,30	0,02
STM3752		0,94	0,09	1,13	0,09
STM3753	sugR	0,89	0,03	0,89	0,03
STM3755	rhuM	2,35	0,03	0,48	0,05
STM3756	rmbA	0,98	0,09	1,23	0,06
STM3757	misL				
STM3758	fidL	1,09	0,09	1,65	0,05
STM3759	marT	1,81	0,06	0,75	0,07
STM3761	slsA	0,18	0,00	5,56	0,02
STM3762	cigR	1,34	0,02	0,66	0,00
STM3763	mgtB	1,04	0,08	1,02	0,09
STM3764	mgtC	0,77	0,05	2,81	0,04
STM3766		0,90	0,08	6,47	0,00
STM3767		0,71	0,01	1,66	0,06
STM3768		0,98	0,10	1,21	0,08
STM3769		0,57	0,03	1,49	0,08
STM3770		3,38	0,02	0,13	0,01
STM3771					
STM3772		0,27		4,21	0,05
STM3773		1,54	0,06	0,91	0,02
STM3774		0,86	0,07	1,34	0,07
STM3775		4,39	0,03	0,38	0,03
STM3779		0,72	0,00	1,48	0,07
STM3780		0,92	0,08	1,44	0,07
STM3781		0,65	0,06	1,31	0,08
STM3782		0,85	0,09	0,70	0,07
STM3783		0,16	0,03	10,40	0,00
STM3784		1,92	0,05	0,30	0,02
STM3789	uhpB	0,07	0,02	0,92	0,07
STM3790	uhpA	0,91	0,07	0,74	0,03
STM3824	torR	1,04	0,04	0,41	0,05
STM3826	torS	3,13	0,04	0,86	0,09
STM4005	glnG	0,68	0,02	10,34	0,00
STM4009	typA	1,25	0,02	0,66	0,01
STM4058	cpxA	0,67	0,05	1,19	0,07
STM4059	cpxR				
STM4173	hydH	0,50	0,01	0,93	0,08
STM4174	hydG	0,76	0,03	1,52	0,01
STM4185	aceK	0,81	0,08	1,11	0,09
STM4257		0,73	0,01	1,50	0,05
STM4258		0,83	0,02	1,13	0,06
STM4259		0,99	0,09	0,55	0,02
STM4260		1,12	0,03	2,97	0,04
STM4261		5,35	0,04	1,12	0,05
STM4262		0,75	0,02	1,90	0,03
STM4264	yjcC	0,84	0,05	1,72	0,01

STM4291	basS	0,67	0,02	3,49	0,01
STM4292	basR	1,36	0,03	0,68	0,00
STM4304	dcuS	0,24	0,03	0,68	0,05
STM4532	yjiY	0,65	0,06	0,89	0,08
STM4534		0,94	0,05	0,72	0,03
STM4547	yjjQ	0,85	0,02	1,46	0,07
STM4548	bglJ	0,27	0,03	1,33	0,08
STM4551		0,74	0,04	0,57	0,02
STM4588	creB	3,26	0,04	1,13	0,07
STM4589	creC	1,22	0,08	1,29	0,07
STM4598	arcA	0,76	0,03	1,43	0,06

Surface structure

STM0319	crl	1,20	0,08	0,46	0,01
STM0543	fimA	1,05	0,02	0,66	0,01
STM0544	fimI	8,45	0,01	1,14	0,09
STM0545	fimC			0,57	0,05
STM0548	fimF	0,96	0,08	0,70	0,02
STM0549	fimZ	0,79	0,07	0,96	0,09
STM1139	csgG	0,81	0,06	1,44	0,05
STM1140	csgF	0,84	0,02	0,68	0,03
STM1141	csgE	0,85	0,04	2,51	0,05
STM1143	csgB	0,77	0,04	1,42	0,07
STM1144	csgA	0,26	0,02	0,28	0,01
STM1171	flgN	0,10	0,00	4,35	0,01
STM1172	flgM	0,12	0,05	2,99	0,00
STM1173	flgA	2,46	0,04	0,65	0,03
STM1174	flgB	20,72		0,53	0,02
STM1175	flgC	1,13	0,04	0,68	0,03
STM1176	flgD	0,76	0,01	1,62	0,03
STM1177	flgE	0,86	0,02	3,33	0,01
STM1178	flgF	1,13	0,04	0,62	0,02
STM1179	flgG	0,88	0,04	2,55	0,05
STM1180	flgH	4,02	0,02	1,79	0,06
STM1181	flgI	1,10	0,04	0,66	0,02
STM1182	flgJ	0,84	0,02	6,84	0,02
STM1183	flgK	0,79	0,02	0,61	0,00
STM1184	flgL	0,94	0,10	0,58	0,06
STM1807	dsbB	0,90	0,02	0,68	0,02
STM1924	flhC	0,86	0,07	0,61	0,06
STM1925	flhD	1,01	0,10	1,02	0,10
STM1956	fliA	1,43	0,06	2,33	0,00
STM1959	fliC	0,74	0,01	0,72	0,03
STM1960	fliD	0,79	0,04	0,62	0,07
STM1961	fliS	1,61	0,06	2,04	0,03
STM1962	fliT	0,64	0,02	1,73	0,01

STM1968	fliE			2,37	0,00
STM1969	fliF			0,39	0,00
STM1970	fliG	0,84	0,03	7,18	0,02
STM1971	fliH	0,54	0,00	0,49	0,01
STM1972	fliI	1,00	0,10	2,06	0,00
STM1973	fliJ	0,96	0,09	1,43	0,04
STM1974	fliK	1,04	0,08	0,69	0,03
STM1975	fliL	0,93	0,04	1,02	0,09
STM1976	fliM	0,30	0,00	3,29	0,01
STM1977	fliN	2,05	0,05	1,68	0,03
STM1978	fliO	1,74	0,05	0,34	0,02
STM1979	fliP	0,50	0,04	3,09	0,03
STM1980	fliQ	0,50	0,02	0,97	0,09
STM1981	fliR	1,10	0,08	1,17	0,09
STM3043	dsbC	0,91	0,05	0,57	0,00

Translation, ribosomal structure and biogenesis

STM0043	rpsT	1,34	0,01	0,50	0,03
STM0046	ileS	3,74	0,02	0,13	0,00
STM0090	ksgA	3,95	0,02	0,24	0,01
STM0095	rluA	0,09	0,02	1,42	0,02
STM0185	yadB	1,98	0,05	1,30	0,06
STM0188	ligT	1,19	0,03	3,23	0,00
STM0215	map	1,07	0,05	0,66	0,01
STM0216	rpsB	1,47	0,00	0,61	0,02
STM0217	tsf	2,90	0,03	0,13	0,01
STM0219	frr	1,15	0,01	0,94	0,06
STM0240	yaeJ	0,38	0,05	1,32	0,03
STM0242	proS	1,38	0,00	0,35	0,00
STM0315	prfH	0,63	0,02	1,15	0,08
STM0404	queA	1,27	0,07	0,77	0,08
STM0469	rpmE2	0,62	0,04	4,21	0,02
STM0470	rpmJ2	3,18	0,03	0,14	0,01
STM0537	cysS	0,86	0,04	0,65	0,00
STM0617	rna	1,00	0,10	0,62	0,02
STM0648	leuS	0,73	0,07	1,15	0,09
STM0670	miaB	1,13	0,03	1,30	0,05
STM0686	glnS	0,85	0,03	0,76	0,00
STM0852	yliG	3,45	0,03	0,38	0,01
STM0882	ybjF	0,55	0,07	3,07	0,02
STM0953	infA	1,51	0,06	1,11	0,09
STM0963	serS	1,22	0,08	0,67	0,07
STM0981	rpsA	0,98	0,10	1,00	0,10
STM1000	asnS	1,00	0,10	0,47	0,00
STM1066	rmf	1,25	0,01	0,83	0,05
STM1167	rimJ	0,67	0,06	0,49	0,02

STM1185	rne	0,91	0,04	0,69	0,02
STM1187	rluC	2,41	0,00	0,92	0,09
STM1191	rpmF	0,82	0,03	0,73	0,00
STM1234	trmU	2,79	0,02	0,19	0,03
STM1237	ymfC	0,81	0,02	1,24	0,07
STM1333	thrS	1,00	0,10	0,64	0,02
STM1334	infC				
STM1335	rpmI	1,31	0,02	0,51	0,02
STM1336	rplT	1,10	0,03	0,55	0,02
STM1337	pheS	0,93	0,06	0,54	0,03
STM1449	tyrS	0,85	0,09	0,83	0,09
STM1502	speG	1,21	0,05	1,36	0,08
STM1548		0,71	0,02	0,75	0,04
STM1549		1,43	0,07	2,76	0,00
STM1550		0,76	0,08	1,14	0,08
STM1611	rimL	0,80	0,03	0,75	0,01
STM1706	yciH	0,99	0,09	0,49	0,01
STM1719	yciL	0,84	0,03	1,14	0,05
STM1720	yciO	1,09	0,08	0,55	0,00
STM1775	hemK	0,89	0,06	0,64	0,01
STM1776	prfA	0,36	0,04	1,13	0,06
STM1783	pth	0,80	0,00	1,26	0,04
STM1784	ychF	0,86	0,05	0,80	0,05
STM1817	rnd	0,64	0,07	0,54	0,05
STM1822	yoaB	0,98	0,08	0,89	0,07
STM1850	yebU	0,85	0,08	1,05	0,09
STM1901	aspS	0,74	0,07	0,70	0,06
STM1909	argS	1,14	0,03	0,57	0,00
STM2155	metG	1,09	0,07	0,78	0,02
STM2174	yohI	0,27	0,02	0,86	0,06
STM2211	yeiP	1,19	0,01	1,42	0,04
STM2222	rsuA	1,37	0,00	1,09	0,08
STM2224	rplY	0,15	0,01	0,60	0,01
STM2368	truA	0,98	0,09	1,45	0,07
STM2385	yfcB	0,84	0,02	0,59	0,00
STM2415	gltX	1,24	0,04	1,17	0,08
STM2545		0,98	0,06	0,91	0,08
STM2648	yfiF	1,14	0,03	0,47	0,02
STM2662	rluD	0,92	0,06	0,81	0,04
STM2665	yfiA	0,57	0,00	0,86	0,05
STM2674	trmD	0,88	0,03	0,64	0,01
STM2675	rimM	1,14	0,02	0,84	0,04
STM2676	rpsP	1,40	0,06	0,93	0,09
STM2685	smpA	1,05	0,08	0,59	0,02
STM2827	alaS	0,67	0,07	0,93	0,09
STM2957	ygcA	1,07	0,06	0,82	0,06

STM2964	yqcB	1,24	0,09	1,05	0,10
STM3040	lysS	1,22	0,00	0,74	0,03
STM3041	prfB	0,68	0,02	1,96	0,05
STM3204	cca	0,58	0,07	2,18	0,05
STM3209	rpsU	1,44	0,06	1,09	0,09
STM3220	ygjO	0,84	0,07	0,97	0,09
STM3282	pnp	0,97	0,06	0,75	0,02
STM3283	rpsO	1,03	0,08	0,88	0,07
STM3284	truB	1,19	0,03	1,31	0,07
STM3285	rbfA	0,79	0,03	0,95	0,08
STM3286	infB	1,18	0,01	0,75	0,01
STM3297	ftsJ	1,05	0,06	0,93	0,09
STM3298	yhbY	0,81	0,00	0,77	0,05
STM3303	rpmA	1,18	0,04	0,52	0,02
STM3304	rplU	1,57	0,03	1,79	0,06
STM3321	yhbH	1,00	0,10	0,78	0,08
STM3344	rpsI	1,26	0,01	0,82	0,05
STM3345	rplM	1,23	0,02	0,54	0,03
STM3370	cafA	0,98	0,09	0,70	0,04
STM3383	prmA	0,89	0,06	0,64	0,02
STM3384	yhdG	0,98	0,05	0,81	0,05
STM3402	yrdC	1,06	0,07	0,59	0,01
STM3406	def	0,98	0,09	0,81	0,08
STM3407	fmt	1,47	0,05	0,74	0,07
STM3408	sun	3,11	0,03	0,12	0,01
STM3414	rplQ	1,04	0,06	0,55	0,00
STM3416	rpsD	1,47	0,00	0,66	0,03
STM3417	rpsK	1,11	0,06	0,64	0,01
STM3418	rpsM	1,84	0,01	1,34	0,08
STM3419	rpmJ	1,13	0,03	0,57	0,02
STM3421	rplO	1,33	0,03	1,07	0,09
STM3422	rpmD	0,88	0,01	0,54	0,00
STM3423	rpsE	1,41	0,00	1,42	0,08
STM3424	rplR	0,99	0,10	0,81	0,05
STM3425	rplF	1,11	0,07	0,59	0,00
STM3426	rpsH	1,61	0,02	1,16	0,09
STM3427	rpsN	0,99	0,09	0,58	0,01
STM3428	rplE	1,06	0,03	0,67	0,01
STM3429	rplX	0,93	0,06	0,52	0,00
STM3430	rplN	0,92	0,07	0,61	0,01
STM3431	rpsQ	1,04	0,09	1,05	0,10
STM3432	rpmC	0,99	0,10	0,75	0,01
STM3433	rplP	0,79	0,03	0,63	0,01
STM3434	rpsC	1,00	0,10	0,41	0,00
STM3435	rplV	1,20	0,04	0,99	0,10
STM3436	rpsS	0,97	0,09	0,77	0,02

STM3437	rplB	0,79	0,04	0,65	0,01
STM3438	rplW	0,63	0,03	0,70	0,01
STM3439	rplD	1,05	0,08	0,50	0,01
STM3440	rplC	1,16	0,03	1,04	0,10
STM3441	rpsJ	1,00	0,10	0,74	0,03
STM3445	tufA	1,24	0,08	0,65	0,06
STM3446	fusA	1,80	0,02	1,42	0,07
STM3447	rpsG	0,77	0,04	0,64	0,03
STM3448	rpsL	1,21	0,00	0,84	0,04
STM3481	trpS	1,18	0,04	0,98	0,10
STM3497	yrfH	1,20	0,05	0,69	0,01
STM3655	glyS	0,96	0,08	1,18	0,07
STM3656	glyQ	0,92	0,03	0,88	0,01
STM3682	selB	1,00	0,10	0,69	0,01
STM3695	yibK	3,27	0,03	1,05	0,10
STM3727	rpmG	0,59	0,04	0,93	0,08
STM3728	rpmB	1,46	0,06	1,33	0,08
STM3734	rph	0,81	0,02	0,62	0,01
STM3743	spoU	0,93	0,08	0,88	0,02
STM3839	rpmH	2,13	0,02	0,72	
STM3840	rnpA	1,73	0,01	2,66	0,01
STM4028	yihZ	0,89	0,04	0,66	0,02
STM4096	rpmE	1,12	0,05	1,40	0,08
STM4129	trmA	0,95	0,05	0,89	0,07
STM4146	tufB	0,59		0,34	
STM4149	rplK	1,06	0,02	0,83	0,05
STM4150	rplA	0,84	0,06	0,41	0,00
STM4151	rplJ	0,63	0,06	0,93	0,10
STM4152	rplL	0,64	0,03	0,88	0,09
STM4193	yjbC	0,92	0,06	0,54	0,00
STM4243	yjbN	0,98	0,09	0,63	0,03
STM4334	efp	1,03	0,10	0,79	0,08
STM4344	yjeA	1,08	0,05	0,94	0,06
STM4360	miaA	0,77	0,02	0,87	0,08
STM4369	yjfH	1,48	0,03	2,44	0,02
STM4391	rpsF	1,12	0,03	0,84	0,06
STM4393	rpsR	1,03	0,09	0,60	0,01
STM4394	rplI	0,95	0,07	0,85	0,07
STM4450		0,54	0,00	0,93	0,09
STM4458	yjgF	1,03	0,03	0,90	0,04
STM4508	trpS2	0,63	0,02	0,63	0,01
STM4556	rsmC	0,70	0,04	2,96	0,02
STM4560	prfC	1,24	0,04	8,14	0,00
STM4600	lasT	0,22	0,03	0,71	0,07

Transcription

STM0014		1,07	0,06	0,64	0,02
STM0017		1,00	0,10	1,56	0,04
STM0029		0,98	0,09	1,38	0,05
STM0030		1,07	0,08	1,14	0,06
STM0031		1,08	0,05	0,61	0,02
STM0040	nhaR	1,32	0,01	0,60	0,00
STM0052		0,49	0,03	5,23	0,00
STM0104	araC	0,97	0,07	1,61	0,06
STM0115	leuO	0,69	0,01	1,36	0,05
STM0118	fruR	0,60	0,01	0,80	0,00
STM0151	pdhR	1,00	0,10	0,55	0,00
STM0164		1,01	0,10	0,49	0,01
STM0210	cdaR	1,31	0,08	1,39	0,08
STM0256	yafC	0,94	0,09	0,62	0,06
STM0304	sinR	0,91	0,03	2,81	0,04
STM0333		0,28	0,03	0,31	0,02
STM0347		0,67	0,08	0,47	0,04
STM0354		0,35	0,02	1,11	0,06
STM0363		1,56	0,05	1,06	0,09
STM0410		1,33	0,07	1,28	0,07
STM0418	nusB	1,26	0,07	0,61	0,06
STM0430	phnR	1,42	0,03	0,68	0,04
STM0459	ybaO	0,79	0,08	0,42	0,04
STM0477	acrR	0,74	0,07	1,30	0,09
STM0499	cueR	1,02	0,08	0,74	0,00
STM0514	ybbS	1,23	0,09	0,94	0,09
STM0516	allR	1,84	0,01	1,39	0,07
STM0552	fimW	0,73	0,03	1,23	0,05
STM0571		1,09	0,07	1,27	0,08
STM0580		1,45	0,03	0,98	0,09
STM0581		0,32	0,02	0,52	0,02
STM0604	ybdM	0,65	0,02	0,75	0,01
STM0606	ybdO	0,76	0,01	1,02	0,10
STM0616	rnk	1,24	0,01	1,49	0,06
STM0626	dpiA	1,18	0,03	3,33	0,02
STM0629	cspE	1,16	0,02	0,60	0,00
STM0634	ybeF	1,45	0,01	2,98	0,03
STM0652		0,99	0,10	0,30	0,01
STM0682	nagC	0,71	0,01	0,65	0,00
STM0692		1,00	0,10	1,15	0,08
STM0763		1,00	0,10	0,20	0,00
STM0764		1,03	0,08	0,56	0,02
STM0789	hutC	1,49	0,01	1,04	0,04
STM0819	ybiH	1,12	0,02	1,08	0,08
STM0821	dinG	0,12	0,02	0,91	0,06
STM0835		0,63	0,05	0,53	0,01

STM0859		4,04	0,01	1,04	0,10
STM0898					
STM0900					
STM0943	cspD	1,12	0,05	0,71	0,04
STM0952		4,79	0,00	0,41	0,04
STM0959	lrp	1,59	0,03	0,36	0,00
STM1001		0,54	0,06	18,49	0,00
STM1012		1,04	0,06	0,78	0,06
STM1072	yccR	0,91	0,09	2,33	0,04
STM1082		0,72	0,01	0,48	0,02
STM1100	hpaR	0,66	0,05	2,86	0,04
STM1108	hpaA	1,05	0,07	0,61	0,03
STM1122	ycdC	0,99	0,10	1,14	0,09
STM1127		0,95	0,09	0,84	0,08
STM1142	csgD	1,09	0,07	2,66	0,01
STM1172	flgM	0,12	0,05	2,99	0,00
STM1213	ycfQ	1,76	0,05	0,83	0,08
STM1220	ycfX	0,77	0,00	0,87	0,01
STM1221	cobB	1,15	0,06	0,73	0,02
STM1243		1,85	0,01	1,26	0,03
STM1265		3,06	0,00	0,94	0,09
STM1279	yeaM	0,54	0,04	0,84	0,08
STM1315	celD	0,98	0,08	7,52	0,02
STM1355	ydiP	0,85	0,03	2,66	0,05
STM1382	orf408	0,94	0,08	1,61	0,00
STM1390	orf242	0,84	0,04	0,41	0,02
STM1429	ydhB	0,65	0,05	0,62	0,06
STM1430	purR	0,48	0,05	1,25	0,08
STM1437	ydhM	0,95	0,04	0,72	0,03
STM1444	slyA	0,97	0,10	1,01	0,10
STM1487	ynfL	0,76	0,07	6,20	0,01
STM1488	mlc	1,08	0,09	1,49	0,07
STM1510	ydfH	1,20	0,03	0,71	0,02
STM1519	marA	0,97	0,10	5,76	0,03
STM1520	marR	5,15	0,03	0,78	0,08
STM1523	yneJ	1,06	0,08	0,59	0,01
STM1541		18,59	0,00	0,79	0,08
STM1547		10,08	0,04	0,21	0,00
STM1555		1,04	0,09	4,14	0,02
STM1575		1,59	0,01	2,10	0,01
STM1588	yncC	0,74	0,08	0,97	0,10
STM1598	ydcR	0,83	0,09	0,54	0,05
STM1605	ydcN	1,12	0,09	5,37	0,01
STM1618		1,61	0,02	1,46	0,06
STM1619		1,32	0,04	2,29	0,00
STM1625	ydcI	1,67	0,05	10,19	0,02

STM1664		1,58	0,00	0,79	0,06
STM1671		2,80	0,03	0,15	0,01
STM1674		0,28	0,01	2,02	0,01
STM1677		1,45	0,07	0,93	0,09
STM1688	pspC				
STM1690	pspA				
STM1704	yciT	1,30	0,02	0,77	0,05
STM1713	cysB	1,07	0,06	1,76	0,06
STM1805	fadR	1,09	0,07	0,99	0,10
STM1821	yoaA	0,48	0,01	0,63	0,03
STM1837	cspC	1,42	0,00	0,62	0,03
STM1842	kdgR	3,70	0,03	0,12	0,01
STM1857		1,25	0,02	0,63	0,06
STM1887	yebK	3,76	0,02	0,22	0,02
STM1950	sdiA	3,60	0,02	0,15	0,01
STM1956	fliA	1,43	0,06	2,33	0,00
STM1982	rcsA	2,32	0,00	2,07	0,04
STM1996	cspB	9,31	0,01	0,32	0,04
STM1998	umuD	1,46	0,02	1,27	0,08
STM2145	yegW	1,12	0,07	0,70	0,06
STM2158	yehT	2,19	0,04	0,53	0,03
STM2160	yehV	3,57	0,03	0,14	0,01
STM2180		1,80	0,04	0,67	0,03
STM2191	galS	1,17	0,08	2,36	0,02
STM2201	yeiE	1,02	0,07	0,67	0,00
STM2223	yejH	0,84	0,02	0,93	0,05
STM2230		0,89	0,06	1,62	0,04
STM2275		0,76	0,01	1,94	0,04
STM2281		0,37	0,02	1,74	0,06
STM2292	yfaX	0,53	0,04	0,88	0,06
STM2330	lrhA	0,92	0,06	0,69	0,03
STM2345		0,80	0,04	0,49	0,01
STM2361		1,09	0,05	1,73	0,05
STM2374		1,40	0,02	0,45	0,01
STM2420	xapR	1,05	0,08	1,18	0,06
STM2424	yfeR	0,99	0,10	1,47	0,05
STM2436	ptsJ	0,83	0,05	0,67	0,02
STM2454	eutR	0,42	0,04	0,71	0,07
STM2544	yfhP	1,99	0,02	1,28	0,08
STM2553	csiE	0,87	0,08	1,39	0,08
STM2557	cadC	0,86	0,03	0,53	0,00
STM2572	yfhH	0,30	0,02	0,84	0,08
STM2575		2,27	0,00	2,08	0,04
STM2581	rnc	0,91	0,05	0,78	0,05
STM2640	rpoE	3,84	0,02	0,13	0,01
STM2644	yfiE	2,17	0,01	0,98	0,09

STM2738					
STM2744				0,10	0,01
STM2748		2,46	0,02	0,47	0,00
STM2749		0,81	0,01	1,50	0,06
STM2794	ygaE	0,61	0,06	3,50	0,01
STM2797		0,98	0,09	1,18	0,08
STM2803		0,48	0,01	0,63	0,00
STM2813	emrR	1,05	0,08	0,51	0,02
STM2837	srlR	0,91	0,06	0,91	0,07
STM2839	ygaA	0,17		0,63	
STM2859	fhlA	1,12	0,04	0,77	0,01
STM2866	sprB	0,98	0,08	1,79	0,06
STM2867	hilC	0,02	0,00	1,28	0,08
STM2875	hilD	0,63	0,01	1,38	0,06
STM2876	hilA	0,09	0,00	0,26	0,00
STM2899	invF	3,92	0,05	1,45	0,01
STM2905		0,65	0,05	1,05	0,09
STM2912		3,50	0,02	0,13	0,01
STM2919	ygbI	0,88	0,09	2,92	0,01
STM2920		0,61	0,04	0,40	0,00
STM2924	rpoS	2,38	0,02	0,28	0,03
STM2955		0,88	0,03	0,78	0,07
STM2979	fucR	0,95	0,08	0,91	0,08
STM2982	gcvA	0,95	0,07	0,74	0,04
STM3011	galR	0,90	0,03	0,82	0,03
STM3012		0,74	0,02	0,71	0,01
STM3014	lysR	4,86	0,03	0,29	0,03
STM3020		1,65	0,00	1,30	0,02
STM3025		0,51	0,03	0,31	0,02
STM3064	iciA	13,89	0,01	1,29	0,09
STM3084		1,02	0,09	0,68	0,02
STM3096	yqgE	1,56	0,06	1,96	0,06
STM3098		0,29	0,03	5,13	0,00
STM3121		0,92	0,07	0,73	0,02
STM3124		1,03	0,10	1,42	0,07
STM3163	yqhC	2,98	0,03	0,20	0,02
STM3175		1,23	0,09	0,96	0,09
STM3211	rpoD	0,73	0,01	0,63	0,02
STM3215	yqjI	0,85	0,07	0,85	0,06
STM3235	yhaJ	0,69	0,07	2,48	0,02
STM3245	tdcA	1,56	0,03	3,49	0,00
STM3252	agaR	0,59	0,03	1,11	0,09
STM3262		1,35	0,07	2,78	0,02
STM3287	nusA	0,80	0,08	1,16	0,09
STM3299	greA	1,08	0,03	0,73	0,04
STM3306	nlp	1,10	0,08	0,81	0,03

STM3320	rpoN	1,18	0,05	1,37	0,07
STM3336	nanK	0,67	0,04	1,21	0,04
STM3340	yhcK	1,03	0,08	0,97	0,09
STM3357		1,02	0,09	0,52	0,02
STM3358		1,21	0,02	1,68	0,00
STM3363	yhcO	0,89	0,05	0,68	0,03
STM3367	yhcS				
STM3385	fis	0,34	0,02	0,69	0,02
STM3389	envR	0,34	0,00	1,58	0,04
STM3412	zntR	0,94	0,07	0,73	0,04
STM3415	rpoA	1,50	0,00	0,78	0,03
STM3503	greB	1,39	0,02	0,90	0,06
STM3515	malT	1,16	0,06	1,32	0,08
STM3523	glpR	0,99	0,03	0,74	0,02
STM3533		1,07	0,09	0,87	0,09
STM3543	gntR	1,73	0,05	0,58	0,02
STM3546	yhhY	4,54	0,00	0,70	0,07
STM3568	rpoH	1,00	0,10	0,49	0,01
STM3584	nikR	0,59	0,03	1,71	0,02
STM3602		0,39	0,05	0,89	0,09
STM3607	yhjC	1,16	0,04	1,42	0,05
STM3633		0,90	0,04	1,03	0,09
STM3643	yiaC	0,75	0,01	0,80	0,01
STM3648	yiaG	1,17	0,09	0,83	0,07
STM3651		0,71	0,05	0,34	0,02
STM3653		2,14	0,00	2,45	0,04
STM3662	xylR	0,36	0,02	1,25	0,07
STM3667	yiaJ	1,12	0,06	0,86	0,06
STM3678		1,03	0,09	1,21	0,05
STM3681		1,21	0,09	0,76	0,08
STM3687	mtlR	0,76	0,03	0,87	0,09
STM3693	lldR	1,05	0,07	0,68	0,03
STM3696		4,38	0,01	0,80	0,01
STM3732	ttk	1,49	0,05	0,47	0,04
STM3736		1,33	0,08	1,53	0,07
STM3741	rpoZ	1,42	0,02	1,40	0,08
STM3756	rmbA	0,98	0,09	1,23	0,06
STM3759	marT	1,81	0,06	0,75	0,07
STM3773		1,54	0,06	0,91	0,02
STM3778		1,09	0,09	1,16	0,03
STM3785		0,55	0,03	1,14	0,09
STM3794		0,31	0,04	0,99	0,10
STM3800	dsdC	1,09	0,08	1,28	0,08
STM3830	dgoR	2,19	0,00	2,03	0,04
STM3834		0,24	0,02	7,02	0,01
STM3848	yidZ	0,93	0,01	0,75	0,04

STM3876	asnC	1,05	0,07	5,57	0,01
STM3886	rbsR	0,71	0,03	0,63	0,06
STM3888	yieP	0,55	0,00	1,04	0,07
STM3897	yifA	0,87	0,03	0,90	0,07
STM3908	ilvY	0,86	0,09	0,37	0,04
STM3917	rho	0,95	0,08	0,46	0,01
STM3924	wecD	0,98	0,09	0,81	0,07
STM3964	metR	0,67	0,02	0,72	0,01
STM3977	rfaH	0,73	0,07	1,18	0,08
STM4025	yihW	1,47	0,05	0,55	0,01
STM4029	yiiD	1,51	0,02	1,33	0,07
STM4033				0,01	
STM4048	rhaS	0,86	0,03	1,23	0,08
STM4049	rhaR	0,66	0,01	0,70	0,01
STM4068		1,62	0,02	1,43	0,04
STM4073	ydeW	0,89	0,09	1,10	0,08
STM4094	cytR	1,22	0,01	0,71	0,03
STM4117	yijO	1,38	0,01	1,03	0,08
STM4125	oxyR	0,54	0,01	0,91	0,07
STM4127	yijC	0,96	0,09	0,56	0,01
STM4148	nusG	0,94	0,06	0,96	0,09
STM4165	rsd	0,87	0,01	0,75	0,02
STM4181	yjaB	0,89	0,04	0,93	0,07
STM4187	iclR	1,19	0,02	1,09	0,08
STM4237	lexA	0,76	0,01	0,97	0,09
STM4265	soxS	0,49	0,01	0,44	0,00
STM4266	soxR	0,92	0,08	1,02	0,09
STM4270		0,49	0,03	1,20	0,06
STM4287	phnO	1,28	0,01	0,63	0,03
STM4295	adiY	0,92	0,03	1,11	0,08
STM4297	melR	0,44	0,04	1,19	0,05
STM4303	dcuR	0,88	0,06	1,08	0,05
STM4314		0,79	0,01	1,44	0,07
STM4315		1,04	0,09	1,26	0,08
STM4318		3,63	0,02	0,84	0,05
STM4320		1,08	0,09	0,50	0,06
STM4322	yjdC	0,80	0,05	0,89	0,09
STM4337	ecnR	0,70	0,01	0,77	0,08
STM4367	yjeB	0,95	0,08	0,51	0,01
STM4368	vacB	3,03	0,03	0,13	0,01
STM4371	yjfJ	0,73	0,00	1,53	0,06
STM4381	yjfQ	0,82	0,08	1,39	0,06
STM4402	ytfH	1,01	0,10	0,52	0,02
STM4417		6,24	0,04	0,71	0,02
STM4423		8,13	0,04	1,24	0,03
STM4455	treR	0,33	0,05	0,16	0,01

STM4473	yjgM	0,94	0,07	1,10	0,09
STM4481	idnR	0,86	0,00	0,88	0,01
STM4507	uxuR	0,97	0,08	0,63	0,01
STM4511	yjiE	1,23	0,06	1,42	0,07
STM4580	nadR	3,51	0,02	0,18	0,01
STM4583	trpR	0,59	0,02	0,87	0,08
STM4586	rob	4,15	0,03	0,12	0,01

Unknown function

STM0005	yaaA	1,12	0,06	1,57	0,07
STM0009	yaaH	0,90	0,05	0,38	0,01
STM0010	htgA	0,97	0,06	0,77	0,04
STM0098		0,99	0,10	0,65	0,02
STM0105	yabI	0,87	0,04	2,11	0,00
STM0119	yabB	1,55	0,03	1,46	0,07
STM0139	yacF	0,90	0,02	0,61	0,01
STM0160	yacL	0,87	0,02	1,07	0,08
STM0162		1,15	0,08	1,39	0,07
STM0204	yadR	0,91	0,05	0,68	0,03
STM0205	yadS	0,94	0,08	0,79	0,04
STM0239	yaeQ	1,71	0,06	15,11	0,00
STM0243	yaeB	1,08	0,09	4,49	0,01
STM0258	yafD	1,07	0,07	0,80	0,05
STM0266		0,62	0,04	1,74	0,07
STM0267		0,84	0,02	1,34	0,07
STM0269		0,32	0,04	1,29	0,07
STM0273		0,89	0,05	0,72	0,02
STM0274		0,85	0,06	0,67	0,03
STM0276		1,01	0,10	1,09	0,07
STM0279		0,73	0,01	1,62	0,06
STM0280		0,74	0,06	0,58	0,07
STM0281		3,34	0,02	0,17	0,01
STM0282		0,88	0,06	0,68	0,03
STM0287		0,77	0,02	0,78	0,02
STM0289		1,11	0,08	1,17	0,09
STM0312	yafK	1,21	0,05	1,41	0,08
STM0381		1,49	0,01	0,64	0,01
STM0387	yaiI	4,03	0,03	2,27	0,02
STM0391	yaiE	1,55	0,00	0,57	0,01
STM0403	yajB	0,54	0,01	0,91	0,09
STM0435	yajQ	0,94	0,08	1,38	0,08
STM0465	ybaY	1,17	0,08	0,76	0,08
STM0482	ybaN	0,36	0,00	5,10	0,01
STM0485	ybaB	3,82	0,02	0,16	0,01
STM0495	ybaK	0,11	0,02	0,71	0,08
STM0496	ybaP	0,97	0,08	0,75	0,01

STM0535	lpxH	0,60	0,07	3,44	0,04
STM0538		1,62	0,03	8,98	0,00
STM0541	ybcJ	0,47		2,52	0,00
STM0559	rfbI	1,05	0,08	1,43	0,01
STM0566		1,15	0,08	1,28	0,08
STM0579	ybdF	0,24	0,03	1,63	0,06
STM0583	ybdK	0,04	0,01	1,26	0,03
STM0587	ybdZ	0,14		1,02	0,10
STM0636	ybeD	1,03	0,07	0,62	0,02
STM0641	ybeA	1,35	0,01	1,45	0,02
STM0642	ybeB	1,43	0,00	0,83	0,06
STM0711	ybgI	3,32	0,03	0,15	0,01
STM0742	ybgT	0,66	0,01	0,65	0,00
STM0743	ybgE	0,85	0,02	1,23	0,04
STM0750	ybgF	0,85	0,03	0,86	0,05
STM0777		0,81	0,03	1,84	0,03
STM0800	slrP	2,92	0,00	0,50	0,04
STM0801	ybhK	0,25	0,00	2,20	0,01
STM0811	ybhN	0,80	0,03	1,03	0,09
STM0837	ybiS	1,73	0,00	0,56	0,06
STM0869		2,03	0,00	2,18	0,04
STM0884		2,07	0,00	2,16	0,04
STM0918					
STM0919					
STM0921					
STM0923					
STM0925					
STM0930	orfB	1,04	0,09	1,38	0,08
STM0938	ybjE	0,69	0,02	0,72	0,06
STM0940	ybjX	1,09	0,09	0,90	0,09
STM0944	yljA	1,18	0,06	1,40	0,08
STM0975	ycaO	6,09	0,03	1,11	0,09
STM0976	ycaP	0,97	0,10	1,13	0,09
STM0986	ycaQ	0,48	0,02	0,43	0,04
STM0987	ycaR	2,18	0,03	0,14	0,01
STM0990	ycbC	0,91	0,08	0,27	0,02
STM0995	ycbB	0,81	0,05	0,82	0,08
STM0996	ycbK	1,11	0,06	0,57	0,01
STM1041		0,52	0,00	6,43	0,01
STM1042		1,16	0,03	1,06	0,09
STM1045		1,85	0,03	0,82	0,02
STM1047		1,01	0,10	2,38	0,05
STM1048		1,03	0,10	0,52	0,05
STM1053		1,03	0,08	1,04	0,10
STM1063	pqiA	1,85	0,00	0,93	0,07
STM1065	ymbA	1,09	0,05	1,07	0,01

STM1069	ycbG	3,66	0,00	0,68	0,02
STM1073	yccS	0,80	0,04	0,44	0,01
STM1074	yccF	1,54	0,01	1,01	0,09
STM1077	yccT	0,42	0,02	1,37	0,06
STM1079	yccV	0,96	0,04	0,70	0,04
STM1088	pipB	6,91	0,00	0,66	0,03
STM1103	hpaD	1,06	0,07	2,11	0,05
STM1123		3,19	0,02	0,11	0,00
STM1130		0,91	0,04	2,56	0,05
STM1157	yceI	0,94	0,09	0,15	0,01
STM1168	yceH	0,68	0,05	0,80	0,07
STM1212	ycfJ	0,16		0,63	0,06
STM1215	ycfS	0,14	0,00	0,61	0,06
STM1229	ycfD	1,28	0,04	1,37	0,07
STM1236		0,82	0,05	0,60	0,07
STM1274	yeaQ	0,84	0,01	0,70	0,01
STM1277	yeaO	1,15	0,07	1,33	0,08
STM1280	yeaL	0,59	0,03	1,29	0,05
STM1282	yeaK	1,38	0,07	5,31	0,01
STM1284	yeaH	1,63	0,02	1,10	0,09
STM1317	celG	0,19	0,02	0,24	0,02
STM1324		0,94	0,08	1,29	0,08
STM1328		1,62	0,06	0,86	0,08
STM1345	ydiU	1,09	0,05	1,08	0,09
STM1348	ydiA	0,66	0,02	1,74	0,06
STM1369	sufA	0,46	0,05	1,50	0,06
STM1375	ynhG	0,58	0,02	0,78	0,08
STM1389	orf319	1,29	0,07	0,64	0,01
STM1441		2,37	0,04	0,98	0,10
STM1466	ydgA	1,05	0,09	1,42	0,05
STM1504	ynfA	0,88	0,04	0,74	0,01
STM1515	ydeI	0,33	0,01	0,33	0,03
STM1527		1,93	0,01	1,47	0,02
STM1573		0,58	0,01	0,75	0,08
STM1586		1,72	0,01	0,82	0,09
STM1591	ydcZ	3,24	0,03	0,12	0,01
STM1594	srfB	1,05	0,08	1,19	0,06
STM1595	srfC	0,35	0,00	1,36	0,05
STM1621		0,76	0,01	1,76	0,03
STM1624		1,16	0,03	0,72	0,00
STM1628				1,18	0,02
STM1632		2,00	0,03	0,56	0,02
STM1637		3,30	0,02	0,13	0,01
STM1640	ydcF	1,39	0,07	0,69	0,07
STM1644	ydbL	4,24	0,02	1,63	0,05
STM1658	ydaL	1,04	0,08	0,42	0,00

STM1665		3,58	0,02	0,15	0,01
STM1684	ycjF	1,03	0,09	1,35	0,08
STM1701	yciW	3,88	0,02	0,23	0,01
STM1709	yciS	0,95	0,07	0,61	0,03
STM1729	yciF	1,15	0,03	0,58	0,01
STM1730	yciE	2,57	0,04	0,73	0,07
STM1738	yciI	1,03	0,09	1,20	0,09
STM1740	yciU	0,83	0,00	0,71	0,02
STM1755	ychJ	0,93	0,06	0,70	0,03
STM1773	ychA	1,11	0,01	0,66	0,00
STM1774	sirC	2,31	0,01	4,50	0,01
STM1797	ymgE	0,82	0,00	0,56	0,00
STM1804	ycgB	0,98	0,09	0,57	0,00
STM1811	ycgN	1,55	0,02	1,43	0,07
STM1813	ycgL	1,37	0,03	1,45	0,07
STM1823	yoaH	0,92	0,09	1,37	0,02
STM1833		0,72	0,05	2,54	0,01
STM1834	yebN	0,23	0,03	2,23	0,02
STM1848	yebS	1,17	0,00	0,86	0,03
STM1880	yebE	1,21	0,02	0,44	0,03
STM1882	yebG	2,99	0,01	0,46	0,04
STM1899	yebC	1,01	0,10	0,85	0,07
STM1903	yecE	1,12	0,04	1,30	0,04
STM1908	yecM	0,39	0,05	1,28	0,01
STM1989	yedI	0,24	0,00	7,03	0,02
STM2001	yeeI	0,74	0,01	0,63	0,02
STM2015	erfK	1,21	0,02	0,69	0,00
STM2050	pduO	0,81	0,04	0,70	0,07
STM2059	yeeX	0,93	0,05	0,60	0,01
STM2060	yeeA	0,96	0,05	0,88	0,06
STM2156	yehR	0,81	0,06	0,89	0,09
STM2157	yehS	1,13	0,06	1,57	0,05
STM2170	yohD	0,71	0,05	0,68	0,01
STM2184	sanA	1,17	0,03	0,72	0,03
STM2192	yeiB	0,77	0,07	0,93	0,09
STM2202	yeiH	1,37	0,01	2,15	0,04
STM2227	yejL	1,20	0,06	0,82	0,06
STM2241	sspH2	0,73	0,05	1,02	0,10
STM2311	elaB	0,76	0,06	0,77	0,08
STM2335	yfbU	0,99	0,09	0,84	0,01
STM2336		0,95	0,08	0,61	0,00
STM2339	yfcC	0,74	0,04	0,38	0,00
STM2342		0,82	0,03	0,84	0,02
STM2364	dedD	0,74	0,01	0,89	0,07
STM2367	dedA	0,69	0,02	0,62	0,02
STM2379		0,40	0,03	1,05	0,08

STM2381	yfcM	1,65	0,00	0,88	0,08
STM2386	yfcN	0,92	0,03	1,34	0,01
STM2390	yfcZ	1,68	0,06	0,68	0,01
STM2520	yfgL	1,18	0,04	1,28	0,08
STM2521	yfgM	0,90	0,06	0,59	0,01
STM2524	yfgA	0,73	0,05	0,94	0,09
STM2537	yfhJ	0,91	0,07	1,44	0,07
STM2541	yfhF	1,02	0,08	0,71	0,03
STM2590		2,23	0,00	1,96	0,05
STM2592		0,73	0,02	0,80	0,02
STM2593		0,79	0,04	0,70	0,02
STM2594		0,14	0,00	6,86	0,01
STM2650	yfiP	1,01	0,08	0,89	0,04
STM2661	yfiH	0,87	0,03	0,86	0,06
STM2686	yfjF	0,97	0,09	1,51	0,07
STM2697					
STM2780		1,65	0,00	0,71	0,08
STM2781	virK	1,31	0,00	0,88	0,06
STM2788					
STM2795	ygaU	1,37	0,02	1,03	0,10
STM2796	yqaE	1,22	0,09	0,13	0,00
STM2802	ygaM	0,81	0,06	0,78	0,04
STM2804		1,18	0,01	0,58	0,02
STM2819	yqaA	1,69	0,01	0,94	0,09
STM2904		0,75	0,06	0,54	0,04
STM2917	ygbK	1,17	0,07	1,23	0,08
STM2928	ygbO	0,73	0,04	0,85	0,09
STM2965	yqcC	0,30	0,03	2,01	0,02
STM2968	yqcD	1,48	0,02	1,73	0,04
STM2981	ygdD	0,81	0,04	0,85	0,05
STM3021		0,41	0,02	1,62	0,04
STM3023	yohL	0,37	0,04	0,83	0,04
STM3034		0,91	0,05	0,52	0,01
STM3047	ygfY	1,20	0,03	0,82	0,03
STM3050	yqfB	0,66	0,03	0,84	0,08
STM3060	ygfE	1,11	0,00	0,78	0,04
STM3065	yggE	0,98	0,08	0,78	0,03
STM3092	sprT	7,31	0,00	0,60	0,06
STM3094	yggJ	1,06	0,09	0,74	0,01
STM3101	yggT	0,98	0,09	0,79	0,03
STM3102	yggU	0,84	0,09	5,77	0,02
STM3108	yggL	0,86	0,09	0,87	0,09
STM3111	yggX	0,92	0,06	0,65	0,02
STM3131		0,93	0,06	2,55	0,05
STM3153	yqhA	0,96	0,08	1,04	0,09
STM3162	yghB	0,90	0,04	0,87	0,07

STM3176	ygiW	1,42	0,03	1,37	0,07
STM3180	ygiN	1,15	0,08	0,70	0,00
STM3184	yqiB	0,65	0,00	1,34	0,05
STM3189	ygiD	10,93	0,01	2,18	0,04
STM3196	yqiC	0,91	0,06	0,66	0,01
STM3199	yqiK	1,07	0,09	1,97	0,00
STM3202	ygiF	0,87	0,04	0,40	0,03
STM3207	ygiH	1,22	0,06	0,29	0,01
STM3222	ygjQ	3,39	0,02	0,18	0,01
STM3226	yqjA	2,39	0,03	0,17	0,02
STM3229	yqjD	1,49	0,03	1,13	0,09
STM3230	yqjE	0,88	0,07	0,85	0,09
STM3232	yqjF	0,73	0,01	1,75	0,05
STM3234	yhaH	0,83	0,00	0,78	0,03
STM3238	yhaN	0,63	0,03	0,64	0,07
STM3270	yhbP	0,70	0,05	1,41	0,06
STM3277		1,29	0,07	0,68	0,02
STM3288	yhbC	1,52	0,02	1,33	0,08
STM3317	yrbK	1,22	0,03	1,00	0,10
STM3318	yhbN	1,13	0,05	1,50	0,07
STM3332	yhcG	1,58	0,06	2,21	0,04
STM3347	yhcB	1,03	0,09	1,20	0,08
STM3364	yhcP	0,24	0,03	0,45	0,04
STM3369	yhdP	0,96	0,10	19,69	0,00
STM3378		1,07	0,07	1,21	0,08
STM3381	yhdT	0,83	0,05	9,46	0,02
STM3404	smg	1,52	0,03	1,22	0,08
STM3411		0,75	0,00	1,28	0,07
STM3452	yheO	1,06	0,08	0,99	0,10
STM3454	slyX	0,98	0,08	0,78	0,05
STM3463	yheU	2,46	0,00	0,95	0,08
STM3467	yhfK	0,94	0,07	0,86	0,07
STM3485	damX	1,02	0,06	0,75	0,02
STM3516		0,38	0,04	3,32	0,03
STM3519	rtcB	0,17	0,02	0,74	0,08
STM3549		4,51	0,02	0,16	0,01
STM3573	yhhL	0,32	0,07	5,86	0,01
STM3575	yhhN	4,19	0,02	0,47	0,02
STM3579	yhhQ	0,88	0,09	5,94	0,00
STM3608	yhjD	2,28	0,06	0,78	0,08
STM3652		3,08	0,04	0,58	0,05
STM3658	yiaH	0,83	0,03	1,85	0,03
STM3659	yiaB	2,83	0,04	1,48	0,07
STM3679		1,02	0,09	1,10	0,09
STM3706	yigQ	1,12	0,01	0,62	0,04
STM3735	yicC	0,95	0,07	0,66	0,01

STM3738	yigC	1,19	0,02	0,71	0,02
STM3764	mgtC	0,77	0,05	2,81	0,04
STM3777		1,01	0,10	0,86	0,08
STM3805	yidH	2,09	0,00	0,59	0,05
STM3841		2,86	0,01	3,42	0,01
STM3898	yifE	0,92	0,04	0,93	0,09
STM3902	ilvM	1,08	0,06	1,00	0,10
STM3906		0,86	0,01	1,31	0,05
STM3948	yigA	0,62	0,02	0,70	0,01
STM3969	yigN	0,84	0,05	0,96	0,01
STM3971	yigP	3,01	0,03	0,20	0,01
STM3985	yigZ	0,75	0,07	0,96	0,10
STM3995	yihD	1,30	0,05	1,62	0,06
STM4003	yihI	1,08	0,03	0,97	0,09
STM4027	rbn	1,04	0,09	1,27	0,08
STM4031		0,90	0,05	0,64	0,03
STM4043	yiiL	3,26	0,02	0,75	0,03
STM4056	yiiM	0,75	0,00	0,88	0,02
STM4071		0,18	0,00	1,66	0,06
STM4079	yneC	18,00	0,00	1,68	0,05
STM4083	yiiR	0,99	0,09	0,99	0,08
STM4088	yiiU	0,94	0,06	0,48	0,01
STM4107	yijF	3,66	0,02	0,14	0,01
STM4169	yjaG	0,87	0,05	0,67	0,01
STM4186		3,84	0,02	0,25	0,02
STM4192		1,19	0,00	1,23	0,06
STM4206		0,75	0,01	1,70	0,03
STM4226	yjbA	1,44	0,02	0,76	0,06
STM4240	yjbJ	0,36	0,01	1,15	0,04
STM4250	yjbQ	1,08	0,02	0,75	0,05
STM4251	yjbR	0,83	0,01	0,80	0,04
STM4274	yjcH	1,08	0,08	1,09	0,08
STM4288	phnB	2,65	0,04	0,47	0,04
STM4310		0,60	0,04	3,58	0,03
STM4317		1,07	0,08	1,27	0,08
STM4356	yjeF	3,84	0,01	1,82	0,01
STM4365	yjeT	1,19	0,05	0,64	0,06
STM4370	yjfI	0,53	0,03	1,63	0,07
STM4374	yjfL	3,66	0,02	0,14	0,01
STM4383	sgaT	3,38	0,02	0,13	0,01
STM4411	ytfP	0,92	0,03	0,84	0,06
STM4429		3,80	0,02	0,14	0,01
STM4437	yjgA	1,33	0,04	1,54	0,06
STM4464		1,62	0,07	0,61	0,05
STM4470	yjgD	0,96	0,05	0,68	0,04
STM4474	yjgN	1,05	0,08	1,63	0,07

STM4490		0,86	0,04	0,57	0,01
STM4499	yeeN	1,07	0,07	0,92	0,08
STM4501		0,03		60,86	
STM4502		1,06	0,08	1,14	0,07
STM4505		0,55	0,06	1,46	0,06
STM4509		1,75	0,05	1,00	0,10
STM4513	yjiG	0,53	0,01	0,48	0,00
STM4514	yjiH	1,05	0,07	0,58	0,01
STM4516	yjiN	2,08	0,06	2,56	0,02
STM4528		0,66	0,01	1,06	0,09
STM4529		0,64	0,01	1,36	0,05
STM4531	yjiX	0,37	0,02	1,10	0,05
STM4545		0,27	0,02	1,16	0,08
STM4546	yjjP	1,13	0,08	1,73	0,06
STM4549		1,66	0,05	0,80	0,08
STM4584	yjjX	0,58	0,06	1,16	0,09
STM4587	creA	1,29	0,07	0,85	0,09

Supplementary Table S3 legend:

Supplementary Table S3: Microarray analysis. Shown is the effect of cortisol on gene expression of a stationary phase culture of *Salmonella* Typhimurium WT and Δ*scsA*, respectively, during conditions mimicking the intracellular environment. Expression of the untreated control WT group and Δ*scsA* is set to 1. Genes differentially regulated by fold < 0.5 or > 2.0 and with P ≤ 0.05 are represented in black letters. Grey letters indicate non-significant changes or changes with a fold > 0.5 or < 2.0. Microarray data have been deposited in the Gene Expression Omnibus at NCBI with series accession numbers GSE55430.
